# Spatial distribution of Madeira Island Laurisilva endemic spiders (Arachnida: Araneae)

**DOI:** 10.3897/BDJ.2.e1051

**Published:** 2014-02-14

**Authors:** Luís C. Crespo, Mário Boieiro, Pedro Cardoso, Carlos A.S. Aguiar, Isabel R. Amorim, Carla Barrinha, Paulo A.V. Borges, Dília Menezes, Fernando Pereira, Carla Rego, Sérvio Ribeiro, Israel F. Silva, Artur R.M. Serrano

**Affiliations:** † Centro de Biologia Ambiental/PEERS Faculdade de Ciências da Universidade de Lisboa, Ed. C2, 2º Piso, Campo Grande, Lisboa, Portugal; ‡ Finnish Museum of Natural History, Helsinki, Finland; § Grupo de Biodiversidade dos Açores, (CITA-A)/PEERS Departamento de Ciências Agrárias, Universidade dos Açores, Pico da Urze, Angra do Heroísmo, Portugal; | Parque Natural da Madeira, Qta. do Bom Sucesso, Caminho do Meio, Funchal, Portugal; ¶ Lab. Evolutionary Ecology of Canopy Insects, Department of Biodiversity, Evolution and Environment, Instituto de Ciências Exatas e Biológicas, Universidade Federal de Ouro Preto, Ouro Preto, Brazil

**Keywords:** Macaronesia, terrestrial arthropods, island endemics, laurel forest, biodiversity hotspot

## Abstract

Madeira island presents a unique spider diversity with a high number of endemic species, many of which are still poorly known. A recent biodiversity survey on the terrestrial arthropods of the native forest, Laurisilva, provided a large set of standardized samples from various patches throughout the island. Out of the fifty two species recorded, approximately 33.3% are Madeiran endemics, many of which had not been collected since their original description. Two new species to science are reported – *Ceratinopsis* n. sp. and *Theridion* n. sp. – and the first records of *Poeciloneta variegata* (Blackwall, 1841) and *Tetragnatha intermedia* Kulczynski, 1891 are reported for the first time for Madeira island. Considerations on species richness and abundance from different Laurisilva locations are presented, together with distribution maps for endemic species. These results contribute to a better understanding of spider diversity patterns and endemic species distribution in the native forest of Madeira island.

## Introduction

Interest on Madeira spider fauna started almost two centuries ago (Blackwall 1859, Blackwall 1862, Bösenberg 1895, Bristowe 1925, Denis 1962, Denis 1963, Denis 1964, Johnson 1863, Kulczynski 1899, Kulczynski 1905, Lowe 1832, Schenkel 1938, Schmitz 1895, Warburton 1892) and has continued until recently, with the description of several new species (Wunderlich 1987, Wunderlich 1992, Wunderlich 1995). Despite the major relevance for the knowledge of Madeiran spider biodiversity, these taxonomic studies are based on traditional *ad-hoc* manner, without a standardized methodology, which renders them inappropriated to explore species distribution patterns. In other words, although the *ad-hoc* approach may be useful in targeting specific localities or ecological niches by experienced collectors (Cardoso et al. 2009), it does not capture the real distribution of species. The lack of knowledge on species distribution is now referred to as the Wallacean shortfall (Lomolino 2004; see Cardoso et al. 2011), and the main objective of this contribution is to help to overcome this shortfall for Madeiran spiders associated with the native laurel forest – Laurisilva.

Laurisilva is a relict evergreen forest that covered part of the Mediterranean Basin during the Tertiary, being restricted nowadays to a few locations in the Macaronesian archipelagoes of the Azores, Madeira and Canary Islands, and in some secluded sites of North Africa (Neves et al. 1996). In the archipelago of Madeira, Laurisilva is restricted to the main island (Madeira), covering about 16000 ha and harbouring a large number of endemic fungi, plants and animals (Uva 2008; Borges et al. 2008). Taking in consideration the high natural value of this forest type, now internationally recognized (IUCN 1999, Myers et al. 2000), local entities created the Natural Park of Madeira with the aim of valuing, studying and protecting this unique ecosystem.

The latest spider checklist (Cardoso and Crespo 2008) reports the occurrence of 164 species for Madeira island. Among these species, 40 are considered single-island endemics (SIE) and some belong to genera that radiated in Madeira archipelago (e.g. *Dysdera* Latreille, 1804, *Hogna* Simon, 1885, *Pholcus* Walckenaer, 1805). Approximatelly 63% of the SIEs were collected exclusively from Laurisilva sites, strongly suggesting an intimate association between these species and this type of habitat. Although the Natural Park of Madeira has developed major efforts to protect and restore Laurisilva patches from human activities and invasive species, conservation measures specifically targeting endemic terrestrial arthropod fauna are yet to be put forward. Preliminary conservation priorities for the endemic invertebrate fauna have already been identified using taxonomically unbiased criteria (Martín et al. 2010, Martín et al. 2008), but there is still a lack of baseline information for most species. The collection of species distribution and abundance data using standardized sampling is paramount to assess species conservation status. The aim of our study is therefore to provide information on the distribution of spiders endemic to the Laurisilva of Madeira island based on a large-scale assessment using standardized sampling.

## Materials and methods

Spiders were sampled using two standardized complementary techniques: pitfall trapping and direct sampling. Pitfall sampling was performed both in May-June 2006 (sites 1 to 28) and June 2007 (sites 29 to 36). A linear transect with 30 pitfall traps, 5 m apart, was set in each study site. Each trap consisted of a plastic cup (4.2 cm diameter by 7.8 cm height) protected with a plastic cover (15 cm diameter) attached ~3 cm aboveground to prevent flooding and loss of specimens. Ethyleneglicol (10%) and Turquin solution were used as preservatives in alternate traps along the linear transect (BALA protocol, Borges et al. 2006, Borges et al. 2005). The traps were active during a two-week period.

Direct sampling was performed in June 2007 for most sampling sites. The sampling design was semi-quantitative with a sampling unit defined as 30 minute/person of effective fieldwork. Direct sampling included the search of spiders in three microhabitats (under bark of dead tree trunks, mosses and lichens, on living tree trunks and under stones/leaf litter) with the collection of six replicates/microhabitat/site (total 18 samples per site).

For each spider species collected, we recorded information on sampling technique used, site location code (Table 1), number of captured specimens and gender whenever possible. Information on species distribution follows the World Spider Catalog (Platnick 2013), updated with records from regional checklists of the Macaronesian archipelagoes (Cardoso and Crespo 2008, Cardoso et al. 2010, Macías 2009). Furthermore, distribution maps are provided for each endemic species found in the Laurisilva of Madeira island (Figs 1, 2, 3, 4, 5). The specimens collected are deposited in the entomological collection of the Faculty of Sciences of the University of Lisbon (Portugal).

## Faunistic results

### Laurisilva of Madeira

#### Natura code

PTMAD0001, PTMAD0002

#### Description

The study took place in Madeira island located in the Atlantic Ocean, approximately 1000 km from the Iberian Peninsula. Sampling was conducted in thirty-six sites (Table 1), mostly in Laurisilva patches, the only exceptions being two sites on an alpine meadow near Pico do Areeiro, one of the highest mountains on the island. Laurisilva sites were mostly located in pristine or near pristine forest patches (Figs 1a, 6) where the dominant tree species included some of the following Madeiran or Macaronesian endemics: *Clethra arborea*, *Erica platycodon maderincola*, *Laurus novocanariensis*, *Ocotea foetens* and *Persea indica*. Additional information on plant species composition and conservation status of sampling sites can be found in Neves et al. 1996.

#### Zygiella minima

Schmidt, 1968

##### Materials

- **Type status:** Other material. **Occurrence:** sex: 1 female; **Location:** locationID: 33; higherGeography: Macaronesia; continent: Europe; waterBody: Atlantic Ocean; islandGroup: Madeira archipelago; island: Madeira; country: Portugal; countryCode: PT; stateProvince: Madeira; county: Porto Moniz; locality: Rabaçal; verbatimElevation: 930; decimalLatitude: 32.7647; decimalLongitude: -17.1341; geodeticDatum: WGS84; **Event:** samplingProtocol: Direct sampling

##### Ecological interactions

###### Native status

macaronesian endemic

##### Distribution

Canary Islands, Deserta Grande island, Madeira island

##### Notes

This species was recently found for the first time in Madeira archipelago in an erosion cave of Deserta Grande island (Crespo et al. 2013), and its distribution has been now extended by this study to Madeira island.

#### Clubiona decora

Blackwall, 1859

##### Materials

- **Type status:** Other material. **Occurrence:** sex: 1 male; **Location:** locationID: 1; higherGeography: Macaronesia; continent: Europe; waterBody: Atlantic Ocean; islandGroup: Madeira archipelago; island: Madeira; country: Portugal; countryCode: PT; stateProvince: Madeira; county: Machico; locality: Funduras; verbatimElevation: 500; decimalLatitude: 32.7493; decimalLongitude: -16.8114; geodeticDatum: WGS84; **Event:** samplingProtocol: Direct sampling
- **Type status:** Other material. **Occurrence:** sex: 1 male; **Location:** locationID: 6; higherGeography: Macaronesia; continent: Europe; waterBody: Atlantic Ocean; islandGroup: Madeira archipelago; island: Madeira; country: Portugal; countryCode: PT; stateProvince: Madeira; county: Santana; locality: Fajã da Nogueira - Mtdo. do Leacoque; verbatimElevation: 614; decimalLatitude: 32.7418; decimalLongitude: -16.9177; geodeticDatum: WGS84; **Event:** samplingProtocol: Direct sampling
- **Type status:** Other material. **Occurrence:** sex: 1 male; **Location:** locationID: 7; higherGeography: Macaronesia; continent: Europe; waterBody: Atlantic Ocean; islandGroup: Madeira archipelago; island: Madeira; country: Portugal; countryCode: PT; stateProvince: Madeira; county: Santana; locality: Fajã da Nogueira - Tanque; verbatimElevation: 845; decimalLatitude: 32.7425; decimalLongitude: -16.9168; geodeticDatum: WGS84; **Event:** samplingProtocol: Direct sampling
- **Type status:** Other material. **Occurrence:** sex: 1 female; **Location:** locationID: 11; higherGeography: Macaronesia; continent: Europe; waterBody: Atlantic Ocean; islandGroup: Madeira archipelago; island: Madeira; country: Portugal; countryCode: PT; stateProvince: Madeira; county: Santana; locality: Achada do Teixeira; verbatimElevation: 1211; decimalLatitude: 32.7733; decimalLongitude: -16.9081; geodeticDatum: WGS84; **Event:** samplingProtocol: Direct sampling
- **Type status:** Other material. **Occurrence:** sex: 1 male, 2 females; **Location:** locationID: 18; higherGeography: Macaronesia; continent: Europe; waterBody: Atlantic Ocean; islandGroup: Madeira archipelago; island: Madeira; country: Portugal; countryCode: PT; stateProvince: Madeira; county: Porto Moniz; locality: Chão da Ribeira; verbatimElevation: 491; decimalLatitude: 32.7957; decimalLongitude: -17.1117; geodeticDatum: WGS84; **Event:** samplingProtocol: Direct sampling
- **Type status:** Other material. **Occurrence:** sex: 1 female; **Location:** locationID: 24; higherGeography: Macaronesia; continent: Europe; waterBody: Atlantic Ocean; islandGroup: Madeira archipelago; island: Madeira; country: Portugal; countryCode: PT; stateProvince: Madeira; county: Porto Moniz; locality: Fanal - Levada dos Cedros; verbatimElevation: 820; decimalLatitude: 32.8259; decimalLongitude: -17.158; geodeticDatum: WGS84; **Event:** samplingProtocol: Direct sampling
- **Type status:** Other material. **Occurrence:** sex: 1 female; **Location:** locationID: 25; higherGeography: Macaronesia; continent: Europe; waterBody: Atlantic Ocean; islandGroup: Madeira archipelago; island: Madeira; country: Portugal; countryCode: PT; stateProvince: Madeira; county: Porto Moniz; locality: Fanal; verbatimElevation: 890; decimalLatitude: 32.8236; decimalLongitude: -17.156; geodeticDatum: WGS84; **Event:** samplingProtocol: Direct sampling
- **Type status:** Other material. **Occurrence:** sex: 1 female; **Location:** locationID: 32; higherGeography: Macaronesia; continent: Europe; waterBody: Atlantic Ocean; islandGroup: Madeira archipelago; island: Madeira; country: Portugal; countryCode: PT; stateProvince: Madeira; county: Calheta; locality: Rabaças; verbatimElevation: 993; decimalLatitude: 32.7413; decimalLongitude: -17.0783; geodeticDatum: WGS84; **Event:** samplingProtocol: Direct sampling
- **Type status:** Other material. **Occurrence:** sex: 1 female; **Location:** locationID: 33; higherGeography: Macaronesia; continent: Europe; waterBody: Atlantic Ocean; islandGroup: Madeira archipelago; island: Madeira; country: Portugal; countryCode: PT; stateProvince: Madeira; county: Porto Moniz; locality: Rabaçal; verbatimElevation: 930; decimalLatitude: 32.7647; decimalLongitude: -17.1341; geodeticDatum: WGS84; **Event:** samplingProtocol: Direct sampling

##### Ecological interactions

###### Native status

native

##### Distribution

Madeira archipelago, Azores, Balkans

##### Notes

Its presence in laurel forest suggests being a native species and its occurence in the Balkans needs to be confirmed. In the Azores the species is very common in the laurel forest and in the canopies of low altitude orchards and exotic trees.

#### Lathys affinis

(Blackwall, 1862)

##### Materials

- **Type status:** Other material. **Occurrence:** sex: 1 female; **Location:** locationID: 4; higherGeography: Macaronesia; continent: Europe; waterBody: Atlantic Ocean; islandGroup: Madeira archipelago; island: Madeira; country: Portugal; countryCode: PT; stateProvince: Madeira; county: Santana; locality: Fajã da Nogueira - Mtdo. do Leacoque; verbatimElevation: 630; decimalLatitude: 32.7415; decimalLongitude: -16.9161; geodeticDatum: WGS84; **Event:** samplingProtocol: Direct sampling
- **Type status:** Other material. **Occurrence:** sex: 1 female; **Location:** locationID: 6; higherGeography: Macaronesia; continent: Europe; waterBody: Atlantic Ocean; islandGroup: Madeira archipelago; island: Madeira; country: Portugal; countryCode: PT; stateProvince: Madeira; county: Santana; locality: Fajã da Nogueira - Mtdo. do Leacoque; verbatimElevation: 614; decimalLatitude: 32.7418; decimalLongitude: -16.9177; geodeticDatum: WGS84; **Event:** samplingProtocol: Direct sampling
- **Type status:** Other material. **Occurrence:** sex: 1 female; **Location:** locationID: 7; higherGeography: Macaronesia; continent: Europe; waterBody: Atlantic Ocean; islandGroup: Madeira archipelago; island: Madeira; country: Portugal; countryCode: PT; stateProvince: Madeira; county: Santana; locality: Fajã da Nogueira - Tanque; verbatimElevation: 845; decimalLatitude: 32.7425; decimalLongitude: -16.9168; geodeticDatum: WGS84; **Event:** samplingProtocol: Direct sampling
- **Type status:** Other material. **Occurrence:** sex: 1 male, 4 females; **Location:** locationID: 11; higherGeography: Macaronesia; continent: Europe; waterBody: Atlantic Ocean; islandGroup: Madeira archipelago; island: Madeira; country: Portugal; countryCode: PT; stateProvince: Madeira; county: Santana; locality: Achada do Teixeira; verbatimElevation: 1211; decimalLatitude: 32.7733; decimalLongitude: -16.9081; geodeticDatum: WGS84; **Event:** samplingProtocol: Direct sampling
- **Type status:** Other material. **Occurrence:** sex: 1 female; **Location:** locationID: 12; higherGeography: Macaronesia; continent: Europe; waterBody: Atlantic Ocean; islandGroup: Madeira archipelago; island: Madeira; country: Portugal; countryCode: PT; stateProvince: Madeira; county: Santana; locality: Achada do Teixeira; verbatimElevation: 1103; decimalLatitude: 32.7762; decimalLongitude: -16.9022; geodeticDatum: WGS84; **Event:** samplingProtocol: Direct sampling
- **Type status:** Other material. **Occurrence:** sex: 1 male, 2 females; **Location:** locationID: 13; higherGeography: Macaronesia; continent: Europe; waterBody: Atlantic Ocean; islandGroup: Madeira archipelago; island: Madeira; country: Portugal; countryCode: PT; stateProvince: Madeira; county: Santana; locality: Ribeiro Frio - Viveiro; verbatimElevation: 906; decimalLatitude: 32.7354; decimalLongitude: -16.8864; geodeticDatum: WGS84; **Event:** samplingProtocol: Direct sampling
- **Type status:** Other material. **Occurrence:** sex: 1 female; **Location:** locationID: 14; higherGeography: Macaronesia; continent: Europe; waterBody: Atlantic Ocean; islandGroup: Madeira archipelago; island: Madeira; country: Portugal; countryCode: PT; stateProvince: Madeira; county: Santana; locality: Ribeiro Frio - Cottages; verbatimElevation: 994; decimalLatitude: 32.7319; decimalLongitude: -16.8861; geodeticDatum: WGS84; **Event:** samplingProtocol: Direct sampling
- **Type status:** Other material. **Occurrence:** sex: 5 females; **Location:** locationID: 18; higherGeography: Macaronesia; continent: Europe; waterBody: Atlantic Ocean; islandGroup: Madeira archipelago; island: Madeira; country: Portugal; countryCode: PT; stateProvince: Madeira; county: Porto Moniz; locality: Chão da Ribeira; verbatimElevation: 491; decimalLatitude: 32.7957; decimalLongitude: -17.1117; geodeticDatum: WGS84; **Event:** samplingProtocol: Direct sampling
- **Type status:** Other material. **Occurrence:** sex: 4 females; **Location:** locationID: 24; higherGeography: Macaronesia; continent: Europe; waterBody: Atlantic Ocean; islandGroup: Madeira archipelago; island: Madeira; country: Portugal; countryCode: PT; stateProvince: Madeira; county: Porto Moniz; locality: Fanal - Levada dos Cedros; verbatimElevation: 820; decimalLatitude: 32.8259; decimalLongitude: -17.158; geodeticDatum: WGS84; **Event:** samplingProtocol: Direct sampling
- **Type status:** Other material. **Occurrence:** sex: 1 male; **Location:** locationID: 24; higherGeography: Macaronesia; continent: Europe; waterBody: Atlantic Ocean; islandGroup: Madeira archipelago; island: Madeira; country: Portugal; countryCode: PT; stateProvince: Madeira; county: Porto Moniz; locality: Fanal - Levada dos Cedros; verbatimElevation: 820; decimalLatitude: 32.8259; decimalLongitude: -17.158; geodeticDatum: WGS84; **Event:** samplingProtocol: Pitfall
- **Type status:** Other material. **Occurrence:** sex: 1 female; **Location:** locationID: 25; higherGeography: Macaronesia; continent: Europe; waterBody: Atlantic Ocean; islandGroup: Madeira archipelago; island: Madeira; country: Portugal; countryCode: PT; stateProvince: Madeira; county: Porto Moniz; locality: Fanal; verbatimElevation: 890; decimalLatitude: 32.8236; decimalLongitude: -17.156; geodeticDatum: WGS84; **Event:** samplingProtocol: Direct sampling
- **Type status:** Other material. **Occurrence:** sex: 2 females; **Location:** locationID: 27; higherGeography: Macaronesia; continent: Europe; waterBody: Atlantic Ocean; islandGroup: Madeira archipelago; island: Madeira; country: Portugal; countryCode: PT; stateProvince: Madeira; county: Porto Moniz; locality: Fanal; verbatimElevation: 1023; decimalLatitude: 32.8182; decimalLongitude: -17.1521; geodeticDatum: WGS84; **Event:** samplingProtocol: Direct sampling
- **Type status:** Other material. **Occurrence:** sex: 2 females; **Location:** locationID: 28; higherGeography: Macaronesia; continent: Europe; waterBody: Atlantic Ocean; islandGroup: Madeira archipelago; island: Madeira; country: Portugal; countryCode: PT; stateProvince: Madeira; county: Porto Moniz; locality: Fanal; verbatimElevation: 1134; decimalLatitude: 32.8062; decimalLongitude: -17.1409; geodeticDatum: WGS84; **Event:** samplingProtocol: Direct sampling
- **Type status:** Other material. **Occurrence:** sex: 3 females; **Location:** locationID: 32; higherGeography: Macaronesia; continent: Europe; waterBody: Atlantic Ocean; islandGroup: Madeira archipelago; island: Madeira; country: Portugal; countryCode: PT; stateProvince: Madeira; county: Calheta; locality: Rabaças; verbatimElevation: 993; decimalLatitude: 32.7413; decimalLongitude: -17.0783; geodeticDatum: WGS84; **Event:** samplingProtocol: Direct sampling
- **Type status:** Other material. **Occurrence:** sex: 3 females; **Location:** locationID: 33; higherGeography: Macaronesia; continent: Europe; waterBody: Atlantic Ocean; islandGroup: Madeira archipelago; island: Madeira; country: Portugal; countryCode: PT; stateProvince: Madeira; county: Porto Moniz; locality: Rabaçal; verbatimElevation: 930; decimalLatitude: 32.7647; decimalLongitude: -17.1341; geodeticDatum: WGS84; **Event:** samplingProtocol: Direct sampling
- **Type status:** Other material. **Occurrence:** sex: 1 female; **Location:** locationID: 34; higherGeography: Macaronesia; continent: Europe; waterBody: Atlantic Ocean; islandGroup: Madeira archipelago; island: Madeira; country: Portugal; countryCode: PT; stateProvince: Madeira; county: Ponta do Sol; locality: Risco; verbatimElevation: 1048; decimalLatitude: 32.7608; decimalLongitude: -17.1256; geodeticDatum: WGS84; **Event:** samplingProtocol: Direct sampling

##### Ecological interactions

###### Native status

macaronesian endemic

##### Distribution

Madeira archipelago, Canary Islands

##### Notes

This macaronesian endemic is widespread in the Laurisilva of Madeira island. L. Crespo has examined the material cited as *Lathys affinis* from the Canary Islands, deposited at the Senckenberg Naturmuseum of Frankfurt, and questions their species affiliation. Additional taxonomic analyses are thus needed to clarify the taxonomic status of the Canary Islands specimens.

#### Nigma puella

(Simon, 1870)

##### Materials

- **Type status:** Other material. **Occurrence:** sex: 1 female; **Location:** locationID: 32; higherGeography: Macaronesia; continent: Europe; waterBody: Atlantic Ocean; islandGroup: Madeira archipelago; island: Madeira; country: Portugal; countryCode: PT; stateProvince: Madeira; county: Calheta; locality: Rabaças; verbatimElevation: 993; decimalLatitude: 32.7413; decimalLongitude: -17.0783; geodeticDatum: WGS84; **Event:** samplingProtocol: Pitfall

##### Ecological interactions

###### Native status

introduced

##### Distribution

Europe, Madeira archipelago, Azores, Canary Islands

#### Haplodrassus dalmatensis

(L. Koch, 1866)

##### Materials

- **Type status:** Other material. **Occurrence:** sex: 13 males, 4 females; **Location:** locationID: 15; higherGeography: Macaronesia; continent: Europe; waterBody: Atlantic Ocean; islandGroup: Madeira archipelago; island: Madeira; country: Portugal; countryCode: PT; stateProvince: Madeira; county: Santana; locality: Pico do Areeiro; verbatimElevation: 1533; decimalLatitude: 32.7231; decimalLongitude: -16.9109; geodeticDatum: WGS84; **Event:** samplingProtocol: Pitfall
- **Type status:** Other material. **Occurrence:** sex: 20 males, 19 females; **Location:** locationID: 16; higherGeography: Macaronesia; continent: Europe; waterBody: Atlantic Ocean; islandGroup: Madeira archipelago; island: Madeira; country: Portugal; countryCode: PT; stateProvince: Madeira; county: Santana; locality: Pico do Areeiro; verbatimElevation: 1594; decimalLatitude: 32.7287; decimalLongitude: -16.9202; geodeticDatum: WGS84; **Event:** samplingProtocol: Pitfall

##### Ecological interactions

###### Native status

native

##### Distribution

Palearctic, Ethiopia

#### Macarophaeus cultior

Kulczynski, 1899

##### Materials

- **Type status:** Other material. **Occurrence:** sex: 1 male; **Location:** locationID: 2; higherGeography: Macaronesia; continent: Europe; waterBody: Atlantic Ocean; islandGroup: Madeira archipelago; island: Madeira; country: Portugal; countryCode: PT; stateProvince: Madeira; county: Machico; locality: Funduras; verbatimElevation: 552; decimalLatitude: 32.754; decimalLongitude: -16.8099; geodeticDatum: WGS84; **Event:** samplingProtocol: Direct sampling
- **Type status:** Other material. **Occurrence:** sex: 1 male, 5 females; **Location:** locationID: 6; higherGeography: Macaronesia; continent: Europe; waterBody: Atlantic Ocean; islandGroup: Madeira archipelago; island: Madeira; country: Portugal; countryCode: PT; stateProvince: Madeira; county: Santana; locality: Fajã da Nogueira - Mtdo. do Leacoque; verbatimElevation: 614; decimalLatitude: 32.7418; decimalLongitude: -16.9177; geodeticDatum: WGS84; **Event:** samplingProtocol: Direct sampling
- **Type status:** Other material. **Occurrence:** sex: 1 male, 1 female; **Location:** locationID: 12; higherGeography: Macaronesia; continent: Europe; waterBody: Atlantic Ocean; islandGroup: Madeira archipelago; island: Madeira; country: Portugal; countryCode: PT; stateProvince: Madeira; county: Santana; locality: Achada do Teixeira; verbatimElevation: 1103; decimalLatitude: 32.7762; decimalLongitude: -16.9022; geodeticDatum: WGS84; **Event:** samplingProtocol: Direct sampling
- **Type status:** Other material. **Occurrence:** sex: 1 male, 2 females; **Location:** locationID: 18; higherGeography: Macaronesia; continent: Europe; waterBody: Atlantic Ocean; islandGroup: Madeira archipelago; island: Madeira; country: Portugal; countryCode: PT; stateProvince: Madeira; county: Porto Moniz; locality: Chão da Ribeira; verbatimElevation: 491; decimalLatitude: 32.7957; decimalLongitude: -17.1117; geodeticDatum: WGS84; **Event:** samplingProtocol: Direct sampling
- **Type status:** Other material. **Occurrence:** sex: 1 male, 3 females; **Location:** locationID: 24; higherGeography: Macaronesia; continent: Europe; waterBody: Atlantic Ocean; islandGroup: Madeira archipelago; island: Madeira; country: Portugal; countryCode: PT; stateProvince: Madeira; county: Porto Moniz; locality: Fanal - Levada dos Cedros; verbatimElevation: 820; decimalLatitude: 32.8259; decimalLongitude: -17.158; geodeticDatum: WGS84; **Event:** samplingProtocol: Direct sampling
- **Type status:** Other material. **Occurrence:** sex: 2 females; **Location:** locationID: 29; higherGeography: Macaronesia; continent: Europe; waterBody: Atlantic Ocean; islandGroup: Madeira archipelago; island: Madeira; country: Portugal; countryCode: PT; stateProvince: Madeira; county: São Vicente; locality: Ginjas; verbatimElevation: 869; decimalLatitude: 32.7758; decimalLongitude: -17.0534; geodeticDatum: WGS84; **Event:** samplingProtocol: Direct sampling
- **Type status:** Other material. **Occurrence:** sex: 1 male, 2 females; **Location:** locationID: 32; higherGeography: Macaronesia; continent: Europe; waterBody: Atlantic Ocean; islandGroup: Madeira archipelago; island: Madeira; country: Portugal; countryCode: PT; stateProvince: Madeira; county: Calheta; locality: Rabaças; verbatimElevation: 993; decimalLatitude: 32.7413; decimalLongitude: -17.0783; geodeticDatum: WGS84; **Event:** samplingProtocol: Direct sampling

##### Ecological interactions

###### Native status

SIE

##### Distribution

Madeira island (Fig. 1b)

##### Notes

So far this species has only been collected in the Laurisilva of Madeira island, where is commonly found under the bark of endemic trees.

#### Micaria pallipes

(Lucas, 1846)

##### Materials

- **Type status:** Other material. **Occurrence:** sex: 9 males, 2 females; **Location:** locationID: 15; higherGeography: Macaronesia; continent: Europe; waterBody: Atlantic Ocean; islandGroup: Madeira archipelago; island: Madeira; country: Portugal; countryCode: PT; stateProvince: Madeira; county: Santana; locality: Pico do Areeiro; verbatimElevation: 1533; decimalLatitude: 32.7231; decimalLongitude: -16.9109; geodeticDatum: WGS84; **Event:** samplingProtocol: Pitfall
- **Type status:** Other material. **Occurrence:** sex: 6 males, 7 females; **Location:** locationID: 16; higherGeography: Macaronesia; continent: Europe; waterBody: Atlantic Ocean; islandGroup: Madeira archipelago; island: Madeira; country: Portugal; countryCode: PT; stateProvince: Madeira; county: Santana; locality: Pico do Areeiro; verbatimElevation: 1594; decimalLatitude: 32.7287; decimalLongitude: -16.9202; geodeticDatum: WGS84; **Event:** samplingProtocol: Pitfall

##### Ecological interactions

###### Native status

introduced

##### Distribution

Madeira island, Canary Islands, Europe to Central Asia

#### Zelotes civicus

(Simon, 1878)

##### Materials

- **Type status:** Other material. **Occurrence:** sex: 54 males, 25 females; **Location:** locationID: 15; higherGeography: Macaronesia; continent: Europe; waterBody: Atlantic Ocean; islandGroup: Madeira archipelago; island: Madeira; country: Portugal; countryCode: PT; stateProvince: Madeira; county: Santana; locality: Pico do Areeiro; verbatimElevation: 1533; decimalLatitude: 32.7231; decimalLongitude: -16.9109; geodeticDatum: WGS84; **Event:** samplingProtocol: Pitfall
- **Type status:** Other material. **Occurrence:** sex: 99 males, 36 females; **Location:** locationID: 16; higherGeography: Macaronesia; continent: Europe; waterBody: Atlantic Ocean; islandGroup: Madeira archipelago; island: Madeira; country: Portugal; countryCode: PT; stateProvince: Madeira; county: Santana; locality: Pico do Areeiro; verbatimElevation: 1594; decimalLatitude: 32.7287; decimalLongitude: -16.9202; geodeticDatum: WGS84; **Event:** samplingProtocol: Pitfall
- **Type status:** Other material. **Occurrence:** sex: 1 male; **Location:** locationID: 32; higherGeography: Macaronesia; continent: Europe; waterBody: Atlantic Ocean; islandGroup: Madeira archipelago; island: Madeira; country: Portugal; countryCode: PT; stateProvince: Madeira; county: Calheta; locality: Rabaças; verbatimElevation: 993; decimalLatitude: 32.7413; decimalLongitude: -17.0783; geodeticDatum: WGS84; **Event:** samplingProtocol: Direct sampling
- **Type status:** Other material. **Occurrence:** sex: 3 males; **Location:** locationID: 32; higherGeography: Macaronesia; continent: Europe; waterBody: Atlantic Ocean; islandGroup: Madeira archipelago; island: Madeira; country: Portugal; countryCode: PT; stateProvince: Madeira; county: Calheta; locality: Rabaças; verbatimElevation: 993; decimalLatitude: 32.7413; decimalLongitude: -17.0783; geodeticDatum: WGS84; **Event:** samplingProtocol: Pitfall

##### Ecological interactions

###### Native status

introduced

##### Distribution

Europe, Madeira archipelago

#### Hahnia insulana

Schenkel, 1938

##### Materials

- **Type status:** Other material. **Occurrence:** sex: 1 female; **Location:** locationID: 6; higherGeography: Macaronesia; continent: Europe; waterBody: Atlantic Ocean; islandGroup: Madeira archipelago; island: Madeira; country: Portugal; countryCode: PT; stateProvince: Madeira; county: Santana; locality: Fajã da Nogueira - Mtdo. do Leacoque; verbatimElevation: 614; decimalLatitude: 32.7418; decimalLongitude: -16.9177; geodeticDatum: WGS84; **Event:** samplingProtocol: Direct sampling
- **Type status:** Other material. **Occurrence:** sex: 2 males; **Location:** locationID: 7; higherGeography: Macaronesia; continent: Europe; waterBody: Atlantic Ocean; islandGroup: Madeira archipelago; island: Madeira; country: Portugal; countryCode: PT; stateProvince: Madeira; county: Santana; locality: Fajã da Nogueira - Tanque; verbatimElevation: 845; decimalLatitude: 32.7425; decimalLongitude: -16.9168; geodeticDatum: WGS84; **Event:** samplingProtocol: Direct sampling
- **Type status:** Other material. **Occurrence:** sex: 1 male; **Location:** locationID: 9; higherGeography: Macaronesia; continent: Europe; waterBody: Atlantic Ocean; islandGroup: Madeira archipelago; island: Madeira; country: Portugal; countryCode: PT; stateProvince: Madeira; county: Santana; locality: Queimadas; verbatimElevation: 841; decimalLatitude: 32.7873; decimalLongitude: -16.9047; geodeticDatum: WGS84; **Event:** samplingProtocol: Direct sampling
- **Type status:** Other material. **Occurrence:** sex: 3 males, 1 female; **Location:** locationID: 12; higherGeography: Macaronesia; continent: Europe; waterBody: Atlantic Ocean; islandGroup: Madeira archipelago; island: Madeira; country: Portugal; countryCode: PT; stateProvince: Madeira; county: Santana; locality: Achada do Teixeira; verbatimElevation: 1103; decimalLatitude: 32.7762; decimalLongitude: -16.9022; geodeticDatum: WGS84; **Event:** samplingProtocol: Direct sampling
- **Type status:** Other material. **Occurrence:** sex: 1 male; **Location:** locationID: 13; higherGeography: Macaronesia; continent: Europe; waterBody: Atlantic Ocean; islandGroup: Madeira archipelago; island: Madeira; country: Portugal; countryCode: PT; stateProvince: Madeira; county: Santana; locality: Ribeiro Frio - Viveiro; verbatimElevation: 906; decimalLatitude: 32.7354; decimalLongitude: -16.8864; geodeticDatum: WGS84; **Event:** samplingProtocol: Direct sampling
- **Type status:** Other material. **Occurrence:** sex: 1 female; **Location:** locationID: 14; higherGeography: Macaronesia; continent: Europe; waterBody: Atlantic Ocean; islandGroup: Madeira archipelago; island: Madeira; country: Portugal; countryCode: PT; stateProvince: Madeira; county: Santana; locality: Ribeiro Frio - Cottages; verbatimElevation: 994; decimalLatitude: 32.7319; decimalLongitude: -16.8861; geodeticDatum: WGS84; **Event:** samplingProtocol: Direct sampling
- **Type status:** Other material. **Occurrence:** sex: 2 males; **Location:** locationID: 18; higherGeography: Macaronesia; continent: Europe; waterBody: Atlantic Ocean; islandGroup: Madeira archipelago; island: Madeira; country: Portugal; countryCode: PT; stateProvince: Madeira; county: Porto Moniz; locality: Chão da Ribeira; verbatimElevation: 491; decimalLatitude: 32.7957; decimalLongitude: -17.1117; geodeticDatum: WGS84; **Event:** samplingProtocol: Direct sampling
- **Type status:** Other material. **Occurrence:** sex: 1 male; **Location:** locationID: 21; higherGeography: Macaronesia; continent: Europe; waterBody: Atlantic Ocean; islandGroup: Madeira archipelago; island: Madeira; country: Portugal; countryCode: PT; stateProvince: Madeira; county: Santana; locality: Ribeiro Bonito - Levada; verbatimElevation: 568; decimalLatitude: 32.8047; decimalLongitude: -16.9346; geodeticDatum: WGS84; **Event:** samplingProtocol: Direct sampling
- **Type status:** Other material. **Occurrence:** sex: 1 male; **Location:** locationID: 33; higherGeography: Macaronesia; continent: Europe; waterBody: Atlantic Ocean; islandGroup: Madeira archipelago; island: Madeira; country: Portugal; countryCode: PT; stateProvince: Madeira; county: Porto Moniz; locality: Rabaçal; verbatimElevation: 930; decimalLatitude: 32.7647; decimalLongitude: -17.1341; geodeticDatum: WGS84; **Event:** samplingProtocol: Direct sampling
- **Type status:** Other material. **Occurrence:** sex: 1 female; **Location:** locationID: 34; higherGeography: Macaronesia; continent: Europe; waterBody: Atlantic Ocean; islandGroup: Madeira archipelago; island: Madeira; country: Portugal; countryCode: PT; stateProvince: Madeira; county: Ponta do Sol; locality: Risco; verbatimElevation: 1048; decimalLatitude: 32.7608; decimalLongitude: -17.1256; geodeticDatum: WGS84; **Event:** samplingProtocol: Direct sampling

##### Ecological interactions

###### Native status

SIE

##### Distribution

Madeira island (Fig. 1c).

##### Notes

This is the first time this species was captured after its description (Schenkel 1938). The previous record only included adult specimens from Caramujo, but this study shows that the species has a wider distribution including many Laurisilva sites in Madeira island.

#### Centromerus variegatus

Denis, 1962

##### Materials

- **Type status:** Other material. **Occurrence:** sex: 1 male, 2 females; **Location:** locationID: 25; higherGeography: Macaronesia; continent: Europe; waterBody: Atlantic Ocean; islandGroup: Madeira archipelago; island: Madeira; country: Portugal; countryCode: PT; stateProvince: Madeira; county: Porto Moniz; locality: Fanal; verbatimElevation: 890; decimalLatitude: 32.8236; decimalLongitude: -17.156; geodeticDatum: WGS84; **Event:** samplingProtocol: Direct sampling

##### Ecological interactions

###### Native status

SIE

##### Distribution

Madeira island (Fig. 1d)

##### Notes

Records of *Centromerus variegatus* are extremely scarce. After its formal description based on five specimens collected in Santo da Serra (Denis 1962), only an additional specimen was later reported from Rabaçal (Wunderlich 1987).

#### Ceratinopsis acripes

(Denis, 1962)

##### Materials

- **Type status:** Other material. **Occurrence:** sex: 1 female; **Location:** locationID: 2; higherGeography: Macaronesia; continent: Europe; waterBody: Atlantic Ocean; islandGroup: Madeira archipelago; island: Madeira; country: Portugal; countryCode: PT; stateProvince: Madeira; county: Machico; locality: Funduras; verbatimElevation: 552; decimalLatitude: 32.754; decimalLongitude: -16.8099; geodeticDatum: WGS84; **Event:** samplingProtocol: Direct sampling
- **Type status:** Other material. **Occurrence:** sex: 1 female; **Location:** locationID: 7; higherGeography: Macaronesia; continent: Europe; waterBody: Atlantic Ocean; islandGroup: Madeira archipelago; island: Madeira; country: Portugal; countryCode: PT; stateProvince: Madeira; county: Santana; locality: Fajã da Nogueira - Tanque; verbatimElevation: 845; decimalLatitude: 32.7425; decimalLongitude: -16.9168; geodeticDatum: WGS84; **Event:** samplingProtocol: Direct sampling
- **Type status:** Other material. **Occurrence:** sex: 1 female; **Location:** locationID: 33; higherGeography: Macaronesia; continent: Europe; waterBody: Atlantic Ocean; islandGroup: Madeira archipelago; island: Madeira; country: Portugal; countryCode: PT; stateProvince: Madeira; county: Porto Moniz; locality: Rabaçal; verbatimElevation: 930; decimalLatitude: 32.7647; decimalLongitude: -17.1341; geodeticDatum: WGS84; **Event:** samplingProtocol: Pitfall

##### Ecological interactions

###### Native status

SIE

##### Distribution

Madeira island (Fig. 2a)

##### Notes

*Ceratinopsis acripes* seems to be restricted to Laurisilva.

#### Ceratinopsis infuscata

(Denis, 1962)

##### Materials

- **Type status:** Other material. **Occurrence:** sex: 1 female; **Location:** locationID: 11; higherGeography: Macaronesia; continent: Europe; waterBody: Atlantic Ocean; islandGroup: Madeira archipelago; island: Madeira; country: Portugal; countryCode: PT; stateProvince: Madeira; county: Santana; locality: Achada do Teixeira; verbatimElevation: 1211; decimalLatitude: 32.7733; decimalLongitude: -16.9081; geodeticDatum: WGS84; **Event:** samplingProtocol: Direct sampling
- **Type status:** Other material. **Occurrence:** sex: 1 female; **Location:** locationID: 12; higherGeography: Macaronesia; continent: Europe; waterBody: Atlantic Ocean; islandGroup: Madeira archipelago; island: Madeira; country: Portugal; countryCode: PT; stateProvince: Madeira; county: Santana; locality: Achada do Teixeira; verbatimElevation: 1103; decimalLatitude: 32.7762; decimalLongitude: -16.9022; geodeticDatum: WGS84; **Event:** samplingProtocol: Direct sampling
- **Type status:** Other material. **Occurrence:** sex: 1 female; **Location:** locationID: 18; higherGeography: Macaronesia; continent: Europe; waterBody: Atlantic Ocean; islandGroup: Madeira archipelago; island: Madeira; country: Portugal; countryCode: PT; stateProvince: Madeira; county: Porto Moniz; locality: Chão da Ribeira; verbatimElevation: 491; decimalLatitude: 32.7957; decimalLongitude: -17.1117; geodeticDatum: WGS84; **Event:** samplingProtocol: Direct sampling

##### Ecological interactions

###### Native status

SIE

##### Distribution

Madeira island (Fig. 2b)

##### Notes

*Ceratinopsis infuscata* seems to be restricted to Laurisilva, with an additional record from Santo da Serra (Denis 1962). This record might indicate a change in the landscape of Santo da Serra, which currently does not have Laurisilva.

#### Ceratinopsis n. sp.

##### Materials

- **Type status:** Other material. **Occurrence:** sex: 1 female; **Location:** locationID: 1; higherGeography: Macaronesia; continent: Europe; waterBody: Atlantic Ocean; islandGroup: Madeira archipelago; island: Madeira; country: Portugal; countryCode: PT; stateProvince: Madeira; county: Machico; locality: Funduras; verbatimElevation: 500; decimalLatitude: 32.7493; decimalLongitude: -16.8114; geodeticDatum: WGS84; **Event:** samplingProtocol: Direct sampling
- **Type status:** Other material. **Occurrence:** sex: 1 male; **Location:** locationID: 6; higherGeography: Macaronesia; continent: Europe; waterBody: Atlantic Ocean; islandGroup: Madeira archipelago; island: Madeira; country: Portugal; countryCode: PT; stateProvince: Madeira; county: Santana; locality: Fajã da Nogueira - Mtdo. do Leacoque; verbatimElevation: 614; decimalLatitude: 32.7418; decimalLongitude: -16.9177; geodeticDatum: WGS84; **Event:** samplingProtocol: Direct sampling
- **Type status:** Other material. **Occurrence:** sex: 1 female; **Location:** locationID: 7; higherGeography: Macaronesia; continent: Europe; waterBody: Atlantic Ocean; islandGroup: Madeira archipelago; island: Madeira; country: Portugal; countryCode: PT; stateProvince: Madeira; county: Santana; locality: Fajã da Nogueira - Tanque; verbatimElevation: 845; decimalLatitude: 32.7425; decimalLongitude: -16.9168; geodeticDatum: WGS84; **Event:** samplingProtocol: Direct sampling
- **Type status:** Other material. **Occurrence:** sex: 1 male, 2 females; **Location:** locationID: 13; higherGeography: Macaronesia; continent: Europe; waterBody: Atlantic Ocean; islandGroup: Madeira archipelago; island: Madeira; country: Portugal; countryCode: PT; stateProvince: Madeira; county: Santana; locality: Ribeiro Frio - Viveiro; verbatimElevation: 906; decimalLatitude: 32.7354; decimalLongitude: -16.8864; geodeticDatum: WGS84; **Event:** samplingProtocol: Direct sampling
- **Type status:** Other material. **Occurrence:** sex: 1 male, 1 female; **Location:** locationID: 14; higherGeography: Macaronesia; continent: Europe; waterBody: Atlantic Ocean; islandGroup: Madeira archipelago; island: Madeira; country: Portugal; countryCode: PT; stateProvince: Madeira; county: Santana; locality: Ribeiro Frio - Cottages; verbatimElevation: 994; decimalLatitude: 32.7319; decimalLongitude: -16.8861; geodeticDatum: WGS84; **Event:** samplingProtocol: Direct sampling
- **Type status:** Other material. **Occurrence:** sex: 1 male, 2 females; **Location:** locationID: 18; higherGeography: Macaronesia; continent: Europe; waterBody: Atlantic Ocean; islandGroup: Madeira archipelago; island: Madeira; country: Portugal; countryCode: PT; stateProvince: Madeira; county: Porto Moniz; locality: Chão da Ribeira; verbatimElevation: 491; decimalLatitude: 32.7957; decimalLongitude: -17.1117; geodeticDatum: WGS84; **Event:** samplingProtocol: Direct sampling
- **Type status:** Other material. **Occurrence:** sex: 1 female; **Location:** locationID: 21; higherGeography: Macaronesia; continent: Europe; waterBody: Atlantic Ocean; islandGroup: Madeira archipelago; island: Madeira; country: Portugal; countryCode: PT; stateProvince: Madeira; county: Santana; locality: Ribeiro Bonito - Levada; verbatimElevation: 568; decimalLatitude: 32.8047; decimalLongitude: -16.9346; geodeticDatum: WGS84; **Event:** samplingProtocol: Direct sampling
- **Type status:** Other material. **Occurrence:** sex: 1 female; **Location:** locationID: 27; higherGeography: Macaronesia; continent: Europe; waterBody: Atlantic Ocean; islandGroup: Madeira archipelago; island: Madeira; country: Portugal; countryCode: PT; stateProvince: Madeira; county: Porto Moniz; locality: Fanal; verbatimElevation: 1023; decimalLatitude: 32.8182; decimalLongitude: -17.1521; geodeticDatum: WGS84; **Event:** samplingProtocol: Direct sampling
- **Type status:** Other material. **Occurrence:** sex: 1 female; **Location:** locationID: 28; higherGeography: Macaronesia; continent: Europe; waterBody: Atlantic Ocean; islandGroup: Madeira archipelago; island: Madeira; country: Portugal; countryCode: PT; stateProvince: Madeira; county: Porto Moniz; locality: Fanal; verbatimElevation: 1134; decimalLatitude: 32.8062; decimalLongitude: -17.1409; geodeticDatum: WGS84; **Event:** samplingProtocol: Direct sampling
- **Type status:** Other material. **Occurrence:** sex: 1 female; **Location:** locationID: 29; higherGeography: Macaronesia; continent: Europe; waterBody: Atlantic Ocean; islandGroup: Madeira archipelago; island: Madeira; country: Portugal; countryCode: PT; stateProvince: Madeira; county: São Vicente; locality: Ginjas; verbatimElevation: 869; decimalLatitude: 32.7758; decimalLongitude: -17.0534; geodeticDatum: WGS84; **Event:** samplingProtocol: Direct sampling
- **Type status:** Other material. **Occurrence:** sex: 1 female; **Location:** locationID: 34; higherGeography: Macaronesia; continent: Europe; waterBody: Atlantic Ocean; islandGroup: Madeira archipelago; island: Madeira; country: Portugal; countryCode: PT; stateProvince: Madeira; county: Ponta do Sol; locality: Risco; verbatimElevation: 1048; decimalLatitude: 32.7608; decimalLongitude: -17.1256; geodeticDatum: WGS84; **Event:** samplingProtocol: Direct sampling

##### Ecological interactions

###### Native status

SIE

##### Distribution

Madeira island (Fig. 2c)

##### Notes

This is a new species to science, to be described in a future work. As the previous species of *Ceratinopsis*, it also seems to be restricted to Laurisilva.

#### Diplostyla concolor

(Wider, 1834)

##### Materials

- **Type status:** Other material. **Occurrence:** sex: 1 female; **Location:** locationID: 3; higherGeography: Macaronesia; continent: Europe; waterBody: Atlantic Ocean; islandGroup: Madeira archipelago; island: Madeira; country: Portugal; countryCode: PT; stateProvince: Madeira; county: Santana; locality: Fajã da Nogueira - Pte. Roquete; verbatimElevation: 1074; decimalLatitude: 32.7391; decimalLongitude: -16.9156; geodeticDatum: WGS84; **Event:** samplingProtocol: Pitfall
- **Type status:** Other material. **Occurrence:** sex: 2 females; **Location:** locationID: 16; higherGeography: Macaronesia; continent: Europe; waterBody: Atlantic Ocean; islandGroup: Madeira archipelago; island: Madeira; country: Portugal; countryCode: PT; stateProvince: Madeira; county: Santana; locality: Pico do Areeiro; verbatimElevation: 1594; decimalLatitude: 32.7287; decimalLongitude: -16.9202; geodeticDatum: WGS84; **Event:** samplingProtocol: Pitfall
- **Type status:** Other material. **Occurrence:** sex: 1 female; **Location:** locationID: 18; higherGeography: Macaronesia; continent: Europe; waterBody: Atlantic Ocean; islandGroup: Madeira archipelago; island: Madeira; country: Portugal; countryCode: PT; stateProvince: Madeira; county: Porto Moniz; locality: Chão da Ribeira; verbatimElevation: 491; decimalLatitude: 32.7957; decimalLongitude: -17.1117; geodeticDatum: WGS84; **Event:** samplingProtocol: Pitfall
- **Type status:** Other material. **Occurrence:** sex: 1 female; **Location:** locationID: 20; higherGeography: Macaronesia; continent: Europe; waterBody: Atlantic Ocean; islandGroup: Madeira archipelago; island: Madeira; country: Portugal; countryCode: PT; stateProvince: Madeira; county: São Vicente; locality: Encumeada; verbatimElevation: 999; decimalLatitude: 32.7558; decimalLongitude: -17.0143; geodeticDatum: WGS84; **Event:** samplingProtocol: Pitfall

##### Ecological interactions

###### Native status

introduced

##### Distribution

Holarctic

#### Entelecara schmitzi

Kulczynski, 1905

##### Materials

- **Type status:** Other material. **Occurrence:** sex: 2 females; **Location:** locationID: 6; higherGeography: Macaronesia; continent: Europe; waterBody: Atlantic Ocean; islandGroup: Madeira archipelago; island: Madeira; country: Portugal; countryCode: PT; stateProvince: Madeira; county: Santana; locality: Fajã da Nogueira - Mtdo. do Leacoque; verbatimElevation: 614; decimalLatitude: 32.7418; decimalLongitude: -16.9177; geodeticDatum: WGS84; **Event:** samplingProtocol: Direct sampling
- **Type status:** Other material. **Occurrence:** sex: 2 females; **Location:** locationID: 7; higherGeography: Macaronesia; continent: Europe; waterBody: Atlantic Ocean; islandGroup: Madeira archipelago; island: Madeira; country: Portugal; countryCode: PT; stateProvince: Madeira; county: Santana; locality: Fajã da Nogueira - Tanque; verbatimElevation: 845; decimalLatitude: 32.7425; decimalLongitude: -16.9168; geodeticDatum: WGS84; **Event:** samplingProtocol: Direct sampling
- **Type status:** Other material. **Occurrence:** sex: 1 male; **Location:** locationID: 8; higherGeography: Macaronesia; continent: Europe; waterBody: Atlantic Ocean; islandGroup: Madeira archipelago; island: Madeira; country: Portugal; countryCode: PT; stateProvince: Madeira; county: Santana; locality: Fajã da Nogueira - Til Gigante; verbatimElevation: 841; decimalLatitude: 32.7457; decimalLongitude: -16.915; geodeticDatum: WGS84; **Event:** samplingProtocol: Direct sampling
- **Type status:** Other material. **Occurrence:** sex: 1 male, 1 female; **Location:** locationID: 11; higherGeography: Macaronesia; continent: Europe; waterBody: Atlantic Ocean; islandGroup: Madeira archipelago; island: Madeira; country: Portugal; countryCode: PT; stateProvince: Madeira; county: Santana; locality: Achada do Teixeira; verbatimElevation: 1211; decimalLatitude: 32.7733; decimalLongitude: -16.9081; geodeticDatum: WGS84; **Event:** samplingProtocol: Direct sampling
- **Type status:** Other material. **Occurrence:** sex: 1 female; **Location:** locationID: 13; higherGeography: Macaronesia; continent: Europe; waterBody: Atlantic Ocean; islandGroup: Madeira archipelago; island: Madeira; country: Portugal; countryCode: PT; stateProvince: Madeira; county: Santana; locality: Ribeiro Frio - Viveiro; verbatimElevation: 906; decimalLatitude: 32.7354; decimalLongitude: -16.8864; geodeticDatum: WGS84; **Event:** samplingProtocol: Direct sampling
- **Type status:** Other material. **Occurrence:** sex: 1 female; **Location:** locationID: 14; higherGeography: Macaronesia; continent: Europe; waterBody: Atlantic Ocean; islandGroup: Madeira archipelago; island: Madeira; country: Portugal; countryCode: PT; stateProvince: Madeira; county: Santana; locality: Ribeiro Frio - Cottages; verbatimElevation: 994; decimalLatitude: 32.7319; decimalLongitude: -16.8861; geodeticDatum: WGS84; **Event:** samplingProtocol: Direct sampling
- **Type status:** Other material. **Occurrence:** sex: 1 female; **Location:** locationID: 17; higherGeography: Macaronesia; continent: Europe; waterBody: Atlantic Ocean; islandGroup: Madeira archipelago; island: Madeira; country: Portugal; countryCode: PT; stateProvince: Madeira; county: Porto Moniz; locality: Chão da Ribeira; verbatimElevation: 519; decimalLatitude: 32.7933; decimalLongitude: -17.1122; geodeticDatum: WGS84; **Event:** samplingProtocol: Direct sampling
- **Type status:** Other material. **Occurrence:** sex: 1 female; **Location:** locationID: 18; higherGeography: Macaronesia; continent: Europe; waterBody: Atlantic Ocean; islandGroup: Madeira archipelago; island: Madeira; country: Portugal; countryCode: PT; stateProvince: Madeira; county: Porto Moniz; locality: Chão da Ribeira; verbatimElevation: 491; decimalLatitude: 32.7957; decimalLongitude: -17.1117; geodeticDatum: WGS84; **Event:** samplingProtocol: Direct sampling
- **Type status:** Other material. **Occurrence:** sex: 8 females; **Location:** locationID: 24; higherGeography: Macaronesia; continent: Europe; waterBody: Atlantic Ocean; islandGroup: Madeira archipelago; island: Madeira; country: Portugal; countryCode: PT; stateProvince: Madeira; county: Porto Moniz; locality: Fanal - Levada dos Cedros; verbatimElevation: 820; decimalLatitude: 32.8259; decimalLongitude: -17.158; geodeticDatum: WGS84; **Event:** samplingProtocol: Direct sampling
- **Type status:** Other material. **Occurrence:** sex: 2 females; **Location:** locationID: 25; higherGeography: Macaronesia; continent: Europe; waterBody: Atlantic Ocean; islandGroup: Madeira archipelago; island: Madeira; country: Portugal; countryCode: PT; stateProvince: Madeira; county: Porto Moniz; locality: Fanal; verbatimElevation: 890; decimalLatitude: 32.8236; decimalLongitude: -17.156; geodeticDatum: WGS84; **Event:** samplingProtocol: Direct sampling
- **Type status:** Other material. **Occurrence:** sex: 1 male, 2 females; **Location:** locationID: 26; higherGeography: Macaronesia; continent: Europe; waterBody: Atlantic Ocean; islandGroup: Madeira archipelago; island: Madeira; country: Portugal; countryCode: PT; stateProvince: Madeira; county: Porto Moniz; locality: Fanal; verbatimElevation: 889; decimalLatitude: 32.8226; decimalLongitude: -17.1539; geodeticDatum: WGS84; **Event:** samplingProtocol: Direct sampling
- **Type status:** Other material. **Occurrence:** sex: 1 male; **Location:** locationID: 27; higherGeography: Macaronesia; continent: Europe; waterBody: Atlantic Ocean; islandGroup: Madeira archipelago; island: Madeira; country: Portugal; countryCode: PT; stateProvince: Madeira; county: Porto Moniz; locality: Fanal; verbatimElevation: 1023; decimalLatitude: 32.8182; decimalLongitude: -17.1521; geodeticDatum: WGS84; **Event:** samplingProtocol: Direct sampling
- **Type status:** Other material. **Occurrence:** sex: 1 female; **Location:** locationID: 29; higherGeography: Macaronesia; continent: Europe; waterBody: Atlantic Ocean; islandGroup: Madeira archipelago; island: Madeira; country: Portugal; countryCode: PT; stateProvince: Madeira; county: São Vicente; locality: Ginjas; verbatimElevation: 869; decimalLatitude: 32.7758; decimalLongitude: -17.0534; geodeticDatum: WGS84; **Event:** samplingProtocol: Pitfall
- **Type status:** Other material. **Occurrence:** sex: 1 female; **Location:** locationID: 34; higherGeography: Macaronesia; continent: Europe; waterBody: Atlantic Ocean; islandGroup: Madeira archipelago; island: Madeira; country: Portugal; countryCode: PT; stateProvince: Madeira; county: Ponta do Sol; locality: Risco; verbatimElevation: 1048; decimalLatitude: 32.7608; decimalLongitude: -17.1256; geodeticDatum: WGS84; **Event:** samplingProtocol: Direct sampling

##### Ecological interactions

###### Native status

native

##### Distribution

Madeira island, Azores, France

##### Notes

This species seems to be widespread in Madeira Laurisilva. In the Azores occurs at low altitude, being very common in the canopies of orchards and exotic trees.

#### Frontinellina dearmata

(Kulczynski, 1899)

##### Materials

- **Type status:** Other material. **Occurrence:** sex: 1 male; **Location:** locationID: 18; higherGeography: Macaronesia; continent: Europe; waterBody: Atlantic Ocean; islandGroup: Madeira archipelago; island: Madeira; country: Portugal; countryCode: PT; stateProvince: Madeira; county: Porto Moniz; locality: Chão da Ribeira; verbatimElevation: 491; decimalLatitude: 32.7957; decimalLongitude: -17.1117; geodeticDatum: WGS84; **Event:** samplingProtocol: Direct sampling
- **Type status:** Other material. **Occurrence:** sex: 2 males; **Location:** locationID: 25; higherGeography: Macaronesia; continent: Europe; waterBody: Atlantic Ocean; islandGroup: Madeira archipelago; island: Madeira; country: Portugal; countryCode: PT; stateProvince: Madeira; county: Porto Moniz; locality: Fanal; verbatimElevation: 890; decimalLatitude: 32.8236; decimalLongitude: -17.156; geodeticDatum: WGS84; **Event:** samplingProtocol: Direct sampling

##### Ecological interactions

###### Native status

SIE

##### Distribution

Madeira island (Fig. 2d)

##### Notes

This is the first record of this endemic species since its original description, which is based on females only and fails to include the precise location of the type material. The specimens collected in this study will be used in a future work to describe the male of this endemic species.

#### Frontiphantes fulgurenotatus

(Schenkel, 1938)

##### Materials

- **Type status:** Other material. **Occurrence:** sex: 2 males; **Location:** locationID: 6; higherGeography: Macaronesia; continent: Europe; waterBody: Atlantic Ocean; islandGroup: Madeira archipelago; island: Madeira; country: Portugal; countryCode: PT; stateProvince: Madeira; county: Santana; locality: Fajã da Nogueira - Mtdo. do Leacoque; verbatimElevation: 614; decimalLatitude: 32.7418; decimalLongitude: -16.9177; geodeticDatum: WGS84; **Event:** samplingProtocol: Direct sampling
- **Type status:** Other material. **Occurrence:** sex: 1 male; **Location:** locationID: 11; higherGeography: Macaronesia; continent: Europe; waterBody: Atlantic Ocean; islandGroup: Madeira archipelago; island: Madeira; country: Portugal; countryCode: PT; stateProvince: Madeira; county: Santana; locality: Achada do Teixeira; verbatimElevation: 1211; decimalLatitude: 32.7733; decimalLongitude: -16.9081; geodeticDatum: WGS84; **Event:** samplingProtocol: Direct sampling
- **Type status:** Other material. **Occurrence:** sex: 1 female; **Location:** locationID: 12; higherGeography: Macaronesia; continent: Europe; waterBody: Atlantic Ocean; islandGroup: Madeira archipelago; island: Madeira; country: Portugal; countryCode: PT; stateProvince: Madeira; county: Santana; locality: Achada do Teixeira; verbatimElevation: 1103; decimalLatitude: 32.7762; decimalLongitude: -16.9022; geodeticDatum: WGS84; **Event:** samplingProtocol: Direct sampling
- **Type status:** Other material. **Occurrence:** sex: 1 male; **Location:** locationID: 34; higherGeography: Macaronesia; continent: Europe; waterBody: Atlantic Ocean; islandGroup: Madeira archipelago; island: Madeira; country: Portugal; countryCode: PT; stateProvince: Madeira; county: Ponta do Sol; locality: Risco; verbatimElevation: 1048; decimalLatitude: 32.7608; decimalLongitude: -17.1256; geodeticDatum: WGS84; **Event:** samplingProtocol: Direct sampling

##### Ecological interactions

###### Native status

SIE

##### Distribution

Madeira island (Fig. 3a)

##### Notes

This endemic species seems to be restricted to Laurisilva. The two records outside Laurisilva (Denis 1962) are most likely artifacts due to imprecise location of sampling localities and/or reductions in Laurisilva cover during the last decades.

#### Lepthyphantes impudicus

Kulczynski, 1909

##### Materials

- **Type status:** Other material. **Occurrence:** sex: 1 female; **Location:** locationID: 12; higherGeography: Macaronesia; continent: Europe; waterBody: Atlantic Ocean; islandGroup: Madeira archipelago; island: Madeira; country: Portugal; countryCode: PT; stateProvince: Madeira; county: Santana; locality: Achada do Teixeira; verbatimElevation: 1103; decimalLatitude: 32.7762; decimalLongitude: -16.9022; geodeticDatum: WGS84; **Event:** samplingProtocol: Pitfall
- **Type status:** Other material. **Occurrence:** sex: 1 female; **Location:** locationID: 36; higherGeography: Macaronesia; continent: Europe; waterBody: Atlantic Ocean; islandGroup: Madeira archipelago; island: Madeira; country: Portugal; countryCode: PT; stateProvince: Madeira; county: Calheta; locality: Galhano; verbatimElevation: 975; decimalLatitude: 32.7971; decimalLongitude: -17.1729; geodeticDatum: WGS84; **Event:** samplingProtocol: Pitfall

##### Ecological interactions

###### Native status

SIE

##### Distribution

Madeira island (Fig. 3b)

##### Notes

Distribution data on *Lepthyphantes impudicus* is scarce consisting of only 4 records. This species seems to be associated with Laurisilva and the lack of congruence between historical data (Denis 1962) and the current distribution of Laurisilva may be explained by the lack of precision in the identification of sampling sites and/or by the decrease of Laurisilva cover in the last decades.

#### Lepthyphantes mauli

Wunderlich, 1992

##### Materials

- **Type status:** Other material. **Occurrence:** sex: 1 male; **Location:** locationID: 33; higherGeography: Macaronesia; continent: Europe; waterBody: Atlantic Ocean; islandGroup: Madeira archipelago; island: Madeira; country: Portugal; countryCode: PT; stateProvince: Madeira; county: Porto Moniz; locality: Rabaçal; verbatimElevation: 930; decimalLatitude: 32.7647; decimalLongitude: -17.1341; geodeticDatum: WGS84; **Event:** samplingProtocol: Pitfall

##### Ecological interactions

###### Native status

SIE

##### Distribution

Madeira island (Fig. 3c)

##### Notes

This is solely the second record of this endemic species, thus enlarging its known distribution.

#### Meioneta fuscipalpa

(C. L. Koch, 1836)

##### Materials

- **Type status:** Other material. **Occurrence:** sex: 2 males; **Location:** locationID: 32; higherGeography: Macaronesia; continent: Europe; waterBody: Atlantic Ocean; islandGroup: Madeira archipelago; island: Madeira; country: Portugal; countryCode: PT; stateProvince: Madeira; county: Calheta; locality: Rabaças; verbatimElevation: 993; decimalLatitude: 32.7413; decimalLongitude: -17.0783; geodeticDatum: WGS84; **Event:** samplingProtocol: Pitfall

##### Ecological interactions

###### Native status

introduced

##### Distribution

Palearctic

#### Microctenonyx subitaneus

(O. Pickard-Cambridge, 1875)

##### Materials

- **Type status:** Other material. **Occurrence:** sex: 1 female; **Location:** locationID: 16; higherGeography: Macaronesia; continent: Europe; waterBody: Atlantic Ocean; islandGroup: Madeira archipelago; island: Madeira; country: Portugal; countryCode: PT; stateProvince: Madeira; county: Santana; locality: Pico do Areeiro; verbatimElevation: 1594; decimalLatitude: 32.7287; decimalLongitude: -16.9202; geodeticDatum: WGS84; **Event:** samplingProtocol: Pitfall

##### Ecological interactions

###### Native status

introduced

##### Distribution

Holarctic

#### Microlinyphia johnsoni

(Blackwall, 1859)

##### Materials

- **Type status:** Other material. **Occurrence:** sex: 1 female; **Location:** locationID: 8; higherGeography: Macaronesia; continent: Europe; waterBody: Atlantic Ocean; islandGroup: Madeira archipelago; island: Madeira; country: Portugal; countryCode: PT; stateProvince: Madeira; county: Santana; locality: Fajã da Nogueira - Til Gigante; verbatimElevation: 841; decimalLatitude: 32.7457; decimalLongitude: -16.915; geodeticDatum: WGS84; **Event:** samplingProtocol: Pitfall

##### Ecological interactions

###### Native status

macaronesian endemic

##### Distribution

Madeira archipelago, Azores, Canary Islands

##### Notes

A Macaronesian endemic also recorded from Porto Santo island and very common in native forests of the Azores.

#### Palliduphantes schmitzi

(Kulczynski, 1899)

##### Materials

- **Type status:** Other material. **Occurrence:** sex: 2 females; **Location:** locationID: 1; higherGeography: Macaronesia; continent: Europe; waterBody: Atlantic Ocean; islandGroup: Madeira archipelago; island: Madeira; country: Portugal; countryCode: PT; stateProvince: Madeira; county: Machico; locality: Funduras; verbatimElevation: 500; decimalLatitude: 32.7493; decimalLongitude: -16.8114; geodeticDatum: WGS84; **Event:** samplingProtocol: Pitfall
- **Type status:** Other material. **Occurrence:** sex: 2 males, 8 females; **Location:** locationID: 2; higherGeography: Macaronesia; continent: Europe; waterBody: Atlantic Ocean; islandGroup: Madeira archipelago; island: Madeira; country: Portugal; countryCode: PT; stateProvince: Madeira; county: Machico; locality: Funduras; verbatimElevation: 552; decimalLatitude: 32.754; decimalLongitude: -16.8099; geodeticDatum: WGS84; **Event:** samplingProtocol: Pitfall
- **Type status:** Other material. **Occurrence:** sex: 1 female; **Location:** locationID: 3; higherGeography: Macaronesia; continent: Europe; waterBody: Atlantic Ocean; islandGroup: Madeira archipelago; island: Madeira; country: Portugal; countryCode: PT; stateProvince: Madeira; county: Santana; locality: Fajã da Nogueira - Pte. Roquete; verbatimElevation: 1074; decimalLatitude: 32.7391; decimalLongitude: -16.9156; geodeticDatum: WGS84; **Event:** samplingProtocol: Pitfall
- **Type status:** Other material. **Occurrence:** sex: 4 females; **Location:** locationID: 4; higherGeography: Macaronesia; continent: Europe; waterBody: Atlantic Ocean; islandGroup: Madeira archipelago; island: Madeira; country: Portugal; countryCode: PT; stateProvince: Madeira; county: Santana; locality: Fajã da Nogueira - Mtdo. do Leacoque; verbatimElevation: 630; decimalLatitude: 32.7415; decimalLongitude: -16.9161; geodeticDatum: WGS84; **Event:** samplingProtocol: Direct sampling
- **Type status:** Other material. **Occurrence:** sex: 1 female; **Location:** locationID: 4; higherGeography: Macaronesia; continent: Europe; waterBody: Atlantic Ocean; islandGroup: Madeira archipelago; island: Madeira; country: Portugal; countryCode: PT; stateProvince: Madeira; county: Santana; locality: Fajã da Nogueira - Mtdo. do Leacoque; verbatimElevation: 630; decimalLatitude: 32.7415; decimalLongitude: -16.9161; geodeticDatum: WGS84; **Event:** samplingProtocol: Pitfall
- **Type status:** Other material. **Occurrence:** sex: 1 male, 2 females; **Location:** locationID: 5; higherGeography: Macaronesia; continent: Europe; waterBody: Atlantic Ocean; islandGroup: Madeira archipelago; island: Madeira; country: Portugal; countryCode: PT; stateProvince: Madeira; county: Santana; locality: Fajã da Nogueira - Casa do Levadeiro; verbatimElevation: 989; decimalLatitude: 32.7406; decimalLongitude: -16.9136; geodeticDatum: WGS84; **Event:** samplingProtocol: Pitfall
- **Type status:** Other material. **Occurrence:** sex: 1 female; **Location:** locationID: 6; higherGeography: Macaronesia; continent: Europe; waterBody: Atlantic Ocean; islandGroup: Madeira archipelago; island: Madeira; country: Portugal; countryCode: PT; stateProvince: Madeira; county: Santana; locality: Fajã da Nogueira - Mtdo. do Leacoque; verbatimElevation: 614; decimalLatitude: 32.7418; decimalLongitude: -16.9177; geodeticDatum: WGS84; **Event:** samplingProtocol: Direct sampling
- **Type status:** Other material. **Occurrence:** sex: 1 male, 1 female; **Location:** locationID: 7; higherGeography: Macaronesia; continent: Europe; waterBody: Atlantic Ocean; islandGroup: Madeira archipelago; island: Madeira; country: Portugal; countryCode: PT; stateProvince: Madeira; county: Santana; locality: Fajã da Nogueira - Tanque; verbatimElevation: 845; decimalLatitude: 32.7425; decimalLongitude: -16.9168; geodeticDatum: WGS84; **Event:** samplingProtocol: Pitfall
- **Type status:** Other material. **Occurrence:** sex: 1 male, 1 female; **Location:** locationID: 9; higherGeography: Macaronesia; continent: Europe; waterBody: Atlantic Ocean; islandGroup: Madeira archipelago; island: Madeira; country: Portugal; countryCode: PT; stateProvince: Madeira; county: Santana; locality: Queimadas; verbatimElevation: 841; decimalLatitude: 32.7873; decimalLongitude: -16.9047; geodeticDatum: WGS84; **Event:** samplingProtocol: Pitfall
- **Type status:** Other material. **Occurrence:** sex: 1 female; **Location:** locationID: 10; higherGeography: Macaronesia; continent: Europe; waterBody: Atlantic Ocean; islandGroup: Madeira archipelago; island: Madeira; country: Portugal; countryCode: PT; stateProvince: Madeira; county: Santana; locality: Pico das Pedras; verbatimElevation: 883; decimalLatitude: 32.7841; decimalLongitude: -16.9055; geodeticDatum: WGS84; **Event:** samplingProtocol: Direct sampling
- **Type status:** Other material. **Occurrence:** sex: 1 male, 2 females; **Location:** locationID: 10; higherGeography: Macaronesia; continent: Europe; waterBody: Atlantic Ocean; islandGroup: Madeira archipelago; island: Madeira; country: Portugal; countryCode: PT; stateProvince: Madeira; county: Santana; locality: Pico das Pedras; verbatimElevation: 883; decimalLatitude: 32.7841; decimalLongitude: -16.9055; geodeticDatum: WGS84; **Event:** samplingProtocol: Pitfall
- **Type status:** Other material. **Occurrence:** sex: 1 female; **Location:** locationID: 11; higherGeography: Macaronesia; continent: Europe; waterBody: Atlantic Ocean; islandGroup: Madeira archipelago; island: Madeira; country: Portugal; countryCode: PT; stateProvince: Madeira; county: Santana; locality: Achada do Teixeira; verbatimElevation: 1211; decimalLatitude: 32.7733; decimalLongitude: -16.9081; geodeticDatum: WGS84; **Event:** samplingProtocol: Direct sampling
- **Type status:** Other material. **Occurrence:** sex: 4 males, 2 females; **Location:** locationID: 11; higherGeography: Macaronesia; continent: Europe; waterBody: Atlantic Ocean; islandGroup: Madeira archipelago; island: Madeira; country: Portugal; countryCode: PT; stateProvince: Madeira; county: Santana; locality: Achada do Teixeira; verbatimElevation: 1211; decimalLatitude: 32.7733; decimalLongitude: -16.9081; geodeticDatum: WGS84; **Event:** samplingProtocol: Pitfall
- **Type status:** Other material. **Occurrence:** sex: 2 males, 3 females; **Location:** locationID: 12; higherGeography: Macaronesia; continent: Europe; waterBody: Atlantic Ocean; islandGroup: Madeira archipelago; island: Madeira; country: Portugal; countryCode: PT; stateProvince: Madeira; county: Santana; locality: Achada do Teixeira; verbatimElevation: 1103; decimalLatitude: 32.7762; decimalLongitude: -16.9022; geodeticDatum: WGS84; **Event:** samplingProtocol: Pitfall
- **Type status:** Other material. **Occurrence:** sex: 1 male, 1 female; **Location:** locationID: 13; higherGeography: Macaronesia; continent: Europe; waterBody: Atlantic Ocean; islandGroup: Madeira archipelago; island: Madeira; country: Portugal; countryCode: PT; stateProvince: Madeira; county: Santana; locality: Ribeiro Frio - Viveiro; verbatimElevation: 906; decimalLatitude: 32.7354; decimalLongitude: -16.8864; geodeticDatum: WGS84; **Event:** samplingProtocol: Pitfall
- **Type status:** Other material. **Occurrence:** sex: 1 male; **Location:** locationID: 15; higherGeography: Macaronesia; continent: Europe; waterBody: Atlantic Ocean; islandGroup: Madeira archipelago; island: Madeira; country: Portugal; countryCode: PT; stateProvince: Madeira; county: Santana; locality: Pico do Areeiro; verbatimElevation: 1533; decimalLatitude: 32.7231; decimalLongitude: -16.9109; geodeticDatum: WGS84; **Event:** samplingProtocol: Pitfall
- **Type status:** Other material. **Occurrence:** sex: 2 males; **Location:** locationID: 17; higherGeography: Macaronesia; continent: Europe; waterBody: Atlantic Ocean; islandGroup: Madeira archipelago; island: Madeira; country: Portugal; countryCode: PT; stateProvince: Madeira; county: Porto Moniz; locality: Chão da Ribeira; verbatimElevation: 519; decimalLatitude: 32.7933; decimalLongitude: -17.1122; geodeticDatum: WGS84; **Event:** samplingProtocol: Pitfall
- **Type status:** Other material. **Occurrence:** sex: 1 male, 2 females; **Location:** locationID: 18; higherGeography: Macaronesia; continent: Europe; waterBody: Atlantic Ocean; islandGroup: Madeira archipelago; island: Madeira; country: Portugal; countryCode: PT; stateProvince: Madeira; county: Porto Moniz; locality: Chão da Ribeira; verbatimElevation: 491; decimalLatitude: 32.7957; decimalLongitude: -17.1117; geodeticDatum: WGS84; **Event:** samplingProtocol: Pitfall
- **Type status:** Other material. **Occurrence:** sex: 3 females; **Location:** locationID: 19; higherGeography: Macaronesia; continent: Europe; waterBody: Atlantic Ocean; islandGroup: Madeira archipelago; island: Madeira; country: Portugal; countryCode: PT; stateProvince: Madeira; county: São Vicente; locality: Chão dos Louros; verbatimElevation: 748; decimalLatitude: 32.7636; decimalLongitude: -17.019; geodeticDatum: WGS84; **Event:** samplingProtocol: Pitfall
- **Type status:** Other material. **Occurrence:** sex: 2 males; **Location:** locationID: 20; higherGeography: Macaronesia; continent: Europe; waterBody: Atlantic Ocean; islandGroup: Madeira archipelago; island: Madeira; country: Portugal; countryCode: PT; stateProvince: Madeira; county: São Vicente; locality: Encumeada; verbatimElevation: 999; decimalLatitude: 32.7558; decimalLongitude: -17.0143; geodeticDatum: WGS84; **Event:** samplingProtocol: Pitfall
- **Type status:** Other material. **Occurrence:** sex: 1 male; **Location:** locationID: 21; higherGeography: Macaronesia; continent: Europe; waterBody: Atlantic Ocean; islandGroup: Madeira archipelago; island: Madeira; country: Portugal; countryCode: PT; stateProvince: Madeira; county: Santana; locality: Ribeiro Bonito - Levada; verbatimElevation: 568; decimalLatitude: 32.8047; decimalLongitude: -16.9346; geodeticDatum: WGS84; **Event:** samplingProtocol: Direct sampling
- **Type status:** Other material. **Occurrence:** sex: 1 female; **Location:** locationID: 21; higherGeography: Macaronesia; continent: Europe; waterBody: Atlantic Ocean; islandGroup: Madeira archipelago; island: Madeira; country: Portugal; countryCode: PT; stateProvince: Madeira; county: Santana; locality: Ribeiro Bonito - Levada; verbatimElevation: 568; decimalLatitude: 32.8047; decimalLongitude: -16.9346; geodeticDatum: WGS84; **Event:** samplingProtocol: Pitfall
- **Type status:** Other material. **Occurrence:** sex: 1 female; **Location:** locationID: 22; higherGeography: Macaronesia; continent: Europe; waterBody: Atlantic Ocean; islandGroup: Madeira archipelago; island: Madeira; country: Portugal; countryCode: PT; stateProvince: Madeira; county: Santana; locality: Ribeiro Bonito - Ribeiro; verbatimElevation: 560; decimalLatitude: 32.7985; decimalLongitude: -16.936; geodeticDatum: WGS84; **Event:** samplingProtocol: Direct sampling
- **Type status:** Other material. **Occurrence:** sex: 1 male, 1 female; **Location:** locationID: 28; higherGeography: Macaronesia; continent: Europe; waterBody: Atlantic Ocean; islandGroup: Madeira archipelago; island: Madeira; country: Portugal; countryCode: PT; stateProvince: Madeira; county: Porto Moniz; locality: Fanal; verbatimElevation: 1134; decimalLatitude: 32.8062; decimalLongitude: -17.1409; geodeticDatum: WGS84; **Event:** samplingProtocol: Pitfall
- **Type status:** Other material. **Occurrence:** sex: 1 male; **Location:** locationID: 29; higherGeography: Macaronesia; continent: Europe; waterBody: Atlantic Ocean; islandGroup: Madeira archipelago; island: Madeira; country: Portugal; countryCode: PT; stateProvince: Madeira; county: São Vicente; locality: Ginjas; verbatimElevation: 869; decimalLatitude: 32.7758; decimalLongitude: -17.0534; geodeticDatum: WGS84; **Event:** samplingProtocol: Pitfall
- **Type status:** Other material. **Occurrence:** sex: 1 male, 1 female; **Location:** locationID: 30; higherGeography: Macaronesia; continent: Europe; waterBody: Atlantic Ocean; islandGroup: Madeira archipelago; island: Madeira; country: Portugal; countryCode: PT; stateProvince: Madeira; county: São Vicente; locality: Caramujo; verbatimElevation: 981; decimalLatitude: 32.7722; decimalLongitude: -17.0529; geodeticDatum: WGS84; **Event:** samplingProtocol: Pitfall
- **Type status:** Other material. **Occurrence:** sex: 1 male; **Location:** locationID: 31; higherGeography: Macaronesia; continent: Europe; waterBody: Atlantic Ocean; islandGroup: Madeira archipelago; island: Madeira; country: Portugal; countryCode: PT; stateProvince: Madeira; county: São Vicente; locality: Caramujo; verbatimElevation: 1001; decimalLatitude: 32.7746; decimalLongitude: -17.0559; geodeticDatum: WGS84; **Event:** samplingProtocol: Pitfall
- **Type status:** Other material. **Occurrence:** sex: 2 males; **Location:** locationID: 36; higherGeography: Macaronesia; continent: Europe; waterBody: Atlantic Ocean; islandGroup: Madeira archipelago; island: Madeira; country: Portugal; countryCode: PT; stateProvince: Madeira; county: Calheta; locality: Galhano; verbatimElevation: 975; decimalLatitude: 32.7971; decimalLongitude: -17.1729; geodeticDatum: WGS84; **Event:** samplingProtocol: Pitfall

##### Ecological interactions

###### Native status

macaronesian endemic

##### Distribution

Madeira island, Azores

#### Poeciloneta variegata

(Blackwall, 1841)

##### Materials

- **Type status:** Other material. **Occurrence:** sex: 1 male; **Location:** locationID: 24; higherGeography: Macaronesia; continent: Europe; waterBody: Atlantic Ocean; islandGroup: Madeira archipelago; island: Madeira; country: Portugal; countryCode: PT; stateProvince: Madeira; county: Porto Moniz; locality: Fanal - Levada dos Cedros; verbatimElevation: 820; decimalLatitude: 32.8259; decimalLongitude: -17.158; geodeticDatum: WGS84; **Event:** samplingProtocol: Direct sampling
- **Type status:** Other material. **Occurrence:** sex: 1 male, 1 female; **Location:** locationID: 28; higherGeography: Macaronesia; continent: Europe; waterBody: Atlantic Ocean; islandGroup: Madeira archipelago; island: Madeira; country: Portugal; countryCode: PT; stateProvince: Madeira; county: Porto Moniz; locality: Fanal; verbatimElevation: 1134; decimalLatitude: 32.8062; decimalLongitude: -17.1409; geodeticDatum: WGS84; **Event:** samplingProtocol: Direct sampling

##### Ecological interactions

###### Native status

introduced

##### Distribution

Holarctic

##### Notes

First record for Madeira island.

#### Tenuiphantes tenuis

(Blackwall, 1852)

##### Materials

- **Type status:** Other material. **Occurrence:** sex: 4 males, 5 females; **Location:** locationID: 1; higherGeography: Macaronesia; continent: Europe; waterBody: Atlantic Ocean; islandGroup: Madeira archipelago; island: Madeira; country: Portugal; countryCode: PT; stateProvince: Madeira; county: Machico; locality: Funduras; verbatimElevation: 500; decimalLatitude: 32.7493; decimalLongitude: -16.8114; geodeticDatum: WGS84; **Event:** samplingProtocol: Direct sampling
- **Type status:** Other material. **Occurrence:** sex: 1 male, 3 females; **Location:** locationID: 1; higherGeography: Macaronesia; continent: Europe; waterBody: Atlantic Ocean; islandGroup: Madeira archipelago; island: Madeira; country: Portugal; countryCode: PT; stateProvince: Madeira; county: Machico; locality: Funduras; verbatimElevation: 500; decimalLatitude: 32.7493; decimalLongitude: -16.8114; geodeticDatum: WGS84; **Event:** samplingProtocol: Pitfall
- **Type status:** Other material. **Occurrence:** sex: 19 males, 22 females; **Location:** locationID: 2; higherGeography: Macaronesia; continent: Europe; waterBody: Atlantic Ocean; islandGroup: Madeira archipelago; island: Madeira; country: Portugal; countryCode: PT; stateProvince: Madeira; county: Machico; locality: Funduras; verbatimElevation: 552; decimalLatitude: 32.754; decimalLongitude: -16.8099; geodeticDatum: WGS84; **Event:** samplingProtocol: Direct sampling
- **Type status:** Other material. **Occurrence:** sex: 5 males, 7 females; **Location:** locationID: 2; higherGeography: Macaronesia; continent: Europe; waterBody: Atlantic Ocean; islandGroup: Madeira archipelago; island: Madeira; country: Portugal; countryCode: PT; stateProvince: Madeira; county: Machico; locality: Funduras; verbatimElevation: 552; decimalLatitude: 32.754; decimalLongitude: -16.8099; geodeticDatum: WGS84; **Event:** samplingProtocol: Pitfall
- **Type status:** Other material. **Occurrence:** sex: 1 female; **Location:** locationID: 3; higherGeography: Macaronesia; continent: Europe; waterBody: Atlantic Ocean; islandGroup: Madeira archipelago; island: Madeira; country: Portugal; countryCode: PT; stateProvince: Madeira; county: Santana; locality: Fajã da Nogueira - Pte. Roquete; verbatimElevation: 1074; decimalLatitude: 32.7391; decimalLongitude: -16.9156; geodeticDatum: WGS84; **Event:** samplingProtocol: Pitfall
- **Type status:** Other material. **Occurrence:** sex: 5 males, 6 females; **Location:** locationID: 4; higherGeography: Macaronesia; continent: Europe; waterBody: Atlantic Ocean; islandGroup: Madeira archipelago; island: Madeira; country: Portugal; countryCode: PT; stateProvince: Madeira; county: Santana; locality: Fajã da Nogueira - Mtdo. do Leacoque; verbatimElevation: 630; decimalLatitude: 32.7415; decimalLongitude: -16.9161; geodeticDatum: WGS84; **Event:** samplingProtocol: Direct sampling
- **Type status:** Other material. **Occurrence:** sex: 1 male, 4 females; **Location:** locationID: 4; higherGeography: Macaronesia; continent: Europe; waterBody: Atlantic Ocean; islandGroup: Madeira archipelago; island: Madeira; country: Portugal; countryCode: PT; stateProvince: Madeira; county: Santana; locality: Fajã da Nogueira - Mtdo. do Leacoque; verbatimElevation: 630; decimalLatitude: 32.7415; decimalLongitude: -16.9161; geodeticDatum: WGS84; **Event:** samplingProtocol: Pitfall
- **Type status:** Other material. **Occurrence:** sex: 1 female; **Location:** locationID: 5; higherGeography: Macaronesia; continent: Europe; waterBody: Atlantic Ocean; islandGroup: Madeira archipelago; island: Madeira; country: Portugal; countryCode: PT; stateProvince: Madeira; county: Santana; locality: Fajã da Nogueira - Casa do Levadeiro; verbatimElevation: 989; decimalLatitude: 32.7406; decimalLongitude: -16.9136; geodeticDatum: WGS84; **Event:** samplingProtocol: Pitfall
- **Type status:** Other material. **Occurrence:** sex: 8 males, 16 females; **Location:** locationID: 6; higherGeography: Macaronesia; continent: Europe; waterBody: Atlantic Ocean; islandGroup: Madeira archipelago; island: Madeira; country: Portugal; countryCode: PT; stateProvince: Madeira; county: Santana; locality: Fajã da Nogueira - Mtdo. do Leacoque; verbatimElevation: 614; decimalLatitude: 32.7418; decimalLongitude: -16.9177; geodeticDatum: WGS84; **Event:** samplingProtocol: Direct sampling
- **Type status:** Other material. **Occurrence:** sex: 2 males, 4 females; **Location:** locationID: 6; higherGeography: Macaronesia; continent: Europe; waterBody: Atlantic Ocean; islandGroup: Madeira archipelago; island: Madeira; country: Portugal; countryCode: PT; stateProvince: Madeira; county: Santana; locality: Fajã da Nogueira - Mtdo. do Leacoque; verbatimElevation: 614; decimalLatitude: 32.7418; decimalLongitude: -16.9177; geodeticDatum: WGS84; **Event:** samplingProtocol: Pitfall
- **Type status:** Other material. **Occurrence:** sex: 3 males, 7 females; **Location:** locationID: 7; higherGeography: Macaronesia; continent: Europe; waterBody: Atlantic Ocean; islandGroup: Madeira archipelago; island: Madeira; country: Portugal; countryCode: PT; stateProvince: Madeira; county: Santana; locality: Fajã da Nogueira - Tanque; verbatimElevation: 845; decimalLatitude: 32.7425; decimalLongitude: -16.9168; geodeticDatum: WGS84; **Event:** samplingProtocol: Direct sampling
- **Type status:** Other material. **Occurrence:** sex: 4 males, 1 female; **Location:** locationID: 7; higherGeography: Macaronesia; continent: Europe; waterBody: Atlantic Ocean; islandGroup: Madeira archipelago; island: Madeira; country: Portugal; countryCode: PT; stateProvince: Madeira; county: Santana; locality: Fajã da Nogueira - Tanque; verbatimElevation: 845; decimalLatitude: 32.7425; decimalLongitude: -16.9168; geodeticDatum: WGS84; **Event:** samplingProtocol: Pitfall
- **Type status:** Other material. **Occurrence:** sex: 4 males, 9 females; **Location:** locationID: 8; higherGeography: Macaronesia; continent: Europe; waterBody: Atlantic Ocean; islandGroup: Madeira archipelago; island: Madeira; country: Portugal; countryCode: PT; stateProvince: Madeira; county: Santana; locality: Fajã da Nogueira - Til Gigante; verbatimElevation: 841; decimalLatitude: 32.7457; decimalLongitude: -16.915; geodeticDatum: WGS84; **Event:** samplingProtocol: Direct sampling
- **Type status:** Other material. **Occurrence:** sex: 2 males, 3 females; **Location:** locationID: 8; higherGeography: Macaronesia; continent: Europe; waterBody: Atlantic Ocean; islandGroup: Madeira archipelago; island: Madeira; country: Portugal; countryCode: PT; stateProvince: Madeira; county: Santana; locality: Fajã da Nogueira - Til Gigante; verbatimElevation: 841; decimalLatitude: 32.7457; decimalLongitude: -16.915; geodeticDatum: WGS84; **Event:** samplingProtocol: Pitfall
- **Type status:** Other material. **Occurrence:** sex: 1 male, 3 females; **Location:** locationID: 9; higherGeography: Macaronesia; continent: Europe; waterBody: Atlantic Ocean; islandGroup: Madeira archipelago; island: Madeira; country: Portugal; countryCode: PT; stateProvince: Madeira; county: Santana; locality: Queimadas; verbatimElevation: 841; decimalLatitude: 32.7873; decimalLongitude: -16.9047; geodeticDatum: WGS84; **Event:** samplingProtocol: Direct sampling
- **Type status:** Other material. **Occurrence:** sex: 2 females; **Location:** locationID: 9; higherGeography: Macaronesia; continent: Europe; waterBody: Atlantic Ocean; islandGroup: Madeira archipelago; island: Madeira; country: Portugal; countryCode: PT; stateProvince: Madeira; county: Santana; locality: Queimadas; verbatimElevation: 841; decimalLatitude: 32.7873; decimalLongitude: -16.9047; geodeticDatum: WGS84; **Event:** samplingProtocol: Pitfall
- **Type status:** Other material. **Occurrence:** sex: 2 males, 1 female; **Location:** locationID: 12; higherGeography: Macaronesia; continent: Europe; waterBody: Atlantic Ocean; islandGroup: Madeira archipelago; island: Madeira; country: Portugal; countryCode: PT; stateProvince: Madeira; county: Santana; locality: Achada do Teixeira; verbatimElevation: 1103; decimalLatitude: 32.7762; decimalLongitude: -16.9022; geodeticDatum: WGS84; **Event:** samplingProtocol: Pitfall
- **Type status:** Other material. **Occurrence:** sex: 1 female; **Location:** locationID: 13; higherGeography: Macaronesia; continent: Europe; waterBody: Atlantic Ocean; islandGroup: Madeira archipelago; island: Madeira; country: Portugal; countryCode: PT; stateProvince: Madeira; county: Santana; locality: Ribeiro Frio - Viveiro; verbatimElevation: 906; decimalLatitude: 32.7354; decimalLongitude: -16.8864; geodeticDatum: WGS84; **Event:** samplingProtocol: Direct sampling
- **Type status:** Other material. **Occurrence:** sex: 1 female; **Location:** locationID: 14; higherGeography: Macaronesia; continent: Europe; waterBody: Atlantic Ocean; islandGroup: Madeira archipelago; island: Madeira; country: Portugal; countryCode: PT; stateProvince: Madeira; county: Santana; locality: Ribeiro Frio - Cottages; verbatimElevation: 994; decimalLatitude: 32.7319; decimalLongitude: -16.8861; geodeticDatum: WGS84; **Event:** samplingProtocol: Direct sampling
- **Type status:** Other material. **Occurrence:** sex: 3 males, 2 females; **Location:** locationID: 14; higherGeography: Macaronesia; continent: Europe; waterBody: Atlantic Ocean; islandGroup: Madeira archipelago; island: Madeira; country: Portugal; countryCode: PT; stateProvince: Madeira; county: Santana; locality: Ribeiro Frio - Cottages; verbatimElevation: 994; decimalLatitude: 32.7319; decimalLongitude: -16.8861; geodeticDatum: WGS84; **Event:** samplingProtocol: Pitfall
- **Type status:** Other material. **Occurrence:** sex: 1 female; **Location:** locationID: 16; higherGeography: Macaronesia; continent: Europe; waterBody: Atlantic Ocean; islandGroup: Madeira archipelago; island: Madeira; country: Portugal; countryCode: PT; stateProvince: Madeira; county: Santana; locality: Pico do Areeiro; verbatimElevation: 1594; decimalLatitude: 32.7287; decimalLongitude: -16.9202; geodeticDatum: WGS84; **Event:** samplingProtocol: Pitfall
- **Type status:** Other material. **Occurrence:** sex: 5 males, 8 females; **Location:** locationID: 17; higherGeography: Macaronesia; continent: Europe; waterBody: Atlantic Ocean; islandGroup: Madeira archipelago; island: Madeira; country: Portugal; countryCode: PT; stateProvince: Madeira; county: Porto Moniz; locality: Chão da Ribeira; verbatimElevation: 519; decimalLatitude: 32.7933; decimalLongitude: -17.1122; geodeticDatum: WGS84; **Event:** samplingProtocol: Pitfall
- **Type status:** Other material. **Occurrence:** sex: 1 male, 15 females; **Location:** locationID: 18; higherGeography: Macaronesia; continent: Europe; waterBody: Atlantic Ocean; islandGroup: Madeira archipelago; island: Madeira; country: Portugal; countryCode: PT; stateProvince: Madeira; county: Porto Moniz; locality: Chão da Ribeira; verbatimElevation: 491; decimalLatitude: 32.7957; decimalLongitude: -17.1117; geodeticDatum: WGS84; **Event:** samplingProtocol: Direct sampling
- **Type status:** Other material. **Occurrence:** sex: 4 males, 1 female; **Location:** locationID: 18; higherGeography: Macaronesia; continent: Europe; waterBody: Atlantic Ocean; islandGroup: Madeira archipelago; island: Madeira; country: Portugal; countryCode: PT; stateProvince: Madeira; county: Porto Moniz; locality: Chão da Ribeira; verbatimElevation: 491; decimalLatitude: 32.7957; decimalLongitude: -17.1117; geodeticDatum: WGS84; **Event:** samplingProtocol: Pitfall
- **Type status:** Other material. **Occurrence:** sex: 1 male, 2 females; **Location:** locationID: 19; higherGeography: Macaronesia; continent: Europe; waterBody: Atlantic Ocean; islandGroup: Madeira archipelago; island: Madeira; country: Portugal; countryCode: PT; stateProvince: Madeira; county: São Vicente; locality: Chão dos Louros; verbatimElevation: 748; decimalLatitude: 32.7636; decimalLongitude: -17.019; geodeticDatum: WGS84; **Event:** samplingProtocol: Pitfall
- **Type status:** Other material. **Occurrence:** sex: 2 males, 4 females; **Location:** locationID: 20; higherGeography: Macaronesia; continent: Europe; waterBody: Atlantic Ocean; islandGroup: Madeira archipelago; island: Madeira; country: Portugal; countryCode: PT; stateProvince: Madeira; county: São Vicente; locality: Encumeada; verbatimElevation: 999; decimalLatitude: 32.7558; decimalLongitude: -17.0143; geodeticDatum: WGS84; **Event:** samplingProtocol: Pitfall
- **Type status:** Other material. **Occurrence:** sex: 6 males, 18 females; **Location:** locationID: 21; higherGeography: Macaronesia; continent: Europe; waterBody: Atlantic Ocean; islandGroup: Madeira archipelago; island: Madeira; country: Portugal; countryCode: PT; stateProvince: Madeira; county: Santana; locality: Ribeiro Bonito - Levada; verbatimElevation: 568; decimalLatitude: 32.8047; decimalLongitude: -16.9346; geodeticDatum: WGS84; **Event:** samplingProtocol: Direct sampling
- **Type status:** Other material. **Occurrence:** sex: 4 females; **Location:** locationID: 21; higherGeography: Macaronesia; continent: Europe; waterBody: Atlantic Ocean; islandGroup: Madeira archipelago; island: Madeira; country: Portugal; countryCode: PT; stateProvince: Madeira; county: Santana; locality: Ribeiro Bonito - Levada; verbatimElevation: 568; decimalLatitude: 32.8047; decimalLongitude: -16.9346; geodeticDatum: WGS84; **Event:** samplingProtocol: Pitfall
- **Type status:** Other material. **Occurrence:** sex: 1 male, 13 females; **Location:** locationID: 22; higherGeography: Macaronesia; continent: Europe; waterBody: Atlantic Ocean; islandGroup: Madeira archipelago; island: Madeira; country: Portugal; countryCode: PT; stateProvince: Madeira; county: Santana; locality: Ribeiro Bonito - Ribeiro; verbatimElevation: 560; decimalLatitude: 32.7985; decimalLongitude: -16.936; geodeticDatum: WGS84; **Event:** samplingProtocol: Direct sampling
- **Type status:** Other material. **Occurrence:** sex: 2 males, 2 females; **Location:** locationID: 22; higherGeography: Macaronesia; continent: Europe; waterBody: Atlantic Ocean; islandGroup: Madeira archipelago; island: Madeira; country: Portugal; countryCode: PT; stateProvince: Madeira; county: Santana; locality: Ribeiro Bonito - Ribeiro; verbatimElevation: 560; decimalLatitude: 32.7985; decimalLongitude: -16.936; geodeticDatum: WGS84; **Event:** samplingProtocol: Pitfall
- **Type status:** Other material. **Occurrence:** sex: 1 male, 1 female; **Location:** locationID: 23; higherGeography: Macaronesia; continent: Europe; waterBody: Atlantic Ocean; islandGroup: Madeira archipelago; island: Madeira; country: Portugal; countryCode: PT; stateProvince: Madeira; county: Porto Moniz; locality: Fanal; verbatimElevation: 755; decimalLatitude: 32.8302; decimalLongitude: -17.1585; geodeticDatum: WGS84; **Event:** samplingProtocol: Pitfall
- **Type status:** Other material. **Occurrence:** sex: 2 females; **Location:** locationID: 24; higherGeography: Macaronesia; continent: Europe; waterBody: Atlantic Ocean; islandGroup: Madeira archipelago; island: Madeira; country: Portugal; countryCode: PT; stateProvince: Madeira; county: Porto Moniz; locality: Fanal - Levada dos Cedros; verbatimElevation: 820; decimalLatitude: 32.8259; decimalLongitude: -17.158; geodeticDatum: WGS84; **Event:** samplingProtocol: Direct sampling
- **Type status:** Other material. **Occurrence:** sex: 9 males, 3 females; **Location:** locationID: 24; higherGeography: Macaronesia; continent: Europe; waterBody: Atlantic Ocean; islandGroup: Madeira archipelago; island: Madeira; country: Portugal; countryCode: PT; stateProvince: Madeira; county: Porto Moniz; locality: Fanal - Levada dos Cedros; verbatimElevation: 820; decimalLatitude: 32.8259; decimalLongitude: -17.158; geodeticDatum: WGS84; **Event:** samplingProtocol: Pitfall
- **Type status:** Other material. **Occurrence:** sex: 1 male, 3 females; **Location:** locationID: 25; higherGeography: Macaronesia; continent: Europe; waterBody: Atlantic Ocean; islandGroup: Madeira archipelago; island: Madeira; country: Portugal; countryCode: PT; stateProvince: Madeira; county: Porto Moniz; locality: Fanal; verbatimElevation: 890; decimalLatitude: 32.8236; decimalLongitude: -17.156; geodeticDatum: WGS84; **Event:** samplingProtocol: Pitfall
- **Type status:** Other material. **Occurrence:** sex: 1 male; **Location:** locationID: 26; higherGeography: Macaronesia; continent: Europe; waterBody: Atlantic Ocean; islandGroup: Madeira archipelago; island: Madeira; country: Portugal; countryCode: PT; stateProvince: Madeira; county: Porto Moniz; locality: Fanal; verbatimElevation: 889; decimalLatitude: 32.8226; decimalLongitude: -17.1539; geodeticDatum: WGS84; **Event:** samplingProtocol: Pitfall
- **Type status:** Other material. **Occurrence:** sex: 1 female; **Location:** locationID: 27; higherGeography: Macaronesia; continent: Europe; waterBody: Atlantic Ocean; islandGroup: Madeira archipelago; island: Madeira; country: Portugal; countryCode: PT; stateProvince: Madeira; county: Porto Moniz; locality: Fanal; verbatimElevation: 1023; decimalLatitude: 32.8182; decimalLongitude: -17.1521; geodeticDatum: WGS84; **Event:** samplingProtocol: Direct sampling
- **Type status:** Other material. **Occurrence:** sex: 2 males, 2 females; **Location:** locationID: 27; higherGeography: Macaronesia; continent: Europe; waterBody: Atlantic Ocean; islandGroup: Madeira archipelago; island: Madeira; country: Portugal; countryCode: PT; stateProvince: Madeira; county: Porto Moniz; locality: Fanal; verbatimElevation: 1023; decimalLatitude: 32.8182; decimalLongitude: -17.1521; geodeticDatum: WGS84; **Event:** samplingProtocol: Pitfall
- **Type status:** Other material. **Occurrence:** sex: 1 male; **Location:** locationID: 28; higherGeography: Macaronesia; continent: Europe; waterBody: Atlantic Ocean; islandGroup: Madeira archipelago; island: Madeira; country: Portugal; countryCode: PT; stateProvince: Madeira; county: Porto Moniz; locality: Fanal; verbatimElevation: 1134; decimalLatitude: 32.8062; decimalLongitude: -17.1409; geodeticDatum: WGS84; **Event:** samplingProtocol: Direct sampling
- **Type status:** Other material. **Occurrence:** sex: 4 males, 2 females; **Location:** locationID: 28; higherGeography: Macaronesia; continent: Europe; waterBody: Atlantic Ocean; islandGroup: Madeira archipelago; island: Madeira; country: Portugal; countryCode: PT; stateProvince: Madeira; county: Porto Moniz; locality: Fanal; verbatimElevation: 1134; decimalLatitude: 32.8062; decimalLongitude: -17.1409; geodeticDatum: WGS84; **Event:** samplingProtocol: Pitfall
- **Type status:** Other material. **Occurrence:** sex: 2 males, 2 females; **Location:** locationID: 29; higherGeography: Macaronesia; continent: Europe; waterBody: Atlantic Ocean; islandGroup: Madeira archipelago; island: Madeira; country: Portugal; countryCode: PT; stateProvince: Madeira; county: São Vicente; locality: Ginjas; verbatimElevation: 869; decimalLatitude: 32.7758; decimalLongitude: -17.0534; geodeticDatum: WGS84; **Event:** samplingProtocol: Direct sampling
- **Type status:** Other material. **Occurrence:** sex: 3 females; **Location:** locationID: 29; higherGeography: Macaronesia; continent: Europe; waterBody: Atlantic Ocean; islandGroup: Madeira archipelago; island: Madeira; country: Portugal; countryCode: PT; stateProvince: Madeira; county: São Vicente; locality: Ginjas; verbatimElevation: 869; decimalLatitude: 32.7758; decimalLongitude: -17.0534; geodeticDatum: WGS84; **Event:** samplingProtocol: Pitfall
- **Type status:** Other material. **Occurrence:** sex: 2 females; **Location:** locationID: 30; higherGeography: Macaronesia; continent: Europe; waterBody: Atlantic Ocean; islandGroup: Madeira archipelago; island: Madeira; country: Portugal; countryCode: PT; stateProvince: Madeira; county: São Vicente; locality: Caramujo; verbatimElevation: 981; decimalLatitude: 32.7722; decimalLongitude: -17.0529; geodeticDatum: WGS84; **Event:** samplingProtocol: Pitfall
- **Type status:** Other material. **Occurrence:** sex: 1 female; **Location:** locationID: 32; higherGeography: Macaronesia; continent: Europe; waterBody: Atlantic Ocean; islandGroup: Madeira archipelago; island: Madeira; country: Portugal; countryCode: PT; stateProvince: Madeira; county: Calheta; locality: Rabaças; verbatimElevation: 993; decimalLatitude: 32.7413; decimalLongitude: -17.0783; geodeticDatum: WGS84; **Event:** samplingProtocol: Direct sampling
- **Type status:** Other material. **Occurrence:** sex: 5 males, 13 females; **Location:** locationID: 32; higherGeography: Macaronesia; continent: Europe; waterBody: Atlantic Ocean; islandGroup: Madeira archipelago; island: Madeira; country: Portugal; countryCode: PT; stateProvince: Madeira; county: Calheta; locality: Rabaças; verbatimElevation: 993; decimalLatitude: 32.7413; decimalLongitude: -17.0783; geodeticDatum: WGS84; **Event:** samplingProtocol: Pitfall
- **Type status:** Other material. **Occurrence:** sex: 2 males, 3 females; **Location:** locationID: 33; higherGeography: Macaronesia; continent: Europe; waterBody: Atlantic Ocean; islandGroup: Madeira archipelago; island: Madeira; country: Portugal; countryCode: PT; stateProvince: Madeira; county: Porto Moniz; locality: Rabaçal; verbatimElevation: 930; decimalLatitude: 32.7647; decimalLongitude: -17.1341; geodeticDatum: WGS84; **Event:** samplingProtocol: Direct sampling
- **Type status:** Other material. **Occurrence:** sex: 5 males, 2 females; **Location:** locationID: 33; higherGeography: Macaronesia; continent: Europe; waterBody: Atlantic Ocean; islandGroup: Madeira archipelago; island: Madeira; country: Portugal; countryCode: PT; stateProvince: Madeira; county: Porto Moniz; locality: Rabaçal; verbatimElevation: 930; decimalLatitude: 32.7647; decimalLongitude: -17.1341; geodeticDatum: WGS84; **Event:** samplingProtocol: Pitfall
- **Type status:** Other material. **Occurrence:** sex: 2 males, 2 females; **Location:** locationID: 34; higherGeography: Macaronesia; continent: Europe; waterBody: Atlantic Ocean; islandGroup: Madeira archipelago; island: Madeira; country: Portugal; countryCode: PT; stateProvince: Madeira; county: Ponta do Sol; locality: Risco; verbatimElevation: 1048; decimalLatitude: 32.7608; decimalLongitude: -17.1256; geodeticDatum: WGS84; **Event:** samplingProtocol: Direct sampling
- **Type status:** Other material. **Occurrence:** sex: 4 females; **Location:** locationID: 34; higherGeography: Macaronesia; continent: Europe; waterBody: Atlantic Ocean; islandGroup: Madeira archipelago; island: Madeira; country: Portugal; countryCode: PT; stateProvince: Madeira; county: Ponta do Sol; locality: Risco; verbatimElevation: 1048; decimalLatitude: 32.7608; decimalLongitude: -17.1256; geodeticDatum: WGS84; **Event:** samplingProtocol: Pitfall
- **Type status:** Other material. **Occurrence:** sex: 1 male, 2 females; **Location:** locationID: 35; higherGeography: Macaronesia; continent: Europe; waterBody: Atlantic Ocean; islandGroup: Madeira archipelago; island: Madeira; country: Portugal; countryCode: PT; stateProvince: Madeira; county: Porto Moniz; locality: Casa do Elias; verbatimElevation: 814; decimalLatitude: 32.8268; decimalLongitude: -17.1883; geodeticDatum: WGS84; **Event:** samplingProtocol: Pitfall
- **Type status:** Other material. **Occurrence:** sex: 6 males, 6 females; **Location:** locationID: 36; higherGeography: Macaronesia; continent: Europe; waterBody: Atlantic Ocean; islandGroup: Madeira archipelago; island: Madeira; country: Portugal; countryCode: PT; stateProvince: Madeira; county: Calheta; locality: Galhano; verbatimElevation: 975; decimalLatitude: 32.7971; decimalLongitude: -17.1729; geodeticDatum: WGS84; **Event:** samplingProtocol: Pitfall

##### Ecological interactions

###### Native status

introduced

##### Distribution

Palearctic (introduced, elsewhere)

#### Turinyphia maderiana

(Schenkel, 1938)

##### Materials

- **Type status:** Other material. **Occurrence:** sex: 1 male, 1 female; **Location:** locationID: 4; higherGeography: Macaronesia; continent: Europe; waterBody: Atlantic Ocean; islandGroup: Madeira archipelago; island: Madeira; country: Portugal; countryCode: PT; stateProvince: Madeira; county: Santana; locality: Fajã da Nogueira - Mtdo. do Leacoque; verbatimElevation: 630; decimalLatitude: 32.7415; decimalLongitude: -16.9161; geodeticDatum: WGS84; **Event:** samplingProtocol: Pitfall
- **Type status:** Other material. **Occurrence:** sex: 1 female; **Location:** locationID: 7; higherGeography: Macaronesia; continent: Europe; waterBody: Atlantic Ocean; islandGroup: Madeira archipelago; island: Madeira; country: Portugal; countryCode: PT; stateProvince: Madeira; county: Santana; locality: Fajã da Nogueira - Tanque; verbatimElevation: 845; decimalLatitude: 32.7425; decimalLongitude: -16.9168; geodeticDatum: WGS84; **Event:** samplingProtocol: Direct sampling
- **Type status:** Other material. **Occurrence:** sex: 1 male; **Location:** locationID: 20; higherGeography: Macaronesia; continent: Europe; waterBody: Atlantic Ocean; islandGroup: Madeira archipelago; island: Madeira; country: Portugal; countryCode: PT; stateProvince: Madeira; county: São Vicente; locality: Encumeada; verbatimElevation: 999; decimalLatitude: 32.7558; decimalLongitude: -17.0143; geodeticDatum: WGS84; **Event:** samplingProtocol: Pitfall
- **Type status:** Other material. **Occurrence:** sex: 1 female; **Location:** locationID: 22; higherGeography: Macaronesia; continent: Europe; waterBody: Atlantic Ocean; islandGroup: Madeira archipelago; island: Madeira; country: Portugal; countryCode: PT; stateProvince: Madeira; county: Santana; locality: Ribeiro Bonito - Ribeiro; verbatimElevation: 560; decimalLatitude: 32.7985; decimalLongitude: -16.936; geodeticDatum: WGS84; **Event:** samplingProtocol: Direct sampling
- **Type status:** Other material. **Occurrence:** sex: 1 male; **Location:** locationID: 30; higherGeography: Macaronesia; continent: Europe; waterBody: Atlantic Ocean; islandGroup: Madeira archipelago; island: Madeira; country: Portugal; countryCode: PT; stateProvince: Madeira; county: São Vicente; locality: Caramujo; verbatimElevation: 981; decimalLatitude: 32.7722; decimalLongitude: -17.0529; geodeticDatum: WGS84; **Event:** samplingProtocol: Pitfall
- **Type status:** Other material. **Occurrence:** sex: 1 female; **Location:** locationID: 33; higherGeography: Macaronesia; continent: Europe; waterBody: Atlantic Ocean; islandGroup: Madeira archipelago; island: Madeira; country: Portugal; countryCode: PT; stateProvince: Madeira; county: Porto Moniz; locality: Rabaçal; verbatimElevation: 930; decimalLatitude: 32.7647; decimalLongitude: -17.1341; geodeticDatum: WGS84; **Event:** samplingProtocol: Pitfall

##### Ecological interactions

###### Native status

SIE

##### Distribution

Madeira island (Fig. 3d)

##### Notes

This is the third record of this species. *Turinyphia maderiana* seems to be restricted to Laurisilva.

#### Typhochrestus madeirensis

Crespo, 2013

##### Materials

- **Type status:** Holotype. **Occurrence:** sex: 1 male; **Location:** locationID: 16; higherGeography: Macaronesia; continent: Europe; waterBody: Atlantic Ocean; islandGroup: Madeira archipelago; island: Madeira; country: Portugal; countryCode: PT; stateProvince: Madeira; county: Santana; locality: Pico do Areeiro; verbatimElevation: 1594; decimalLatitude: 32.7287; decimalLongitude: -16.9202; geodeticDatum: WGS84; **Event:** samplingProtocol: Pitfall

##### Ecological interactions

###### Native status

archipelago endemic

##### Distribution

Madeira island, Deserta Grande island

##### Notes

This species was recently described (Crespo et al. 2013), from native arid or semi-arid meadows. In Madeira archipelago this kind of habitat is usually found at high altitude, as in Pico do Areeiro where the species was collected.

#### Unidentified species

##### Materials

- **Type status:** Other material. **Occurrence:** sex: 1 male, 1 female; **Location:** locationID: 32; higherGeography: Macaronesia; continent: Europe; waterBody: Atlantic Ocean; islandGroup: Madeira archipelago; island: Madeira; country: Portugal; countryCode: PT; stateProvince: Madeira; county: Calheta; locality: Rabaças; verbatimElevation: 993; decimalLatitude: 32.7413; decimalLongitude: -17.0783; geodeticDatum: WGS84; **Event:** samplingProtocol: Pitfall

##### Ecological interactions

###### Native status

native

##### Distribution

Madeira island, Porto Santo island

##### Notes

Although this is clearly a “micronetine”, species identification was impossible.

#### Pardosa proxima

(C. L. Koch, 1847)

##### Materials

- **Type status:** Other material. **Occurrence:** sex: 6 females; **Location:** locationID: 15; higherGeography: Macaronesia; continent: Europe; waterBody: Atlantic Ocean; islandGroup: Madeira archipelago; island: Madeira; country: Portugal; countryCode: PT; stateProvince: Madeira; county: Santana; locality: Pico do Areeiro; verbatimElevation: 1533; decimalLatitude: 32.7231; decimalLongitude: -16.9109; geodeticDatum: WGS84; **Event:** samplingProtocol: Pitfall
- **Type status:** Other material. **Occurrence:** sex: 11 males, 51 females; **Location:** locationID: 16; higherGeography: Macaronesia; continent: Europe; waterBody: Atlantic Ocean; islandGroup: Madeira archipelago; island: Madeira; country: Portugal; countryCode: PT; stateProvince: Madeira; county: Santana; locality: Pico do Areeiro; verbatimElevation: 1594; decimalLatitude: 32.7287; decimalLongitude: -16.9202; geodeticDatum: WGS84; **Event:** samplingProtocol: Pitfall

##### Ecological interactions

###### Native status

introduced

##### Distribution

Palearctic, Canary Islands, Azores, Madeira archipelago

#### Ero aphana

(Walckenaer, 1802)

##### Materials

- **Type status:** Other material. **Occurrence:** sex: 1 male; **Location:** locationID: 2; higherGeography: Macaronesia; continent: Europe; waterBody: Atlantic Ocean; islandGroup: Madeira archipelago; island: Madeira; country: Portugal; countryCode: PT; stateProvince: Madeira; county: Machico; locality: Funduras; verbatimElevation: 552; decimalLatitude: 32.754; decimalLongitude: -16.8099; geodeticDatum: WGS84; **Event:** samplingProtocol: Direct sampling

##### Ecological interactions

###### Native status

introduced

##### Distribution

Palearctic (St. Helena, Queensland, Western Australia, introduced)

#### Cheiracanthium albidulum

(Blackwall, 1859)

##### Materials

- **Type status:** Other material. **Occurrence:** sex: 1 female; **Location:** locationID: 2; higherGeography: Macaronesia; continent: Europe; waterBody: Atlantic Ocean; islandGroup: Madeira archipelago; island: Madeira; country: Portugal; countryCode: PT; stateProvince: Madeira; county: Machico; locality: Funduras; verbatimElevation: 552; decimalLatitude: 32.754; decimalLongitude: -16.8099; geodeticDatum: WGS84; **Event:** samplingProtocol: Direct sampling
- **Type status:** Other material. **Occurrence:** sex: 1 female; **Location:** locationID: 6; higherGeography: Macaronesia; continent: Europe; waterBody: Atlantic Ocean; islandGroup: Madeira archipelago; island: Madeira; country: Portugal; countryCode: PT; stateProvince: Madeira; county: Santana; locality: Fajã da Nogueira - Mtdo. do Leacoque; verbatimElevation: 614; decimalLatitude: 32.7418; decimalLongitude: -16.9177; geodeticDatum: WGS84; **Event:** samplingProtocol: Direct sampling
- **Type status:** Other material. **Occurrence:** sex: 1 female; **Location:** locationID: 12; higherGeography: Macaronesia; continent: Europe; waterBody: Atlantic Ocean; islandGroup: Madeira archipelago; island: Madeira; country: Portugal; countryCode: PT; stateProvince: Madeira; county: Santana; locality: Achada do Teixeira; verbatimElevation: 1103; decimalLatitude: 32.7762; decimalLongitude: -16.9022; geodeticDatum: WGS84; **Event:** samplingProtocol: Direct sampling
- **Type status:** Other material. **Occurrence:** sex: 1 female; **Location:** locationID: 13; higherGeography: Macaronesia; continent: Europe; waterBody: Atlantic Ocean; islandGroup: Madeira archipelago; island: Madeira; country: Portugal; countryCode: PT; stateProvince: Madeira; county: Santana; locality: Ribeiro Frio - Viveiro; verbatimElevation: 906; decimalLatitude: 32.7354; decimalLongitude: -16.8864; geodeticDatum: WGS84; **Event:** samplingProtocol: Direct sampling
- **Type status:** Other material. **Occurrence:** sex: 4 males, 1 female; **Location:** locationID: 24; higherGeography: Macaronesia; continent: Europe; waterBody: Atlantic Ocean; islandGroup: Madeira archipelago; island: Madeira; country: Portugal; countryCode: PT; stateProvince: Madeira; county: Porto Moniz; locality: Fanal - Levada dos Cedros; verbatimElevation: 820; decimalLatitude: 32.8259; decimalLongitude: -17.158; geodeticDatum: WGS84; **Event:** samplingProtocol: Direct sampling
- **Type status:** Other material. **Occurrence:** sex: 1 female; **Location:** locationID: 28; higherGeography: Macaronesia; continent: Europe; waterBody: Atlantic Ocean; islandGroup: Madeira archipelago; island: Madeira; country: Portugal; countryCode: PT; stateProvince: Madeira; county: Porto Moniz; locality: Fanal; verbatimElevation: 1134; decimalLatitude: 32.8062; decimalLongitude: -17.1409; geodeticDatum: WGS84; **Event:** samplingProtocol: Direct sampling

##### Ecological interactions

###### Native status

macaronesian endemic

##### Distribution

Madeira archipelago, Canary Islands

#### Trogloneta madeirensis

Wunderlich, 1987

##### Materials

- **Type status:** Other material. **Occurrence:** sex: 1 female; **Location:** locationID: 1; higherGeography: Macaronesia; continent: Europe; waterBody: Atlantic Ocean; islandGroup: Madeira archipelago; island: Madeira; country: Portugal; countryCode: PT; stateProvince: Madeira; county: Machico; locality: Funduras; verbatimElevation: 500; decimalLatitude: 32.7493; decimalLongitude: -16.8114; geodeticDatum: WGS84; **Event:** samplingProtocol: Direct sampling
- **Type status:** Other material. **Occurrence:** sex: 4 females; **Location:** locationID: 4; higherGeography: Macaronesia; continent: Europe; waterBody: Atlantic Ocean; islandGroup: Madeira archipelago; island: Madeira; country: Portugal; countryCode: PT; stateProvince: Madeira; county: Santana; locality: Fajã da Nogueira - Mtdo. do Leacoque; verbatimElevation: 630; decimalLatitude: 32.7415; decimalLongitude: -16.9161; geodeticDatum: WGS84; **Event:** samplingProtocol: Direct sampling
- **Type status:** Other material. **Occurrence:** sex: 2 females; **Location:** locationID: 6; higherGeography: Macaronesia; continent: Europe; waterBody: Atlantic Ocean; islandGroup: Madeira archipelago; island: Madeira; country: Portugal; countryCode: PT; stateProvince: Madeira; county: Santana; locality: Fajã da Nogueira - Mtdo. do Leacoque; verbatimElevation: 614; decimalLatitude: 32.7418; decimalLongitude: -16.9177; geodeticDatum: WGS84; **Event:** samplingProtocol: Direct sampling
- **Type status:** Other material. **Occurrence:** sex: 1 male, 2 females; **Location:** locationID: 7; higherGeography: Macaronesia; continent: Europe; waterBody: Atlantic Ocean; islandGroup: Madeira archipelago; island: Madeira; country: Portugal; countryCode: PT; stateProvince: Madeira; county: Santana; locality: Fajã da Nogueira - Tanque; verbatimElevation: 845; decimalLatitude: 32.7425; decimalLongitude: -16.9168; geodeticDatum: WGS84; **Event:** samplingProtocol: Direct sampling
- **Type status:** Other material. **Occurrence:** sex: 1 female; **Location:** locationID: 8; higherGeography: Macaronesia; continent: Europe; waterBody: Atlantic Ocean; islandGroup: Madeira archipelago; island: Madeira; country: Portugal; countryCode: PT; stateProvince: Madeira; county: Santana; locality: Fajã da Nogueira - Til Gigante; verbatimElevation: 841; decimalLatitude: 32.7457; decimalLongitude: -16.915; geodeticDatum: WGS84; **Event:** samplingProtocol: Direct sampling
- **Type status:** Other material. **Occurrence:** sex: 1 female; **Location:** locationID: 11; higherGeography: Macaronesia; continent: Europe; waterBody: Atlantic Ocean; islandGroup: Madeira archipelago; island: Madeira; country: Portugal; countryCode: PT; stateProvince: Madeira; county: Santana; locality: Achada do Teixeira; verbatimElevation: 1211; decimalLatitude: 32.7733; decimalLongitude: -16.9081; geodeticDatum: WGS84; **Event:** samplingProtocol: Direct sampling
- **Type status:** Other material. **Occurrence:** sex: 1 female; **Location:** locationID: 14; higherGeography: Macaronesia; continent: Europe; waterBody: Atlantic Ocean; islandGroup: Madeira archipelago; island: Madeira; country: Portugal; countryCode: PT; stateProvince: Madeira; county: Santana; locality: Ribeiro Frio - Cottages; verbatimElevation: 994; decimalLatitude: 32.7319; decimalLongitude: -16.8861; geodeticDatum: WGS84; **Event:** samplingProtocol: Direct sampling
- **Type status:** Other material. **Occurrence:** sex: 3 females; **Location:** locationID: 18; higherGeography: Macaronesia; continent: Europe; waterBody: Atlantic Ocean; islandGroup: Madeira archipelago; island: Madeira; country: Portugal; countryCode: PT; stateProvince: Madeira; county: Porto Moniz; locality: Chão da Ribeira; verbatimElevation: 491; decimalLatitude: 32.7957; decimalLongitude: -17.1117; geodeticDatum: WGS84; **Event:** samplingProtocol: Direct sampling
- **Type status:** Other material. **Occurrence:** sex: 4 males, 5 females; **Location:** locationID: 21; higherGeography: Macaronesia; continent: Europe; waterBody: Atlantic Ocean; islandGroup: Madeira archipelago; island: Madeira; country: Portugal; countryCode: PT; stateProvince: Madeira; county: Santana; locality: Ribeiro Bonito - Levada; verbatimElevation: 568; decimalLatitude: 32.8047; decimalLongitude: -16.9346; geodeticDatum: WGS84; **Event:** samplingProtocol: Direct sampling
- **Type status:** Other material. **Occurrence:** sex: 1 male, 1 female; **Location:** locationID: 22; higherGeography: Macaronesia; continent: Europe; waterBody: Atlantic Ocean; islandGroup: Madeira archipelago; island: Madeira; country: Portugal; countryCode: PT; stateProvince: Madeira; county: Santana; locality: Ribeiro Bonito - Ribeiro; verbatimElevation: 560; decimalLatitude: 32.7985; decimalLongitude: -16.936; geodeticDatum: WGS84; **Event:** samplingProtocol: Direct sampling
- **Type status:** Other material. **Occurrence:** sex: 1 male; **Location:** locationID: 25; higherGeography: Macaronesia; continent: Europe; waterBody: Atlantic Ocean; islandGroup: Madeira archipelago; island: Madeira; country: Portugal; countryCode: PT; stateProvince: Madeira; county: Porto Moniz; locality: Fanal; verbatimElevation: 890; decimalLatitude: 32.8236; decimalLongitude: -17.156; geodeticDatum: WGS84; **Event:** samplingProtocol: Direct sampling
- **Type status:** Other material. **Occurrence:** sex: 2 females; **Location:** locationID: 29; higherGeography: Macaronesia; continent: Europe; waterBody: Atlantic Ocean; islandGroup: Madeira archipelago; island: Madeira; country: Portugal; countryCode: PT; stateProvince: Madeira; county: São Vicente; locality: Ginjas; verbatimElevation: 869; decimalLatitude: 32.7758; decimalLongitude: -17.0534; geodeticDatum: WGS84; **Event:** samplingProtocol: Direct sampling

##### Ecological interactions

###### Native status

SIE

##### Distribution

Madeira island (Fig. 4a)

##### Notes

This species seems to be restricted to Laurisilva. Wunderlich (Wunderlich 1987) described *Trogloneta madeirensis* from only two sites, but the present study considerably enlarges the distribution of this endemic species throughout the Laurisilva of Madeira island.

#### Philodromus insulanus

Kulczynski, 1905

##### Materials

- **Type status:** Other material. **Occurrence:** sex: 2 females; **Location:** locationID: 28; higherGeography: Macaronesia; continent: Europe; waterBody: Atlantic Ocean; islandGroup: Madeira archipelago; island: Madeira; country: Portugal; countryCode: PT; stateProvince: Madeira; county: Porto Moniz; locality: Fanal; verbatimElevation: 1134; decimalLatitude: 32.8062; decimalLongitude: -17.1409; geodeticDatum: WGS84; **Event:** samplingProtocol: Direct sampling

##### Ecological interactions

###### Native status

archipelago endemic

##### Distribution

Madeira island, Porto Santo island

#### Thanatus oblongiusculus

(Lucas, 1846)

##### Materials

- **Type status:** Other material. **Occurrence:** sex: 1 female; **Location:** locationID: 32; higherGeography: Macaronesia; continent: Europe; waterBody: Atlantic Ocean; islandGroup: Madeira archipelago; island: Madeira; country: Portugal; countryCode: PT; stateProvince: Madeira; county: Calheta; locality: Rabaças; verbatimElevation: 993; decimalLatitude: 32.7413; decimalLongitude: -17.0783; geodeticDatum: WGS84; **Event:** samplingProtocol: Pitfall

##### Ecological interactions

###### Native status

introduced

##### Distribution

Palearctic

#### Chalcoscirtus sublestus

(Blackwall, 1867)

##### Materials

- **Type status:** Other material. **Occurrence:** sex: 5 males, 4 females; **Location:** locationID: 15; higherGeography: Macaronesia; continent: Europe; waterBody: Atlantic Ocean; islandGroup: Madeira archipelago; island: Madeira; country: Portugal; countryCode: PT; stateProvince: Madeira; county: Santana; locality: Pico do Areeiro; verbatimElevation: 1533; decimalLatitude: 32.7231; decimalLongitude: -16.9109; geodeticDatum: WGS84; **Event:** samplingProtocol: Pitfall
- **Type status:** Other material. **Occurrence:** sex: 1 male, 4 females; **Location:** locationID: 16; higherGeography: Macaronesia; continent: Europe; waterBody: Atlantic Ocean; islandGroup: Madeira archipelago; island: Madeira; country: Portugal; countryCode: PT; stateProvince: Madeira; county: Santana; locality: Pico do Areeiro; verbatimElevation: 1594; decimalLatitude: 32.7287; decimalLongitude: -16.9202; geodeticDatum: WGS84; **Event:** samplingProtocol: Pitfall

##### Ecological interactions

###### Native status

macaronesian endemic

##### Distribution

Madeira archipelago, Canary Islands

#### Macaroeris diligens

(Blackwall, 1867)

##### Materials

- **Type status:** Other material. **Occurrence:** sex: 1 female; **Location:** locationID: 4; higherGeography: Macaronesia; continent: Europe; waterBody: Atlantic Ocean; islandGroup: Madeira archipelago; island: Madeira; country: Portugal; countryCode: PT; stateProvince: Madeira; county: Santana; locality: Fajã da Nogueira - Mtdo. do Leacoque; verbatimElevation: 630; decimalLatitude: 32.7415; decimalLongitude: -16.9161; geodeticDatum: WGS84; **Event:** samplingProtocol: Direct sampling
- **Type status:** Other material. **Occurrence:** sex: 1 female; **Location:** locationID: 6; higherGeography: Macaronesia; continent: Europe; waterBody: Atlantic Ocean; islandGroup: Madeira archipelago; island: Madeira; country: Portugal; countryCode: PT; stateProvince: Madeira; county: Santana; locality: Fajã da Nogueira - Mtdo. do Leacoque; verbatimElevation: 614; decimalLatitude: 32.7418; decimalLongitude: -16.9177; geodeticDatum: WGS84; **Event:** samplingProtocol: Direct sampling
- **Type status:** Other material. **Occurrence:** sex: 1 male, 4 females; **Location:** locationID: 11; higherGeography: Macaronesia; continent: Europe; waterBody: Atlantic Ocean; islandGroup: Madeira archipelago; island: Madeira; country: Portugal; countryCode: PT; stateProvince: Madeira; county: Santana; locality: Achada do Teixeira; verbatimElevation: 1211; decimalLatitude: 32.7733; decimalLongitude: -16.9081; geodeticDatum: WGS84; **Event:** samplingProtocol: Direct sampling
- **Type status:** Other material. **Occurrence:** sex: 1 female; **Location:** locationID: 12; higherGeography: Macaronesia; continent: Europe; waterBody: Atlantic Ocean; islandGroup: Madeira archipelago; island: Madeira; country: Portugal; countryCode: PT; stateProvince: Madeira; county: Santana; locality: Achada do Teixeira; verbatimElevation: 1103; decimalLatitude: 32.7762; decimalLongitude: -16.9022; geodeticDatum: WGS84; **Event:** samplingProtocol: Direct sampling
- **Type status:** Other material. **Occurrence:** sex: 1 female; **Location:** locationID: 13; higherGeography: Macaronesia; continent: Europe; waterBody: Atlantic Ocean; islandGroup: Madeira archipelago; island: Madeira; country: Portugal; countryCode: PT; stateProvince: Madeira; county: Santana; locality: Ribeiro Frio - Viveiro; verbatimElevation: 906; decimalLatitude: 32.7354; decimalLongitude: -16.8864; geodeticDatum: WGS84; **Event:** samplingProtocol: Direct sampling
- **Type status:** Other material. **Occurrence:** sex: 1 male; **Location:** locationID: 14; higherGeography: Macaronesia; continent: Europe; waterBody: Atlantic Ocean; islandGroup: Madeira archipelago; island: Madeira; country: Portugal; countryCode: PT; stateProvince: Madeira; county: Santana; locality: Ribeiro Frio - Cottages; verbatimElevation: 994; decimalLatitude: 32.7319; decimalLongitude: -16.8861; geodeticDatum: WGS84; **Event:** samplingProtocol: Direct sampling
- **Type status:** Other material. **Occurrence:** sex: 3 females; **Location:** locationID: 24; higherGeography: Macaronesia; continent: Europe; waterBody: Atlantic Ocean; islandGroup: Madeira archipelago; island: Madeira; country: Portugal; countryCode: PT; stateProvince: Madeira; county: Porto Moniz; locality: Fanal - Levada dos Cedros; verbatimElevation: 820; decimalLatitude: 32.8259; decimalLongitude: -17.158; geodeticDatum: WGS84; **Event:** samplingProtocol: Direct sampling
- **Type status:** Other material. **Occurrence:** sex: 4 females; **Location:** locationID: 25; higherGeography: Macaronesia; continent: Europe; waterBody: Atlantic Ocean; islandGroup: Madeira archipelago; island: Madeira; country: Portugal; countryCode: PT; stateProvince: Madeira; county: Porto Moniz; locality: Fanal; verbatimElevation: 890; decimalLatitude: 32.8236; decimalLongitude: -17.156; geodeticDatum: WGS84; **Event:** samplingProtocol: Direct sampling
- **Type status:** Other material. **Occurrence:** sex: 3 females; **Location:** locationID: 27; higherGeography: Macaronesia; continent: Europe; waterBody: Atlantic Ocean; islandGroup: Madeira archipelago; island: Madeira; country: Portugal; countryCode: PT; stateProvince: Madeira; county: Porto Moniz; locality: Fanal; verbatimElevation: 1023; decimalLatitude: 32.8182; decimalLongitude: -17.1521; geodeticDatum: WGS84; **Event:** samplingProtocol: Direct sampling
- **Type status:** Other material. **Occurrence:** sex: 1 female; **Location:** locationID: 32; higherGeography: Macaronesia; continent: Europe; waterBody: Atlantic Ocean; islandGroup: Madeira archipelago; island: Madeira; country: Portugal; countryCode: PT; stateProvince: Madeira; county: Calheta; locality: Rabaças; verbatimElevation: 993; decimalLatitude: 32.7413; decimalLongitude: -17.0783; geodeticDatum: WGS84; **Event:** samplingProtocol: Pitfall
- **Type status:** Other material. **Occurrence:** sex: 1 female; **Location:** locationID: 33; higherGeography: Macaronesia; continent: Europe; waterBody: Atlantic Ocean; islandGroup: Madeira archipelago; island: Madeira; country: Portugal; countryCode: PT; stateProvince: Madeira; county: Porto Moniz; locality: Rabaçal; verbatimElevation: 930; decimalLatitude: 32.7647; decimalLongitude: -17.1341; geodeticDatum: WGS84; **Event:** samplingProtocol: Direct sampling
- **Type status:** Other material. **Occurrence:** sex: 1 female; **Location:** locationID: 34; higherGeography: Macaronesia; continent: Europe; waterBody: Atlantic Ocean; islandGroup: Madeira archipelago; island: Madeira; country: Portugal; countryCode: PT; stateProvince: Madeira; county: Ponta do Sol; locality: Risco; verbatimElevation: 1048; decimalLatitude: 32.7608; decimalLongitude: -17.1256; geodeticDatum: WGS84; **Event:** samplingProtocol: Direct sampling

##### Ecological interactions

###### Native status

macaronesian endemic

##### Distribution

Madeira archipelago, Azores

##### Notes

This Macaronesian endemic occurs both in Madeira and Porto Santo islands. In the Azores, it was reported from locations at low altitude, being very common in the canopies of orchards and exotic trees.

#### Macaroeris sp.

##### Materials

- **Type status:** Other material. **Occurrence:** sex: 1 female; **Location:** locationID: 2; higherGeography: Macaronesia; continent: Europe; waterBody: Atlantic Ocean; islandGroup: Madeira archipelago; island: Madeira; country: Portugal; countryCode: PT; stateProvince: Madeira; county: Machico; locality: Funduras; verbatimElevation: 552; decimalLatitude: 32.754; decimalLongitude: -16.8099; geodeticDatum: WGS84; **Event:** samplingProtocol: Pitfall
- **Type status:** Other material. **Occurrence:** sex: 1 female; **Location:** locationID: 17; higherGeography: Macaronesia; continent: Europe; waterBody: Atlantic Ocean; islandGroup: Madeira archipelago; island: Madeira; country: Portugal; countryCode: PT; stateProvince: Madeira; county: Porto Moniz; locality: Chão da Ribeira; verbatimElevation: 519; decimalLatitude: 32.7933; decimalLongitude: -17.1122; geodeticDatum: WGS84; **Event:** samplingProtocol: Direct sampling
- **Type status:** Other material. **Occurrence:** sex: 2 males, 1 female; **Location:** locationID: 32; higherGeography: Macaronesia; continent: Europe; waterBody: Atlantic Ocean; islandGroup: Madeira archipelago; island: Madeira; country: Portugal; countryCode: PT; stateProvince: Madeira; county: Calheta; locality: Rabaças; verbatimElevation: 993; decimalLatitude: 32.7413; decimalLongitude: -17.0783; geodeticDatum: WGS84; **Event:** samplingProtocol: Direct sampling

##### Ecological interactions

###### Native status

native

##### Notes

The taxonomy of the genus *Macaroeris* Wunderlich, 1992 is unclear, with some species still poorly described. Several specimens of this species were identified as different from *Macaroeris diligens*, but their species affiliation was not possible.

#### Pellenes maderianus

Kulczynski, 1905

##### Materials

- **Type status:** Other material. **Occurrence:** sex: 1 male; **Location:** locationID: 15; higherGeography: Macaronesia; continent: Europe; waterBody: Atlantic Ocean; islandGroup: Madeira archipelago; island: Madeira; country: Portugal; countryCode: PT; stateProvince: Madeira; county: Santana; locality: Pico do Areeiro; verbatimElevation: 1533; decimalLatitude: 32.7231; decimalLongitude: -16.9109; geodeticDatum: WGS84; **Event:** samplingProtocol: Pitfall
- **Type status:** Other material. **Occurrence:** sex: 1 male; **Location:** locationID: 32; higherGeography: Macaronesia; continent: Europe; waterBody: Atlantic Ocean; islandGroup: Madeira archipelago; island: Madeira; country: Portugal; countryCode: PT; stateProvince: Madeira; county: Calheta; locality: Rabaças; verbatimElevation: 993; decimalLatitude: 32.7413; decimalLongitude: -17.0783; geodeticDatum: WGS84; **Event:** samplingProtocol: Pitfall

##### Ecological interactions

###### Native status

native

##### Distribution

Madeira island, Israel

##### Notes

Some confusion appears to exist regarding the identity of *Pellenes maderianus*. Although most authors consider this species a valid taxon, some support that it is a synonymy of *Pellenes epularis* (O. Pickard-Cambridge, 1872) (Logunov et al. 1999).

#### Meta stridulans

Wunderlich, 1992

##### Materials

- **Type status:** Other material. **Occurrence:** sex: 2 females; **Location:** locationID: 17; higherGeography: Macaronesia; continent: Europe; waterBody: Atlantic Ocean; islandGroup: Madeira archipelago; island: Madeira; country: Portugal; countryCode: PT; stateProvince: Madeira; county: Porto Moniz; locality: Chão da Ribeira; verbatimElevation: 519; decimalLatitude: 32.7933; decimalLongitude: -17.1122; geodeticDatum: WGS84; **Event:** samplingProtocol: Direct sampling
- **Type status:** Other material. **Occurrence:** sex: 1 male; **Location:** locationID: 18; higherGeography: Macaronesia; continent: Europe; waterBody: Atlantic Ocean; islandGroup: Madeira archipelago; island: Madeira; country: Portugal; countryCode: PT; stateProvince: Madeira; county: Porto Moniz; locality: Chão da Ribeira; verbatimElevation: 491; decimalLatitude: 32.7957; decimalLongitude: -17.1117; geodeticDatum: WGS84; **Event:** samplingProtocol: Direct sampling
- **Type status:** Other material. **Occurrence:** sex: 1 female; **Location:** locationID: 25; higherGeography: Macaronesia; continent: Europe; waterBody: Atlantic Ocean; islandGroup: Madeira archipelago; island: Madeira; country: Portugal; countryCode: PT; stateProvince: Madeira; county: Porto Moniz; locality: Fanal; verbatimElevation: 890; decimalLatitude: 32.8236; decimalLongitude: -17.156; geodeticDatum: WGS84; **Event:** samplingProtocol: Direct sampling

##### Ecological interactions

###### Native status

SIE

##### Distribution

Madeira island (Fig. 4b)

##### Notes

Previous records were mostly from Laurisilva with a single record from a moutain area (Pico do Areeiro). Our records confirm the association of this endemic species with Laurisilva.

#### Tetragnatha intermedia

Kulczynski, 1891

##### Materials

- **Type status:** Other material. **Occurrence:** sex: 1 female; **Location:** locationID: 2; higherGeography: Macaronesia; continent: Europe; waterBody: Atlantic Ocean; islandGroup: Madeira archipelago; island: Madeira; country: Portugal; countryCode: PT; stateProvince: Madeira; county: Machico; locality: Funduras; verbatimElevation: 552; decimalLatitude: 32.754; decimalLongitude: -16.8099; geodeticDatum: WGS84; **Event:** samplingProtocol: Direct sampling

##### Ecological interactions

###### Native status

introduced

##### Distribution

Portugal to Hungary, Russia, Madeira

##### Notes

First record for Madeira island. It is possible that previous records of *Tetragnatha obtusa* C. L. Koch, 1837 from the Madeira archipelago (Cardoso and Crespo 2008) refer to *Tetragnatha intermedia* as the former was believed to be a subspecies of the latter until the elevation of *Tetragnatha intermedia* to species level (Wunderlich 2011).

#### Cryptachaea blattea

(Urquhart, 1886)

##### Materials

- **Type status:** Other material. **Occurrence:** sex: 1 male, 2 females; **Location:** locationID: 1; higherGeography: Macaronesia; continent: Europe; waterBody: Atlantic Ocean; islandGroup: Madeira archipelago; island: Madeira; country: Portugal; countryCode: PT; stateProvince: Madeira; county: Machico; locality: Funduras; verbatimElevation: 500; decimalLatitude: 32.7493; decimalLongitude: -16.8114; geodeticDatum: WGS84; **Event:** samplingProtocol: Direct sampling
- **Type status:** Other material. **Occurrence:** sex: 4 females; **Location:** locationID: 2; higherGeography: Macaronesia; continent: Europe; waterBody: Atlantic Ocean; islandGroup: Madeira archipelago; island: Madeira; country: Portugal; countryCode: PT; stateProvince: Madeira; county: Machico; locality: Funduras; verbatimElevation: 552; decimalLatitude: 32.754; decimalLongitude: -16.8099; geodeticDatum: WGS84; **Event:** samplingProtocol: Direct sampling
- **Type status:** Other material. **Occurrence:** sex: 2 males, 2 females; **Location:** locationID: 2; higherGeography: Macaronesia; continent: Europe; waterBody: Atlantic Ocean; islandGroup: Madeira archipelago; island: Madeira; country: Portugal; countryCode: PT; stateProvince: Madeira; county: Machico; locality: Funduras; verbatimElevation: 552; decimalLatitude: 32.754; decimalLongitude: -16.8099; geodeticDatum: WGS84; **Event:** samplingProtocol: Pitfall
- **Type status:** Other material. **Occurrence:** sex: 1 male, 6 females; **Location:** locationID: 4; higherGeography: Macaronesia; continent: Europe; waterBody: Atlantic Ocean; islandGroup: Madeira archipelago; island: Madeira; country: Portugal; countryCode: PT; stateProvince: Madeira; county: Santana; locality: Fajã da Nogueira - Mtdo. do Leacoque; verbatimElevation: 630; decimalLatitude: 32.7415; decimalLongitude: -16.9161; geodeticDatum: WGS84; **Event:** samplingProtocol: Direct sampling
- **Type status:** Other material. **Occurrence:** sex: 2 females; **Location:** locationID: 4; higherGeography: Macaronesia; continent: Europe; waterBody: Atlantic Ocean; islandGroup: Madeira archipelago; island: Madeira; country: Portugal; countryCode: PT; stateProvince: Madeira; county: Santana; locality: Fajã da Nogueira - Mtdo. do Leacoque; verbatimElevation: 630; decimalLatitude: 32.7415; decimalLongitude: -16.9161; geodeticDatum: WGS84; **Event:** samplingProtocol: Pitfall
- **Type status:** Other material. **Occurrence:** sex: 1 female; **Location:** locationID: 6; higherGeography: Macaronesia; continent: Europe; waterBody: Atlantic Ocean; islandGroup: Madeira archipelago; island: Madeira; country: Portugal; countryCode: PT; stateProvince: Madeira; county: Santana; locality: Fajã da Nogueira - Mtdo. do Leacoque; verbatimElevation: 614; decimalLatitude: 32.7418; decimalLongitude: -16.9177; geodeticDatum: WGS84; **Event:** samplingProtocol: Direct sampling
- **Type status:** Other material. **Occurrence:** sex: 1 female; **Location:** locationID: 6; higherGeography: Macaronesia; continent: Europe; waterBody: Atlantic Ocean; islandGroup: Madeira archipelago; island: Madeira; country: Portugal; countryCode: PT; stateProvince: Madeira; county: Santana; locality: Fajã da Nogueira - Mtdo. do Leacoque; verbatimElevation: 614; decimalLatitude: 32.7418; decimalLongitude: -16.9177; geodeticDatum: WGS84; **Event:** samplingProtocol: Pitfall
- **Type status:** Other material. **Occurrence:** sex: 2 males; **Location:** locationID: 7; higherGeography: Macaronesia; continent: Europe; waterBody: Atlantic Ocean; islandGroup: Madeira archipelago; island: Madeira; country: Portugal; countryCode: PT; stateProvince: Madeira; county: Santana; locality: Fajã da Nogueira - Tanque; verbatimElevation: 845; decimalLatitude: 32.7425; decimalLongitude: -16.9168; geodeticDatum: WGS84; **Event:** samplingProtocol: Direct sampling
- **Type status:** Other material. **Occurrence:** sex: 1 female; **Location:** locationID: 8; higherGeography: Macaronesia; continent: Europe; waterBody: Atlantic Ocean; islandGroup: Madeira archipelago; island: Madeira; country: Portugal; countryCode: PT; stateProvince: Madeira; county: Santana; locality: Fajã da Nogueira - Til Gigante; verbatimElevation: 841; decimalLatitude: 32.7457; decimalLongitude: -16.915; geodeticDatum: WGS84; **Event:** samplingProtocol: Pitfall
- **Type status:** Other material. **Occurrence:** sex: 2 females; **Location:** locationID: 9; higherGeography: Macaronesia; continent: Europe; waterBody: Atlantic Ocean; islandGroup: Madeira archipelago; island: Madeira; country: Portugal; countryCode: PT; stateProvince: Madeira; county: Santana; locality: Queimadas; verbatimElevation: 841; decimalLatitude: 32.7873; decimalLongitude: -16.9047; geodeticDatum: WGS84; **Event:** samplingProtocol: Direct sampling
- **Type status:** Other material. **Occurrence:** sex: 1 male, 4 females; **Location:** locationID: 10; higherGeography: Macaronesia; continent: Europe; waterBody: Atlantic Ocean; islandGroup: Madeira archipelago; island: Madeira; country: Portugal; countryCode: PT; stateProvince: Madeira; county: Santana; locality: Pico das Pedras; verbatimElevation: 883; decimalLatitude: 32.7841; decimalLongitude: -16.9055; geodeticDatum: WGS84; **Event:** samplingProtocol: Direct sampling
- **Type status:** Other material. **Occurrence:** sex: 4 males, 5 females; **Location:** locationID: 11; higherGeography: Macaronesia; continent: Europe; waterBody: Atlantic Ocean; islandGroup: Madeira archipelago; island: Madeira; country: Portugal; countryCode: PT; stateProvince: Madeira; county: Santana; locality: Achada do Teixeira; verbatimElevation: 1211; decimalLatitude: 32.7733; decimalLongitude: -16.9081; geodeticDatum: WGS84; **Event:** samplingProtocol: Direct sampling
- **Type status:** Other material. **Occurrence:** sex: 1 female; **Location:** locationID: 11; higherGeography: Macaronesia; continent: Europe; waterBody: Atlantic Ocean; islandGroup: Madeira archipelago; island: Madeira; country: Portugal; countryCode: PT; stateProvince: Madeira; county: Santana; locality: Achada do Teixeira; verbatimElevation: 1211; decimalLatitude: 32.7733; decimalLongitude: -16.9081; geodeticDatum: WGS84; **Event:** samplingProtocol: Pitfall
- **Type status:** Other material. **Occurrence:** sex: 2 males, 1 female; **Location:** locationID: 12; higherGeography: Macaronesia; continent: Europe; waterBody: Atlantic Ocean; islandGroup: Madeira archipelago; island: Madeira; country: Portugal; countryCode: PT; stateProvince: Madeira; county: Santana; locality: Achada do Teixeira; verbatimElevation: 1103; decimalLatitude: 32.7762; decimalLongitude: -16.9022; geodeticDatum: WGS84; **Event:** samplingProtocol: Direct sampling
- **Type status:** Other material. **Occurrence:** sex: 2 females; **Location:** locationID: 12; higherGeography: Macaronesia; continent: Europe; waterBody: Atlantic Ocean; islandGroup: Madeira archipelago; island: Madeira; country: Portugal; countryCode: PT; stateProvince: Madeira; county: Santana; locality: Achada do Teixeira; verbatimElevation: 1103; decimalLatitude: 32.7762; decimalLongitude: -16.9022; geodeticDatum: WGS84; **Event:** samplingProtocol: Pitfall
- **Type status:** Other material. **Occurrence:** sex: 2 males; **Location:** locationID: 13; higherGeography: Macaronesia; continent: Europe; waterBody: Atlantic Ocean; islandGroup: Madeira archipelago; island: Madeira; country: Portugal; countryCode: PT; stateProvince: Madeira; county: Santana; locality: Ribeiro Frio - Viveiro; verbatimElevation: 906; decimalLatitude: 32.7354; decimalLongitude: -16.8864; geodeticDatum: WGS84; **Event:** samplingProtocol: Direct sampling
- **Type status:** Other material. **Occurrence:** sex: 2 females; **Location:** locationID: 14; higherGeography: Macaronesia; continent: Europe; waterBody: Atlantic Ocean; islandGroup: Madeira archipelago; island: Madeira; country: Portugal; countryCode: PT; stateProvince: Madeira; county: Santana; locality: Ribeiro Frio - Cottages; verbatimElevation: 994; decimalLatitude: 32.7319; decimalLongitude: -16.8861; geodeticDatum: WGS84; **Event:** samplingProtocol: Direct sampling
- **Type status:** Other material. **Occurrence:** sex: 1 female; **Location:** locationID: 15; higherGeography: Macaronesia; continent: Europe; waterBody: Atlantic Ocean; islandGroup: Madeira archipelago; island: Madeira; country: Portugal; countryCode: PT; stateProvince: Madeira; county: Santana; locality: Pico do Areeiro; verbatimElevation: 1533; decimalLatitude: 32.7231; decimalLongitude: -16.9109; geodeticDatum: WGS84; **Event:** samplingProtocol: Direct sampling
- **Type status:** Other material. **Occurrence:** sex: 1 male; **Location:** locationID: 17; higherGeography: Macaronesia; continent: Europe; waterBody: Atlantic Ocean; islandGroup: Madeira archipelago; island: Madeira; country: Portugal; countryCode: PT; stateProvince: Madeira; county: Porto Moniz; locality: Chão da Ribeira; verbatimElevation: 519; decimalLatitude: 32.7933; decimalLongitude: -17.1122; geodeticDatum: WGS84; **Event:** samplingProtocol: Pitfall
- **Type status:** Other material. **Occurrence:** sex: 3 females; **Location:** locationID: 18; higherGeography: Macaronesia; continent: Europe; waterBody: Atlantic Ocean; islandGroup: Madeira archipelago; island: Madeira; country: Portugal; countryCode: PT; stateProvince: Madeira; county: Porto Moniz; locality: Chão da Ribeira; verbatimElevation: 491; decimalLatitude: 32.7957; decimalLongitude: -17.1117; geodeticDatum: WGS84; **Event:** samplingProtocol: Direct sampling
- **Type status:** Other material. **Occurrence:** sex: 2 males, 4 females; **Location:** locationID: 20; higherGeography: Macaronesia; continent: Europe; waterBody: Atlantic Ocean; islandGroup: Madeira archipelago; island: Madeira; country: Portugal; countryCode: PT; stateProvince: Madeira; county: São Vicente; locality: Encumeada; verbatimElevation: 999; decimalLatitude: 32.7558; decimalLongitude: -17.0143; geodeticDatum: WGS84; **Event:** samplingProtocol: Pitfall
- **Type status:** Other material. **Occurrence:** sex: 5 females; **Location:** locationID: 21; higherGeography: Macaronesia; continent: Europe; waterBody: Atlantic Ocean; islandGroup: Madeira archipelago; island: Madeira; country: Portugal; countryCode: PT; stateProvince: Madeira; county: Santana; locality: Ribeiro Bonito - Levada; verbatimElevation: 568; decimalLatitude: 32.8047; decimalLongitude: -16.9346; geodeticDatum: WGS84; **Event:** samplingProtocol: Direct sampling
- **Type status:** Other material. **Occurrence:** sex: 1 female; **Location:** locationID: 21; higherGeography: Macaronesia; continent: Europe; waterBody: Atlantic Ocean; islandGroup: Madeira archipelago; island: Madeira; country: Portugal; countryCode: PT; stateProvince: Madeira; county: Santana; locality: Ribeiro Bonito - Levada; verbatimElevation: 568; decimalLatitude: 32.8047; decimalLongitude: -16.9346; geodeticDatum: WGS84; **Event:** samplingProtocol: Pitfall
- **Type status:** Other material. **Occurrence:** sex: 1 female; **Location:** locationID: 22; higherGeography: Macaronesia; continent: Europe; waterBody: Atlantic Ocean; islandGroup: Madeira archipelago; island: Madeira; country: Portugal; countryCode: PT; stateProvince: Madeira; county: Santana; locality: Ribeiro Bonito - Ribeiro; verbatimElevation: 560; decimalLatitude: 32.7985; decimalLongitude: -16.936; geodeticDatum: WGS84; **Event:** samplingProtocol: Direct sampling
- **Type status:** Other material. **Occurrence:** sex: 1 female; **Location:** locationID: 22; higherGeography: Macaronesia; continent: Europe; waterBody: Atlantic Ocean; islandGroup: Madeira archipelago; island: Madeira; country: Portugal; countryCode: PT; stateProvince: Madeira; county: Santana; locality: Ribeiro Bonito - Ribeiro; verbatimElevation: 560; decimalLatitude: 32.7985; decimalLongitude: -16.936; geodeticDatum: WGS84; **Event:** samplingProtocol: Pitfall
- **Type status:** Other material. **Occurrence:** sex: 3 females; **Location:** locationID: 23; higherGeography: Macaronesia; continent: Europe; waterBody: Atlantic Ocean; islandGroup: Madeira archipelago; island: Madeira; country: Portugal; countryCode: PT; stateProvince: Madeira; county: Porto Moniz; locality: Fanal; verbatimElevation: 755; decimalLatitude: 32.8302; decimalLongitude: -17.1585; geodeticDatum: WGS84; **Event:** samplingProtocol: Pitfall
- **Type status:** Other material. **Occurrence:** sex: 3 males, 4 females; **Location:** locationID: 24; higherGeography: Macaronesia; continent: Europe; waterBody: Atlantic Ocean; islandGroup: Madeira archipelago; island: Madeira; country: Portugal; countryCode: PT; stateProvince: Madeira; county: Porto Moniz; locality: Fanal - Levada dos Cedros; verbatimElevation: 820; decimalLatitude: 32.8259; decimalLongitude: -17.158; geodeticDatum: WGS84; **Event:** samplingProtocol: Pitfall
- **Type status:** Other material. **Occurrence:** sex: 2 females; **Location:** locationID: 25; higherGeography: Macaronesia; continent: Europe; waterBody: Atlantic Ocean; islandGroup: Madeira archipelago; island: Madeira; country: Portugal; countryCode: PT; stateProvince: Madeira; county: Porto Moniz; locality: Fanal; verbatimElevation: 890; decimalLatitude: 32.8236; decimalLongitude: -17.156; geodeticDatum: WGS84; **Event:** samplingProtocol: Direct sampling
- **Type status:** Other material. **Occurrence:** sex: 1 male, 5 females; **Location:** locationID: 25; higherGeography: Macaronesia; continent: Europe; waterBody: Atlantic Ocean; islandGroup: Madeira archipelago; island: Madeira; country: Portugal; countryCode: PT; stateProvince: Madeira; county: Porto Moniz; locality: Fanal; verbatimElevation: 890; decimalLatitude: 32.8236; decimalLongitude: -17.156; geodeticDatum: WGS84; **Event:** samplingProtocol: Pitfall
- **Type status:** Other material. **Occurrence:** sex: 1 male, 2 females; **Location:** locationID: 26; higherGeography: Macaronesia; continent: Europe; waterBody: Atlantic Ocean; islandGroup: Madeira archipelago; island: Madeira; country: Portugal; countryCode: PT; stateProvince: Madeira; county: Porto Moniz; locality: Fanal; verbatimElevation: 889; decimalLatitude: 32.8226; decimalLongitude: -17.1539; geodeticDatum: WGS84; **Event:** samplingProtocol: Pitfall
- **Type status:** Other material. **Occurrence:** sex: 2 females; **Location:** locationID: 27; higherGeography: Macaronesia; continent: Europe; waterBody: Atlantic Ocean; islandGroup: Madeira archipelago; island: Madeira; country: Portugal; countryCode: PT; stateProvince: Madeira; county: Porto Moniz; locality: Fanal; verbatimElevation: 1023; decimalLatitude: 32.8182; decimalLongitude: -17.1521; geodeticDatum: WGS84; **Event:** samplingProtocol: Direct sampling
- **Type status:** Other material. **Occurrence:** sex: 1 female; **Location:** locationID: 27; higherGeography: Macaronesia; continent: Europe; waterBody: Atlantic Ocean; islandGroup: Madeira archipelago; island: Madeira; country: Portugal; countryCode: PT; stateProvince: Madeira; county: Porto Moniz; locality: Fanal; verbatimElevation: 1023; decimalLatitude: 32.8182; decimalLongitude: -17.1521; geodeticDatum: WGS84; **Event:** samplingProtocol: Pitfall
- **Type status:** Other material. **Occurrence:** sex: 1 male, 3 females; **Location:** locationID: 28; higherGeography: Macaronesia; continent: Europe; waterBody: Atlantic Ocean; islandGroup: Madeira archipelago; island: Madeira; country: Portugal; countryCode: PT; stateProvince: Madeira; county: Porto Moniz; locality: Fanal; verbatimElevation: 1134; decimalLatitude: 32.8062; decimalLongitude: -17.1409; geodeticDatum: WGS84; **Event:** samplingProtocol: Direct sampling
- **Type status:** Other material. **Occurrence:** sex: 2 females; **Location:** locationID: 28; higherGeography: Macaronesia; continent: Europe; waterBody: Atlantic Ocean; islandGroup: Madeira archipelago; island: Madeira; country: Portugal; countryCode: PT; stateProvince: Madeira; county: Porto Moniz; locality: Fanal; verbatimElevation: 1134; decimalLatitude: 32.8062; decimalLongitude: -17.1409; geodeticDatum: WGS84; **Event:** samplingProtocol: Pitfall
- **Type status:** Other material. **Occurrence:** sex: 1 male; **Location:** locationID: 29; higherGeography: Macaronesia; continent: Europe; waterBody: Atlantic Ocean; islandGroup: Madeira archipelago; island: Madeira; country: Portugal; countryCode: PT; stateProvince: Madeira; county: São Vicente; locality: Ginjas; verbatimElevation: 869; decimalLatitude: 32.7758; decimalLongitude: -17.0534; geodeticDatum: WGS84; **Event:** samplingProtocol: Direct sampling
- **Type status:** Other material. **Occurrence:** sex: 1 female; **Location:** locationID: 29; higherGeography: Macaronesia; continent: Europe; waterBody: Atlantic Ocean; islandGroup: Madeira archipelago; island: Madeira; country: Portugal; countryCode: PT; stateProvince: Madeira; county: São Vicente; locality: Ginjas; verbatimElevation: 869; decimalLatitude: 32.7758; decimalLongitude: -17.0534; geodeticDatum: WGS84; **Event:** samplingProtocol: Pitfall
- **Type status:** Other material. **Occurrence:** sex: 1 female; **Location:** locationID: 33; higherGeography: Macaronesia; continent: Europe; waterBody: Atlantic Ocean; islandGroup: Madeira archipelago; island: Madeira; country: Portugal; countryCode: PT; stateProvince: Madeira; county: Porto Moniz; locality: Rabaçal; verbatimElevation: 930; decimalLatitude: 32.7647; decimalLongitude: -17.1341; geodeticDatum: WGS84; **Event:** samplingProtocol: Direct sampling
- **Type status:** Other material. **Occurrence:** sex: 4 females; **Location:** locationID: 33; higherGeography: Macaronesia; continent: Europe; waterBody: Atlantic Ocean; islandGroup: Madeira archipelago; island: Madeira; country: Portugal; countryCode: PT; stateProvince: Madeira; county: Porto Moniz; locality: Rabaçal; verbatimElevation: 930; decimalLatitude: 32.7647; decimalLongitude: -17.1341; geodeticDatum: WGS84; **Event:** samplingProtocol: Pitfall
- **Type status:** Other material. **Occurrence:** sex: 1 male, 1 female; **Location:** locationID: 35; higherGeography: Macaronesia; continent: Europe; waterBody: Atlantic Ocean; islandGroup: Madeira archipelago; island: Madeira; country: Portugal; countryCode: PT; stateProvince: Madeira; county: Porto Moniz; locality: Casa do Elias; verbatimElevation: 814; decimalLatitude: 32.8268; decimalLongitude: -17.1883; geodeticDatum: WGS84; **Event:** samplingProtocol: Pitfall

##### Ecological interactions

###### Native status

introduced

##### Distribution

Cosmopolitan

#### Dipoenata cf. longitarsis

(Denis, 1962)

##### Materials

- **Type status:** Other material. **Occurrence:** sex: 1 male; **Location:** locationID: 7; higherGeography: Macaronesia; continent: Europe; waterBody: Atlantic Ocean; islandGroup: Madeira archipelago; island: Madeira; country: Portugal; countryCode: PT; stateProvince: Madeira; county: Santana; locality: Fajã da Nogueira - Tanque; verbatimElevation: 845; decimalLatitude: 32.7425; decimalLongitude: -16.9168; geodeticDatum: WGS84; **Event:** samplingProtocol: Direct sampling

##### Ecological interactions

###### Native status

SIE

##### Distribution

Madeira island (Fig. 4c)

##### Notes

The male of *Dipoenata longitarsis* is unknown. This preliminary identification is based on the observation of somatical similarities between a male and a female hadrotarsine also collected in Laurisilva and identified as *Dipoenata longitarsis*.

#### Enoplognatha sattleri

Bösenberg, 1895

##### Materials

- **Type status:** Other material. **Occurrence:** sex: 2 females; **Location:** locationID: 11; higherGeography: Macaronesia; continent: Europe; waterBody: Atlantic Ocean; islandGroup: Madeira archipelago; island: Madeira; country: Portugal; countryCode: PT; stateProvince: Madeira; county: Santana; locality: Achada do Teixeira; verbatimElevation: 1211; decimalLatitude: 32.7733; decimalLongitude: -16.9081; geodeticDatum: WGS84; **Event:** samplingProtocol: Direct sampling
- **Type status:** Other material. **Occurrence:** sex: 1 female; **Location:** locationID: 21; higherGeography: Macaronesia; continent: Europe; waterBody: Atlantic Ocean; islandGroup: Madeira archipelago; island: Madeira; country: Portugal; countryCode: PT; stateProvince: Madeira; county: Santana; locality: Ribeiro Bonito - Levada; verbatimElevation: 568; decimalLatitude: 32.8047; decimalLongitude: -16.9346; geodeticDatum: WGS84; **Event:** samplingProtocol: Direct sampling
- **Type status:** Other material. **Occurrence:** sex: 1 female; **Location:** locationID: 25; higherGeography: Macaronesia; continent: Europe; waterBody: Atlantic Ocean; islandGroup: Madeira archipelago; island: Madeira; country: Portugal; countryCode: PT; stateProvince: Madeira; county: Porto Moniz; locality: Fanal; verbatimElevation: 890; decimalLatitude: 32.8236; decimalLongitude: -17.156; geodeticDatum: WGS84; **Event:** samplingProtocol: Direct sampling

##### Ecological interactions

###### Native status

native

##### Distribution

Madeira island, Selvagens, Canary Islands

#### Episinus maderianus

Kulczynski, 1905

##### Materials

- **Type status:** Other material. **Occurrence:** sex: 1 male, 1 female; **Location:** locationID: 1; higherGeography: Macaronesia; continent: Europe; waterBody: Atlantic Ocean; islandGroup: Madeira archipelago; island: Madeira; country: Portugal; countryCode: PT; stateProvince: Madeira; county: Machico; locality: Funduras; verbatimElevation: 500; decimalLatitude: 32.7493; decimalLongitude: -16.8114; geodeticDatum: WGS84; **Event:** samplingProtocol: Direct sampling
- **Type status:** Other material. **Occurrence:** sex: 1 male, 1 female; **Location:** locationID: 4; higherGeography: Macaronesia; continent: Europe; waterBody: Atlantic Ocean; islandGroup: Madeira archipelago; island: Madeira; country: Portugal; countryCode: PT; stateProvince: Madeira; county: Santana; locality: Fajã da Nogueira - Mtdo. do Leacoque; verbatimElevation: 630; decimalLatitude: 32.7415; decimalLongitude: -16.9161; geodeticDatum: WGS84; **Event:** samplingProtocol: Direct sampling
- **Type status:** Other material. **Occurrence:** sex: 1 female; **Location:** locationID: 7; higherGeography: Macaronesia; continent: Europe; waterBody: Atlantic Ocean; islandGroup: Madeira archipelago; island: Madeira; country: Portugal; countryCode: PT; stateProvince: Madeira; county: Santana; locality: Fajã da Nogueira - Tanque; verbatimElevation: 845; decimalLatitude: 32.7425; decimalLongitude: -16.9168; geodeticDatum: WGS84; **Event:** samplingProtocol: Direct sampling
- **Type status:** Other material. **Occurrence:** sex: 1 male; **Location:** locationID: 9; higherGeography: Macaronesia; continent: Europe; waterBody: Atlantic Ocean; islandGroup: Madeira archipelago; island: Madeira; country: Portugal; countryCode: PT; stateProvince: Madeira; county: Santana; locality: Queimadas; verbatimElevation: 841; decimalLatitude: 32.7873; decimalLongitude: -16.9047; geodeticDatum: WGS84; **Event:** samplingProtocol: Direct sampling
- **Type status:** Other material. **Occurrence:** sex: 2 males; **Location:** locationID: 10; higherGeography: Macaronesia; continent: Europe; waterBody: Atlantic Ocean; islandGroup: Madeira archipelago; island: Madeira; country: Portugal; countryCode: PT; stateProvince: Madeira; county: Santana; locality: Pico das Pedras; verbatimElevation: 883; decimalLatitude: 32.7841; decimalLongitude: -16.9055; geodeticDatum: WGS84; **Event:** samplingProtocol: Direct sampling
- **Type status:** Other material. **Occurrence:** sex: 1 male; **Location:** locationID: 12; higherGeography: Macaronesia; continent: Europe; waterBody: Atlantic Ocean; islandGroup: Madeira archipelago; island: Madeira; country: Portugal; countryCode: PT; stateProvince: Madeira; county: Santana; locality: Achada do Teixeira; verbatimElevation: 1103; decimalLatitude: 32.7762; decimalLongitude: -16.9022; geodeticDatum: WGS84; **Event:** samplingProtocol: Direct sampling
- **Type status:** Other material. **Occurrence:** sex: 2 males; **Location:** locationID: 13; higherGeography: Macaronesia; continent: Europe; waterBody: Atlantic Ocean; islandGroup: Madeira archipelago; island: Madeira; country: Portugal; countryCode: PT; stateProvince: Madeira; county: Santana; locality: Ribeiro Frio - Viveiro; verbatimElevation: 906; decimalLatitude: 32.7354; decimalLongitude: -16.8864; geodeticDatum: WGS84; **Event:** samplingProtocol: Direct sampling
- **Type status:** Other material. **Occurrence:** sex: 1 male; **Location:** locationID: 14; higherGeography: Macaronesia; continent: Europe; waterBody: Atlantic Ocean; islandGroup: Madeira archipelago; island: Madeira; country: Portugal; countryCode: PT; stateProvince: Madeira; county: Santana; locality: Ribeiro Frio - Cottages; verbatimElevation: 994; decimalLatitude: 32.7319; decimalLongitude: -16.8861; geodeticDatum: WGS84; **Event:** samplingProtocol: Direct sampling
- **Type status:** Other material. **Occurrence:** sex: 1 female; **Location:** locationID: 17; higherGeography: Macaronesia; continent: Europe; waterBody: Atlantic Ocean; islandGroup: Madeira archipelago; island: Madeira; country: Portugal; countryCode: PT; stateProvince: Madeira; county: Porto Moniz; locality: Chão da Ribeira; verbatimElevation: 519; decimalLatitude: 32.7933; decimalLongitude: -17.1122; geodeticDatum: WGS84; **Event:** samplingProtocol: Direct sampling
- **Type status:** Other material. **Occurrence:** sex: 1 female; **Location:** locationID: 18; higherGeography: Macaronesia; continent: Europe; waterBody: Atlantic Ocean; islandGroup: Madeira archipelago; island: Madeira; country: Portugal; countryCode: PT; stateProvince: Madeira; county: Porto Moniz; locality: Chão da Ribeira; verbatimElevation: 491; decimalLatitude: 32.7957; decimalLongitude: -17.1117; geodeticDatum: WGS84; **Event:** samplingProtocol: Direct sampling
- **Type status:** Other material. **Occurrence:** sex: 1 male, 4 females; **Location:** locationID: 21; higherGeography: Macaronesia; continent: Europe; waterBody: Atlantic Ocean; islandGroup: Madeira archipelago; island: Madeira; country: Portugal; countryCode: PT; stateProvince: Madeira; county: Santana; locality: Ribeiro Bonito - Levada; verbatimElevation: 568; decimalLatitude: 32.8047; decimalLongitude: -16.9346; geodeticDatum: WGS84; **Event:** samplingProtocol: Direct sampling
- **Type status:** Other material. **Occurrence:** sex: 1 male, 1 female; **Location:** locationID: 22; higherGeography: Macaronesia; continent: Europe; waterBody: Atlantic Ocean; islandGroup: Madeira archipelago; island: Madeira; country: Portugal; countryCode: PT; stateProvince: Madeira; county: Santana; locality: Ribeiro Bonito - Ribeiro; verbatimElevation: 560; decimalLatitude: 32.7985; decimalLongitude: -16.936; geodeticDatum: WGS84; **Event:** samplingProtocol: Direct sampling
- **Type status:** Other material. **Occurrence:** sex: 1 female; **Location:** locationID: 25; higherGeography: Macaronesia; continent: Europe; waterBody: Atlantic Ocean; islandGroup: Madeira archipelago; island: Madeira; country: Portugal; countryCode: PT; stateProvince: Madeira; county: Porto Moniz; locality: Fanal; verbatimElevation: 890; decimalLatitude: 32.8236; decimalLongitude: -17.156; geodeticDatum: WGS84; **Event:** samplingProtocol: Direct sampling
- **Type status:** Other material. **Occurrence:** sex: 1 male, 1 female; **Location:** locationID: 27; higherGeography: Macaronesia; continent: Europe; waterBody: Atlantic Ocean; islandGroup: Madeira archipelago; island: Madeira; country: Portugal; countryCode: PT; stateProvince: Madeira; county: Porto Moniz; locality: Fanal; verbatimElevation: 1023; decimalLatitude: 32.8182; decimalLongitude: -17.1521; geodeticDatum: WGS84; **Event:** samplingProtocol: Direct sampling
- **Type status:** Other material. **Occurrence:** sex: 1 female; **Location:** locationID: 28; higherGeography: Macaronesia; continent: Europe; waterBody: Atlantic Ocean; islandGroup: Madeira archipelago; island: Madeira; country: Portugal; countryCode: PT; stateProvince: Madeira; county: Porto Moniz; locality: Fanal; verbatimElevation: 1134; decimalLatitude: 32.8062; decimalLongitude: -17.1409; geodeticDatum: WGS84; **Event:** samplingProtocol: Direct sampling
- **Type status:** Other material. **Occurrence:** sex: 1 male; **Location:** locationID: 33; higherGeography: Macaronesia; continent: Europe; waterBody: Atlantic Ocean; islandGroup: Madeira archipelago; island: Madeira; country: Portugal; countryCode: PT; stateProvince: Madeira; county: Porto Moniz; locality: Rabaçal; verbatimElevation: 930; decimalLatitude: 32.7647; decimalLongitude: -17.1341; geodeticDatum: WGS84; **Event:** samplingProtocol: Direct sampling
- **Type status:** Other material. **Occurrence:** sex: 1 female; **Location:** locationID: 34; higherGeography: Macaronesia; continent: Europe; waterBody: Atlantic Ocean; islandGroup: Madeira archipelago; island: Madeira; country: Portugal; countryCode: PT; stateProvince: Madeira; county: Ponta do Sol; locality: Risco; verbatimElevation: 1048; decimalLatitude: 32.7608; decimalLongitude: -17.1256; geodeticDatum: WGS84; **Event:** samplingProtocol: Direct sampling
- **Type status:** Other material. **Occurrence:** sex: 1 male, 1 female; **Location:** locationID: 35; higherGeography: Macaronesia; continent: Europe; waterBody: Atlantic Ocean; islandGroup: Madeira archipelago; island: Madeira; country: Portugal; countryCode: PT; stateProvince: Madeira; county: Porto Moniz; locality: Casa do Elias; verbatimElevation: 814; decimalLatitude: 32.8268; decimalLongitude: -17.1883; geodeticDatum: WGS84; **Event:** samplingProtocol: Pitfall

##### Ecological interactions

###### Native status

SIE

##### Distribution

Madeira island (Fig. 4d)

##### Notes

Previous records of *Episinus maderianus* were made by Schenkel (Schenkel 1938) and are exclusive to the Laurisilva. The present study greatly widens up the distribution of this endemic species that seems to be widespread within Laurisilva.

#### Paidiscura orotavensis

(Schmidt, 1968)

##### Materials

- **Type status:** Other material. **Occurrence:** sex: 1 female; **Location:** locationID: 11; higherGeography: Macaronesia; continent: Europe; waterBody: Atlantic Ocean; islandGroup: Madeira archipelago; island: Madeira; country: Portugal; countryCode: PT; stateProvince: Madeira; county: Santana; locality: Achada do Teixeira; verbatimElevation: 1211; decimalLatitude: 32.7733; decimalLongitude: -16.9081; geodeticDatum: WGS84; **Event:** samplingProtocol: Pitfall
- **Type status:** Other material. **Occurrence:** sex: 2 females; **Location:** locationID: 18; higherGeography: Macaronesia; continent: Europe; waterBody: Atlantic Ocean; islandGroup: Madeira archipelago; island: Madeira; country: Portugal; countryCode: PT; stateProvince: Madeira; county: Porto Moniz; locality: Chão da Ribeira; verbatimElevation: 491; decimalLatitude: 32.7957; decimalLongitude: -17.1117; geodeticDatum: WGS84; **Event:** samplingProtocol: Direct sampling

##### Ecological interactions

###### Native status

macaronesian endemic

##### Distribution

Madeira archipelago, Canary Islands

#### Rugathodes madeirensis

Wunderlich, 1987

##### Materials

- **Type status:** Other material. **Occurrence:** sex: 1 female; **Location:** locationID: 3; higherGeography: Macaronesia; continent: Europe; waterBody: Atlantic Ocean; islandGroup: Madeira archipelago; island: Madeira; country: Portugal; countryCode: PT; stateProvince: Madeira; county: Santana; locality: Fajã da Nogueira - Pte. Roquete; verbatimElevation: 1074; decimalLatitude: 32.7391; decimalLongitude: -16.9156; geodeticDatum: WGS84; **Event:** samplingProtocol: Pitfall
- **Type status:** Other material. **Occurrence:** sex: 2 females; **Location:** locationID: 4; higherGeography: Macaronesia; continent: Europe; waterBody: Atlantic Ocean; islandGroup: Madeira archipelago; island: Madeira; country: Portugal; countryCode: PT; stateProvince: Madeira; county: Santana; locality: Fajã da Nogueira - Mtdo. do Leacoque; verbatimElevation: 630; decimalLatitude: 32.7415; decimalLongitude: -16.9161; geodeticDatum: WGS84; **Event:** samplingProtocol: Direct sampling
- **Type status:** Other material. **Occurrence:** sex: 1 female; **Location:** locationID: 8; higherGeography: Macaronesia; continent: Europe; waterBody: Atlantic Ocean; islandGroup: Madeira archipelago; island: Madeira; country: Portugal; countryCode: PT; stateProvince: Madeira; county: Santana; locality: Fajã da Nogueira - Til Gigante; verbatimElevation: 841; decimalLatitude: 32.7457; decimalLongitude: -16.915; geodeticDatum: WGS84; **Event:** samplingProtocol: Direct sampling
- **Type status:** Other material. **Occurrence:** sex: 1 female; **Location:** locationID: 9; higherGeography: Macaronesia; continent: Europe; waterBody: Atlantic Ocean; islandGroup: Madeira archipelago; island: Madeira; country: Portugal; countryCode: PT; stateProvince: Madeira; county: Santana; locality: Queimadas; verbatimElevation: 841; decimalLatitude: 32.7873; decimalLongitude: -16.9047; geodeticDatum: WGS84; **Event:** samplingProtocol: Direct sampling
- **Type status:** Other material. **Occurrence:** sex: 1 male; **Location:** locationID: 10; higherGeography: Macaronesia; continent: Europe; waterBody: Atlantic Ocean; islandGroup: Madeira archipelago; island: Madeira; country: Portugal; countryCode: PT; stateProvince: Madeira; county: Santana; locality: Pico das Pedras; verbatimElevation: 883; decimalLatitude: 32.7841; decimalLongitude: -16.9055; geodeticDatum: WGS84; **Event:** samplingProtocol: Direct sampling
- **Type status:** Other material. **Occurrence:** sex: 1 male; **Location:** locationID: 11; higherGeography: Macaronesia; continent: Europe; waterBody: Atlantic Ocean; islandGroup: Madeira archipelago; island: Madeira; country: Portugal; countryCode: PT; stateProvince: Madeira; county: Santana; locality: Achada do Teixeira; verbatimElevation: 1211; decimalLatitude: 32.7733; decimalLongitude: -16.9081; geodeticDatum: WGS84; **Event:** samplingProtocol: Direct sampling
- **Type status:** Other material. **Occurrence:** sex: 1 female; **Location:** locationID: 12; higherGeography: Macaronesia; continent: Europe; waterBody: Atlantic Ocean; islandGroup: Madeira archipelago; island: Madeira; country: Portugal; countryCode: PT; stateProvince: Madeira; county: Santana; locality: Achada do Teixeira; verbatimElevation: 1103; decimalLatitude: 32.7762; decimalLongitude: -16.9022; geodeticDatum: WGS84; **Event:** samplingProtocol: Direct sampling
- **Type status:** Other material. **Occurrence:** sex: 1 female; **Location:** locationID: 12; higherGeography: Macaronesia; continent: Europe; waterBody: Atlantic Ocean; islandGroup: Madeira archipelago; island: Madeira; country: Portugal; countryCode: PT; stateProvince: Madeira; county: Santana; locality: Achada do Teixeira; verbatimElevation: 1103; decimalLatitude: 32.7762; decimalLongitude: -16.9022; geodeticDatum: WGS84; **Event:** samplingProtocol: Pitfall
- **Type status:** Other material. **Occurrence:** sex: 1 female; **Location:** locationID: 13; higherGeography: Macaronesia; continent: Europe; waterBody: Atlantic Ocean; islandGroup: Madeira archipelago; island: Madeira; country: Portugal; countryCode: PT; stateProvince: Madeira; county: Santana; locality: Ribeiro Frio - Viveiro; verbatimElevation: 906; decimalLatitude: 32.7354; decimalLongitude: -16.8864; geodeticDatum: WGS84; **Event:** samplingProtocol: Direct sampling
- **Type status:** Other material. **Occurrence:** sex: 1 female; **Location:** locationID: 17; higherGeography: Macaronesia; continent: Europe; waterBody: Atlantic Ocean; islandGroup: Madeira archipelago; island: Madeira; country: Portugal; countryCode: PT; stateProvince: Madeira; county: Porto Moniz; locality: Chão da Ribeira; verbatimElevation: 519; decimalLatitude: 32.7933; decimalLongitude: -17.1122; geodeticDatum: WGS84; **Event:** samplingProtocol: Pitfall
- **Type status:** Other material. **Occurrence:** sex: 3 females; **Location:** locationID: 22; higherGeography: Macaronesia; continent: Europe; waterBody: Atlantic Ocean; islandGroup: Madeira archipelago; island: Madeira; country: Portugal; countryCode: PT; stateProvince: Madeira; county: Santana; locality: Ribeiro Bonito - Ribeiro; verbatimElevation: 560; decimalLatitude: 32.7985; decimalLongitude: -16.936; geodeticDatum: WGS84; **Event:** samplingProtocol: Direct sampling
- **Type status:** Other material. **Occurrence:** sex: 2 females; **Location:** locationID: 24; higherGeography: Macaronesia; continent: Europe; waterBody: Atlantic Ocean; islandGroup: Madeira archipelago; island: Madeira; country: Portugal; countryCode: PT; stateProvince: Madeira; county: Porto Moniz; locality: Fanal - Levada dos Cedros; verbatimElevation: 820; decimalLatitude: 32.8259; decimalLongitude: -17.158; geodeticDatum: WGS84; **Event:** samplingProtocol: Direct sampling
- **Type status:** Other material. **Occurrence:** sex: 1 female; **Location:** locationID: 28; higherGeography: Macaronesia; continent: Europe; waterBody: Atlantic Ocean; islandGroup: Madeira archipelago; island: Madeira; country: Portugal; countryCode: PT; stateProvince: Madeira; county: Porto Moniz; locality: Fanal; verbatimElevation: 1134; decimalLatitude: 32.8062; decimalLongitude: -17.1409; geodeticDatum: WGS84; **Event:** samplingProtocol: Pitfall
- **Type status:** Other material. **Occurrence:** sex: 1 female; **Location:** locationID: 29; higherGeography: Macaronesia; continent: Europe; waterBody: Atlantic Ocean; islandGroup: Madeira archipelago; island: Madeira; country: Portugal; countryCode: PT; stateProvince: Madeira; county: São Vicente; locality: Ginjas; verbatimElevation: 869; decimalLatitude: 32.7758; decimalLongitude: -17.0534; geodeticDatum: WGS84; **Event:** samplingProtocol: Direct sampling

##### Ecological interactions

###### Native status

SIE

##### Distribution

Madeira island (Fig. 5a)

##### Notes

This species has been found mostly in Laurisilva. Its occurence outside the largest areas of native forest may be explained by the existence of small, secluded micro-habitats composed of native flora, which might serve as ecological islands for forest species.

#### Steatoda nobilis

(Thorell, 1875)

##### Materials

- **Type status:** Other material. **Occurrence:** sex: 1 female; **Location:** locationID: 4; higherGeography: Macaronesia; continent: Europe; waterBody: Atlantic Ocean; islandGroup: Madeira archipelago; island: Madeira; country: Portugal; countryCode: PT; stateProvince: Madeira; county: Santana; locality: Fajã da Nogueira - Mtdo. do Leacoque; verbatimElevation: 630; decimalLatitude: 32.7415; decimalLongitude: -16.9161; geodeticDatum: WGS84; **Event:** samplingProtocol: Direct sampling

##### Ecological interactions

###### Native status

native

##### Distribution

Madeira archipelago, Canary Islands (elsewhere, introduced)

#### Theridion melanurum

Hahn, 1831

##### Materials

- **Type status:** Other material. **Occurrence:** sex: 2 females; **Location:** locationID: 4; higherGeography: Macaronesia; continent: Europe; waterBody: Atlantic Ocean; islandGroup: Madeira archipelago; island: Madeira; country: Portugal; countryCode: PT; stateProvince: Madeira; county: Santana; locality: Fajã da Nogueira - Mtdo. do Leacoque; verbatimElevation: 630; decimalLatitude: 32.7415; decimalLongitude: -16.9161; geodeticDatum: WGS84; **Event:** samplingProtocol: Direct sampling
- **Type status:** Other material. **Occurrence:** sex: 1 female; **Location:** locationID: 7; higherGeography: Macaronesia; continent: Europe; waterBody: Atlantic Ocean; islandGroup: Madeira archipelago; island: Madeira; country: Portugal; countryCode: PT; stateProvince: Madeira; county: Santana; locality: Fajã da Nogueira - Tanque; verbatimElevation: 845; decimalLatitude: 32.7425; decimalLongitude: -16.9168; geodeticDatum: WGS84; **Event:** samplingProtocol: Direct sampling
- **Type status:** Other material. **Occurrence:** sex: 1 female; **Location:** locationID: 9; higherGeography: Macaronesia; continent: Europe; waterBody: Atlantic Ocean; islandGroup: Madeira archipelago; island: Madeira; country: Portugal; countryCode: PT; stateProvince: Madeira; county: Santana; locality: Queimadas; verbatimElevation: 841; decimalLatitude: 32.7873; decimalLongitude: -16.9047; geodeticDatum: WGS84; **Event:** samplingProtocol: Direct sampling
- **Type status:** Other material. **Occurrence:** sex: 1 male, 3 females; **Location:** locationID: 11; higherGeography: Macaronesia; continent: Europe; waterBody: Atlantic Ocean; islandGroup: Madeira archipelago; island: Madeira; country: Portugal; countryCode: PT; stateProvince: Madeira; county: Santana; locality: Achada do Teixeira; verbatimElevation: 1211; decimalLatitude: 32.7733; decimalLongitude: -16.9081; geodeticDatum: WGS84; **Event:** samplingProtocol: Direct sampling
- **Type status:** Other material. **Occurrence:** sex: 1 female; **Location:** locationID: 14; higherGeography: Macaronesia; continent: Europe; waterBody: Atlantic Ocean; islandGroup: Madeira archipelago; island: Madeira; country: Portugal; countryCode: PT; stateProvince: Madeira; county: Santana; locality: Ribeiro Frio - Cottages; verbatimElevation: 994; decimalLatitude: 32.7319; decimalLongitude: -16.8861; geodeticDatum: WGS84; **Event:** samplingProtocol: Direct sampling
- **Type status:** Other material. **Occurrence:** sex: 1 female; **Location:** locationID: 15; higherGeography: Macaronesia; continent: Europe; waterBody: Atlantic Ocean; islandGroup: Madeira archipelago; island: Madeira; country: Portugal; countryCode: PT; stateProvince: Madeira; county: Santana; locality: Pico do Areeiro; verbatimElevation: 1533; decimalLatitude: 32.7231; decimalLongitude: -16.9109; geodeticDatum: WGS84; **Event:** samplingProtocol: Direct sampling
- **Type status:** Other material. **Occurrence:** sex: 2 females; **Location:** locationID: 26; higherGeography: Macaronesia; continent: Europe; waterBody: Atlantic Ocean; islandGroup: Madeira archipelago; island: Madeira; country: Portugal; countryCode: PT; stateProvince: Madeira; county: Porto Moniz; locality: Fanal; verbatimElevation: 889; decimalLatitude: 32.8226; decimalLongitude: -17.1539; geodeticDatum: WGS84; **Event:** samplingProtocol: Direct sampling
- **Type status:** Other material. **Occurrence:** sex: 1 female; **Location:** locationID: 27; higherGeography: Macaronesia; continent: Europe; waterBody: Atlantic Ocean; islandGroup: Madeira archipelago; island: Madeira; country: Portugal; countryCode: PT; stateProvince: Madeira; county: Porto Moniz; locality: Fanal; verbatimElevation: 1023; decimalLatitude: 32.8182; decimalLongitude: -17.1521; geodeticDatum: WGS84; **Event:** samplingProtocol: Direct sampling

##### Ecological interactions

###### Native status

introduced

##### Distribution

Holarctic, Azores, Madeira

#### Theridion n. sp.

##### Materials

- **Type status:** Other material. **Occurrence:** sex: 1 male, 1 female; **Location:** locationID: 1; higherGeography: Macaronesia; continent: Europe; waterBody: Atlantic Ocean; islandGroup: Madeira archipelago; island: Madeira; country: Portugal; countryCode: PT; stateProvince: Madeira; county: Machico; locality: Funduras; verbatimElevation: 500; decimalLatitude: 32.7493; decimalLongitude: -16.8114; geodeticDatum: WGS84; **Event:** samplingProtocol: Direct sampling
- **Type status:** Other material. **Occurrence:** sex: 1 female; **Location:** locationID: 2; higherGeography: Macaronesia; continent: Europe; waterBody: Atlantic Ocean; islandGroup: Madeira archipelago; island: Madeira; country: Portugal; countryCode: PT; stateProvince: Madeira; county: Machico; locality: Funduras; verbatimElevation: 552; decimalLatitude: 32.754; decimalLongitude: -16.8099; geodeticDatum: WGS84; **Event:** samplingProtocol: Direct sampling
- **Type status:** Other material. **Occurrence:** sex: 1 male; **Location:** locationID: 4; higherGeography: Macaronesia; continent: Europe; waterBody: Atlantic Ocean; islandGroup: Madeira archipelago; island: Madeira; country: Portugal; countryCode: PT; stateProvince: Madeira; county: Santana; locality: Fajã da Nogueira - Mtdo. do Leacoque; verbatimElevation: 630; decimalLatitude: 32.7415; decimalLongitude: -16.9161; geodeticDatum: WGS84; **Event:** samplingProtocol: Direct sampling
- **Type status:** Other material. **Occurrence:** sex: 1 male; **Location:** locationID: 13; higherGeography: Macaronesia; continent: Europe; waterBody: Atlantic Ocean; islandGroup: Madeira archipelago; island: Madeira; country: Portugal; countryCode: PT; stateProvince: Madeira; county: Santana; locality: Ribeiro Frio - Viveiro; verbatimElevation: 906; decimalLatitude: 32.7354; decimalLongitude: -16.8864; geodeticDatum: WGS84; **Event:** samplingProtocol: Direct sampling
- **Type status:** Other material. **Occurrence:** sex: 1 female; **Location:** locationID: 14; higherGeography: Macaronesia; continent: Europe; waterBody: Atlantic Ocean; islandGroup: Madeira archipelago; island: Madeira; country: Portugal; countryCode: PT; stateProvince: Madeira; county: Santana; locality: Ribeiro Frio - Cottages; verbatimElevation: 994; decimalLatitude: 32.7319; decimalLongitude: -16.8861; geodeticDatum: WGS84; **Event:** samplingProtocol: Direct sampling
- **Type status:** Other material. **Occurrence:** sex: 1 male; **Location:** locationID: 18; higherGeography: Macaronesia; continent: Europe; waterBody: Atlantic Ocean; islandGroup: Madeira archipelago; island: Madeira; country: Portugal; countryCode: PT; stateProvince: Madeira; county: Porto Moniz; locality: Chão da Ribeira; verbatimElevation: 491; decimalLatitude: 32.7957; decimalLongitude: -17.1117; geodeticDatum: WGS84; **Event:** samplingProtocol: Direct sampling
- **Type status:** Other material. **Occurrence:** sex: 1 female; **Location:** locationID: 21; higherGeography: Macaronesia; continent: Europe; waterBody: Atlantic Ocean; islandGroup: Madeira archipelago; island: Madeira; country: Portugal; countryCode: PT; stateProvince: Madeira; county: Santana; locality: Ribeiro Bonito - Levada; verbatimElevation: 568; decimalLatitude: 32.8047; decimalLongitude: -16.9346; geodeticDatum: WGS84; **Event:** samplingProtocol: Direct sampling
- **Type status:** Other material. **Occurrence:** sex: 1 male; **Location:** locationID: 24; higherGeography: Macaronesia; continent: Europe; waterBody: Atlantic Ocean; islandGroup: Madeira archipelago; island: Madeira; country: Portugal; countryCode: PT; stateProvince: Madeira; county: Porto Moniz; locality: Fanal - Levada dos Cedros; verbatimElevation: 820; decimalLatitude: 32.8259; decimalLongitude: -17.158; geodeticDatum: WGS84; **Event:** samplingProtocol: Direct sampling
- **Type status:** Other material. **Occurrence:** sex: 1 male, 1 female; **Location:** locationID: 33; higherGeography: Macaronesia; continent: Europe; waterBody: Atlantic Ocean; islandGroup: Madeira archipelago; island: Madeira; country: Portugal; countryCode: PT; stateProvince: Madeira; county: Porto Moniz; locality: Rabaçal; verbatimElevation: 930; decimalLatitude: 32.7647; decimalLongitude: -17.1341; geodeticDatum: WGS84; **Event:** samplingProtocol: Direct sampling
- **Type status:** Other material. **Occurrence:** sex: 1 male; **Location:** locationID: 34; higherGeography: Macaronesia; continent: Europe; waterBody: Atlantic Ocean; islandGroup: Madeira archipelago; island: Madeira; country: Portugal; countryCode: PT; stateProvince: Madeira; county: Ponta do Sol; locality: Risco; verbatimElevation: 1048; decimalLatitude: 32.7608; decimalLongitude: -17.1256; geodeticDatum: WGS84; **Event:** samplingProtocol: Direct sampling

##### Ecological interactions

###### Native status

SIE

##### Distribution

Madeira island (Fig. 5b)

##### Notes

This is a new species to science. It seems to be restricted to Laurisilva and is now being formally described (Van Keer, in prep.).

#### Misumena spinifera

(Blackwall, 1862)

##### Materials

- **Type status:** Other material. **Occurrence:** sex: 1 male; **Location:** locationID: 25; higherGeography: Macaronesia; continent: Europe; waterBody: Atlantic Ocean; islandGroup: Madeira archipelago; island: Madeira; country: Portugal; countryCode: PT; stateProvince: Madeira; county: Porto Moniz; locality: Fanal; verbatimElevation: 890; decimalLatitude: 32.8236; decimalLongitude: -17.156; geodeticDatum: WGS84; **Event:** samplingProtocol: Direct sampling

##### Ecological interactions

###### Native status

macaronesian endemic

##### Distribution

Madeira island, Canary Islands

#### Xysticus nubilus

Simon, 1875

##### Materials

- **Type status:** Other material. **Occurrence:** sex: 2 females; **Location:** locationID: 15; higherGeography: Macaronesia; continent: Europe; waterBody: Atlantic Ocean; islandGroup: Madeira archipelago; island: Madeira; country: Portugal; countryCode: PT; stateProvince: Madeira; county: Santana; locality: Pico do Areeiro; verbatimElevation: 1533; decimalLatitude: 32.7231; decimalLongitude: -16.9109; geodeticDatum: WGS84; **Event:** samplingProtocol: Pitfall

##### Ecological interactions

###### Native status

introduced

##### Distribution

Mediterranean, Macaronesia

#### Zodarion styliferum

(Simon, 1870)

##### Materials

- **Type status:** Other material. **Occurrence:** sex: 201 males, 63 females; **Location:** locationID: 15; higherGeography: Macaronesia; continent: Europe; waterBody: Atlantic Ocean; islandGroup: Madeira archipelago; island: Madeira; country: Portugal; countryCode: PT; stateProvince: Madeira; county: Santana; locality: Pico do Areeiro; verbatimElevation: 1533; decimalLatitude: 32.7231; decimalLongitude: -16.9109; geodeticDatum: WGS84; **Event:** samplingProtocol: Pitfall
- **Type status:** Other material. **Occurrence:** sex: 1 male; **Location:** locationID: 32; higherGeography: Macaronesia; continent: Europe; waterBody: Atlantic Ocean; islandGroup: Madeira archipelago; island: Madeira; country: Portugal; countryCode: PT; stateProvince: Madeira; county: Calheta; locality: Rabaças; verbatimElevation: 993; decimalLatitude: 32.7413; decimalLongitude: -17.0783; geodeticDatum: WGS84; **Event:** samplingProtocol: Direct sampling
- **Type status:** Other material. **Occurrence:** sex: 14 males, 7 females; **Location:** locationID: 32; higherGeography: Macaronesia; continent: Europe; waterBody: Atlantic Ocean; islandGroup: Madeira archipelago; island: Madeira; country: Portugal; countryCode: PT; stateProvince: Madeira; county: Calheta; locality: Rabaças; verbatimElevation: 993; decimalLatitude: 32.7413; decimalLongitude: -17.0783; geodeticDatum: WGS84; **Event:** samplingProtocol: Pitfall

##### Ecological interactions

###### Native status

introduced

##### Distribution

Iberian Peninsula, Madeira island, Porto Santo island (introduced in the islands)

## Discussion

Fifty two spider species and 3145 specimens are accounted in this study. Out of these, 17 are Madeira’s endemics species (SIE) mostly restricted to Laurisilva. Range for most endemic spider species was expanded by this study (Figs 1, 2, 3, 4, 5) and six endemics [*Hahnia insulana* Schenkel, 1938, *Frontinellina dearmata* (Kulczynski, 1899), *Lepthyphantes mauli* Wunderlich, 1992, *Trogloneta madeirensis* Wunderlich, 1987, *Metastridulans* Wunderlich, 1987 and *Dipoenata longitarsis* (Denis, 1962)], were collected for the first time after their original description. A major finding of this study was the discovery of two species new to science, a species of *Ceratinopsis* Emerton, 1882, and a species of *Theridion* Walckenaer, 1805, apparently restricted to Laurisilva. The remaining thirty five spider species were assigned to one of the two following categories: non-endemic native (for both macaronesian endemics and species with a wider distribution) and introduced species. The inclusion of each species in these categories was based on distribution and species ecological traits. Sixteen species were classified as introduced species, including *Poeciloneta variegata* (Blackwall, 1841) and *Tetragnatha intermedia* Kulczynski, 1891, two new records for Madeira island. Two species with uncertain identity, *Macaroeris* sp. and “Linyphiidae unidentified species” were considered as non-endemic native species.

Species richness considerations

**Figure 7.**
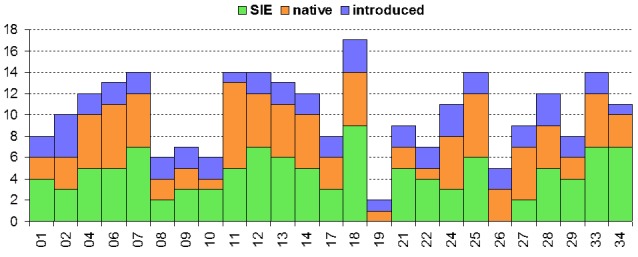
Species richness values per site, highlighting the relative proportion of endemic, native and introduced species. The data of each site combines information from both pitfall and direct sampling. Suppl. material 1.

**Figure 8.**
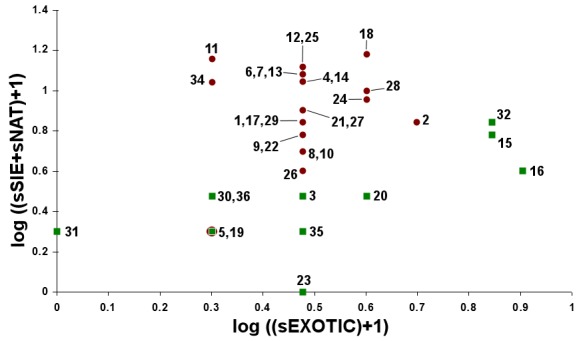
Scatter plot of the log-transformed species richness of endemic and native species versus exotics for each sampling site. Red circles – from both pitfall trapping and direct sampling; Green squares – pitfall data only. Suppl. material 1.

The highest values of species richness were found in sites 18, 7, 11, 12, 25 and 33, which were also among those with the largest number of endemics (Fig. 7, Table 2). The lowest species richness was recorded in site 31, with only 1 native species. Abundance was maximum on alpine meadow sites (15, 16), mostly due to the presence of several abundant species like *Zodarion styliferum*, *Haplodrassus dalmatensis* and *Zelotes civicus*. These ground-dwelling species are quite vagile and are among the top invertebrate predators of Madeiran altitudinal meadows. In most study areas, introduced species were more abundant than natives, with some exceptions, such as sites 11, 12, 13, 18, 25 and 34, where higher proportions of specimens from endemic species were recorded (Fig. 8). The lowest proportions of native and endemic species were recorded for sites 5, 19, 23, 31 and 35, where site 19 was the only sampled by both sampling techniques.

This study reveals a high number of endemic spider species in some locations, such as Chão da Ribeira (sites 17 and 18), Rabaçal (site 33), Risco (site 34), Achada do Teixeira (sites 11 and 12), Ribeiro Frio (sites 13 and 14), Fajã da Nogueira (sites 3 to 8) and Fanal (sites 23 to 28) that should therefore be the focus of further conservation biology of Madeiran spiders and their associated habitats. Those sites are the only where *Centromerus variegatus*, *Ceratinopsis infuscata*, *Dipoenata longitarsis*, *Frontinellina dearmata*, *Frontiphantes fulgurenotatus* and *Lepthyphantes mauli* are known to occur. However, the occurrence of these araneoid spiders in other locations within Madeira island is expected since they have a potentially high dispersal ability.

This is the first time that a large set of standardized samples of spiders from Madeira island is reported. The distribution of several Madeiran endemic species was greatly increased, particularly *Macarophaeus cultior*, *Hahnia insulana*, *Turinyphia maderiana*, *Trogloneta madeirensis*, *Rugathodes madeirensis* and *Episinus maderianus*. Other endemics were recorded in fewer sites, but in most cases such species are still associated with Laurisilva. In some instances, the historical distribution of endemic spider species includes areas no longer covered by Laurisilva. An example is a record of *Centromerus variegatus* from Santo da Serra (Denis 1962), a site now covered by exotic forest but which was presumably dominated by endemic *Erica* shrubs just some decades ago.

The need to apply a combination of sampling techniques in a standardized design in order to assess species composition and structure of ecological communities has been strongly emphasized in the last few years by different authors (Borges and Wunderlich (2008), Borges et al. (2005), Borges et al. (2006), Cardoso (2009), Cardoso et al. (2009)). Accordingly, combining two complementary sampling techniques was determinant in our study to find a variety of spider species that use the habitat in different ways.

In complex habitats like Laurisilva, the prospection of microhabitats such as fallen logs and trees, tree holes, under tree bark and epiphytes allows the finding of species with highly specialized niches. Furthermore, several spider species seldom move or do not move at all at ground level, making direct sampling the most suitable detection technique. From the 52 spider species reported, 16 were collected exclusively using pitfall traps while 22 other species were only found by direct sampling. On the other hand, direct sampling proved to be an effective method to sample Madeiran endemics since most of the sampled endemic spider species were only collected using this technique (see also Fig. 8).

The original descriptions of some Madeiran endemics do not allow inequivocal identification since they were based on very few specimens or on specimens of a single gender. Thus, taxonomic revisions of several genera or families (i.e. *Dysdera* Latreille, 1804, *Hahnia* C.L. Koch, 1841, or the Linyphiidae) are mandatory for a better understanding of Madeiran spider biodiversity. Besides the merits of the present study in improving the knowledge on the distribution of spider species in Madeira island, it also allowed the collection of a number of specimens that will be the aim of future taxonomic studies, such as species redescriptions and formal descriptions of new species to science.

Madeira Laurisilva presents a unique spider biodiversity and efforts should be made towards improving knowledge on the abundance and distribution of endemic species as well as to develop a strategy for their conservation and monitoring.

## Supplementary Material

Supplementary material 1 Species richness data Data type: occurrences
Brief description: Data from where Figures 5 and 6 were built.
File: oo_5544.xls Luís Crespo

XML Treatment for Zygiella minima

XML Treatment for Clubiona decora

XML Treatment for Lathys affinis

XML Treatment for Nigma puella

XML Treatment for Haplodrassus dalmatensis

XML Treatment for Macarophaeus cultior

XML Treatment for Micaria pallipes

XML Treatment for Zelotes civicus

XML Treatment for Hahnia insulana

XML Treatment for Centromerus variegatus

XML Treatment for Ceratinopsis acripes

XML Treatment for Ceratinopsis infuscata

XML Treatment for Ceratinopsis n. sp.

XML Treatment for Diplostyla concolor

XML Treatment for Entelecara schmitzi

XML Treatment for Frontinellina dearmata

XML Treatment for Frontiphantes fulgurenotatus

XML Treatment for Lepthyphantes impudicus

XML Treatment for Lepthyphantes mauli

XML Treatment for Meioneta fuscipalpa

XML Treatment for Microctenonyx subitaneus

XML Treatment for Microlinyphia johnsoni

XML Treatment for Palliduphantes schmitzi

XML Treatment for Poeciloneta variegata

XML Treatment for Tenuiphantes tenuis

XML Treatment for Turinyphia maderiana

XML Treatment for Typhochrestus madeirensis

XML Treatment for Pardosa proxima

XML Treatment for Ero aphana

XML Treatment for Cheiracanthium albidulum

XML Treatment for Trogloneta madeirensis

XML Treatment for Philodromus insulanus

XML Treatment for Thanatus oblongiusculus

XML Treatment for Chalcoscirtus sublestus

XML Treatment for Macaroeris diligens

XML Treatment for Macaroeris sp.

XML Treatment for Pellenes maderianus

XML Treatment for Meta stridulans

XML Treatment for Tetragnatha intermedia

XML Treatment for Cryptachaea blattea

XML Treatment for Dipoenata cf. longitarsis

XML Treatment for Enoplognatha sattleri

XML Treatment for Episinus maderianus

XML Treatment for Paidiscura orotavensis

XML Treatment for Rugathodes madeirensis

XML Treatment for Steatoda nobilis

XML Treatment for Theridion melanurum

XML Treatment for Theridion n. sp.

XML Treatment for Misumena spinifera

XML Treatment for Xysticus nubilus

XML Treatment for Zodarion styliferum

## Figures and Tables

**Figure 1a.**
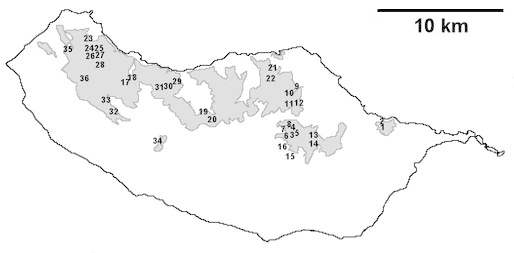
sampling sites in Madeira island. Laurisilva cover shaded grey. Sites 15 and 16 are not in Laurisilva;

**Figure 1b.**
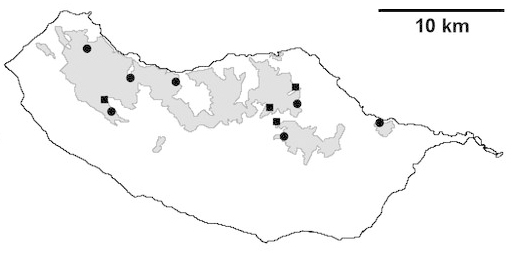
distribution of *Macarophaeus cultior*;

**Figure 1c.**
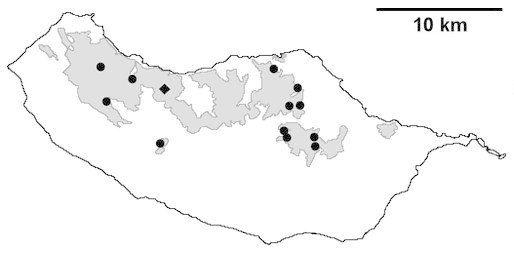
distribution of *Hahnia insulana*;

**Figure 1d.**
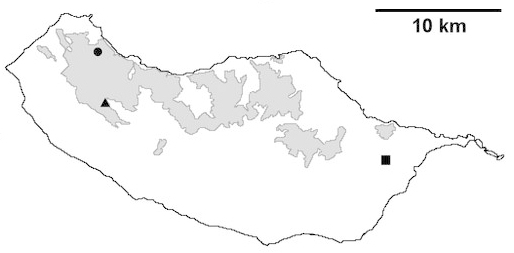
distribution of *Centromerus variegatus*.

**Figure 2a.**
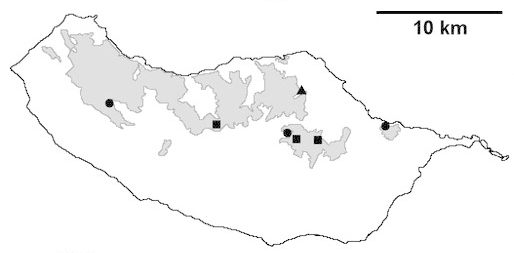
*Ceratinopsis acripes*

**Figure 2b.**
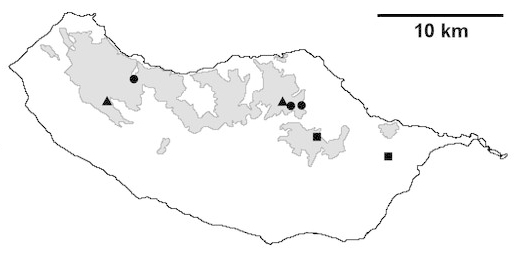
*Ceratinopsis infuscata*

**Figure 2c.**
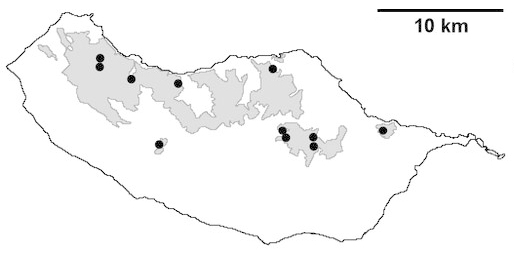
*Ceratinopsis* n. sp.

**Figure 2d.**
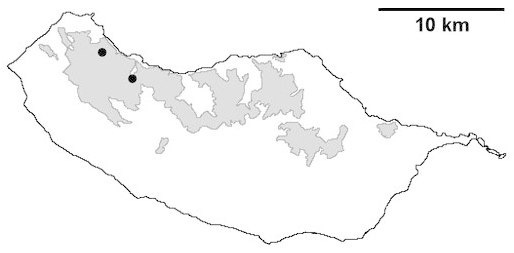
*Frontinellina dearmata*

**Figure 3a.**
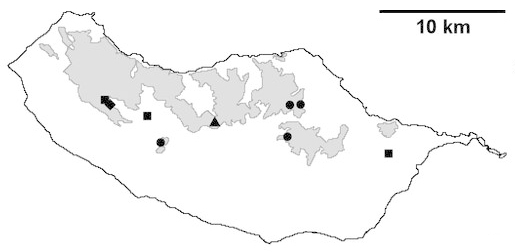
*Frontiphantes fulgurenotatus*;

**Figure 3b.**
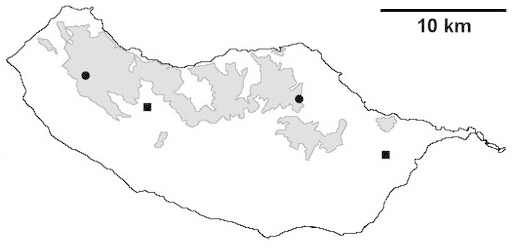
*Lepthyphantes impudicus*;

**Figure 3c.**
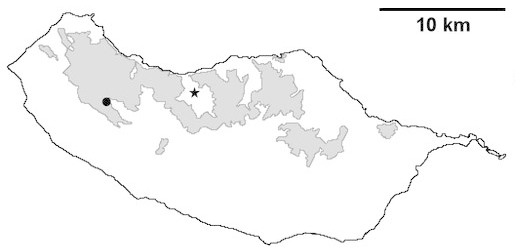
*Lepthyphantes mauli*;

**Figure 3d.**
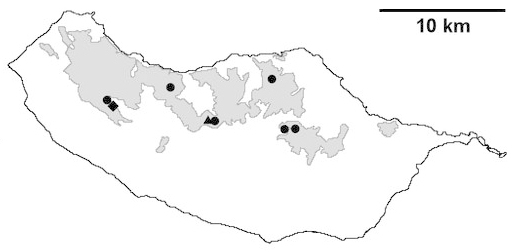
*Turinyphia maderiana*.

**Figure 4a.**
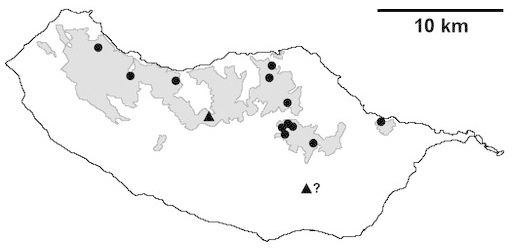
*Trogloneta madeirensis*;

**Figure 4b.**
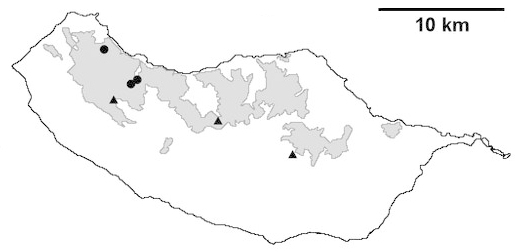
*Meta stridulans*;

**Figure 4c.**
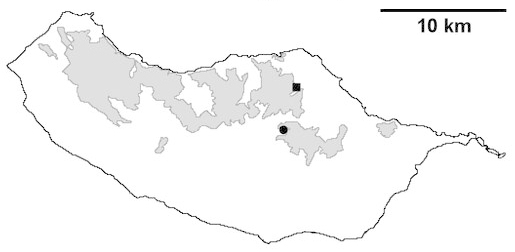
*Dipoenata longitarsis*;

**Figure 4d.**
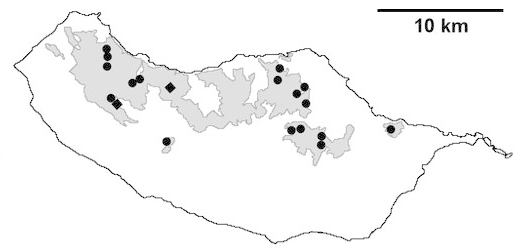
*Episinus maderianus*.

**Figure 5a.**
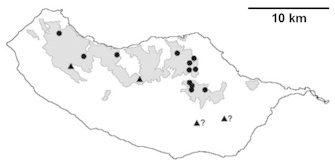
*Rugathodes madeirensis*;

**Figure 5b.**
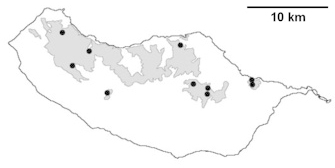
*Theridion* n. sp.

**Figure 6a.**
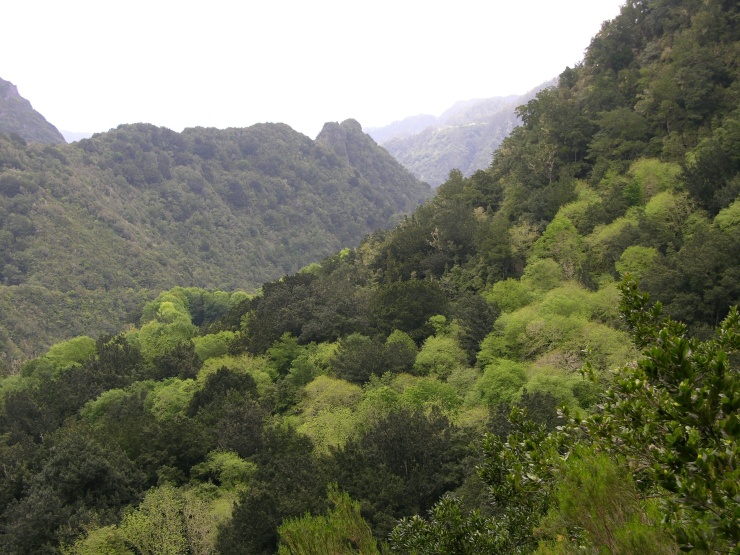
panoramic view of Fajã da Nogueira;

**Figure 6b.**
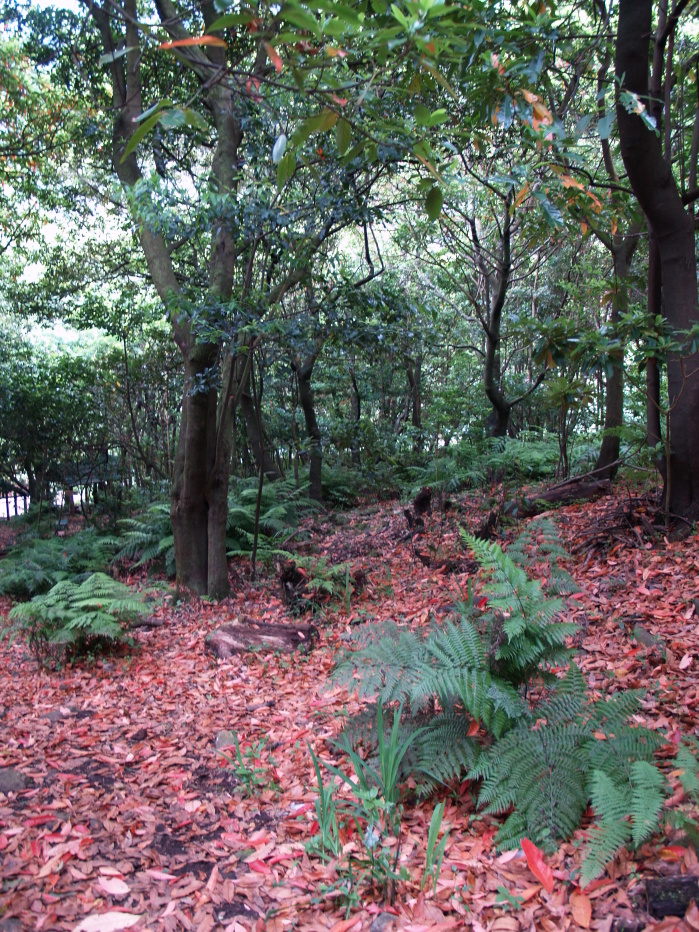
sampling site in Chão da Ribeira;

**Figure 6c.**
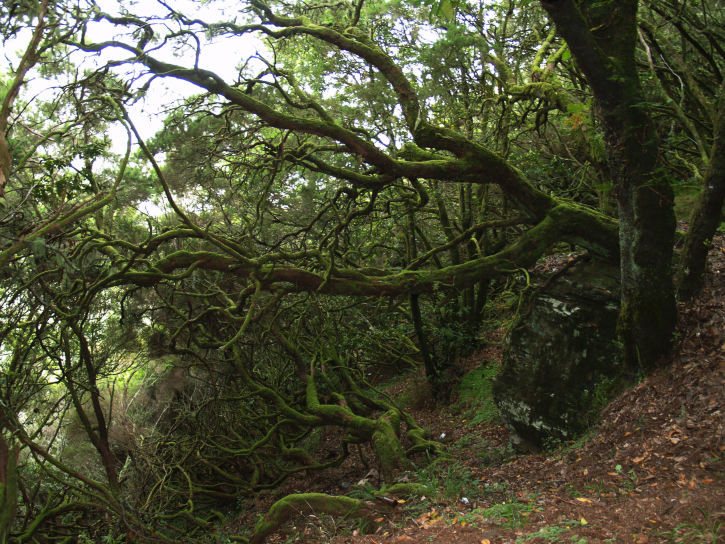
sampling site in Rabaçal;

**Figure 6d.**
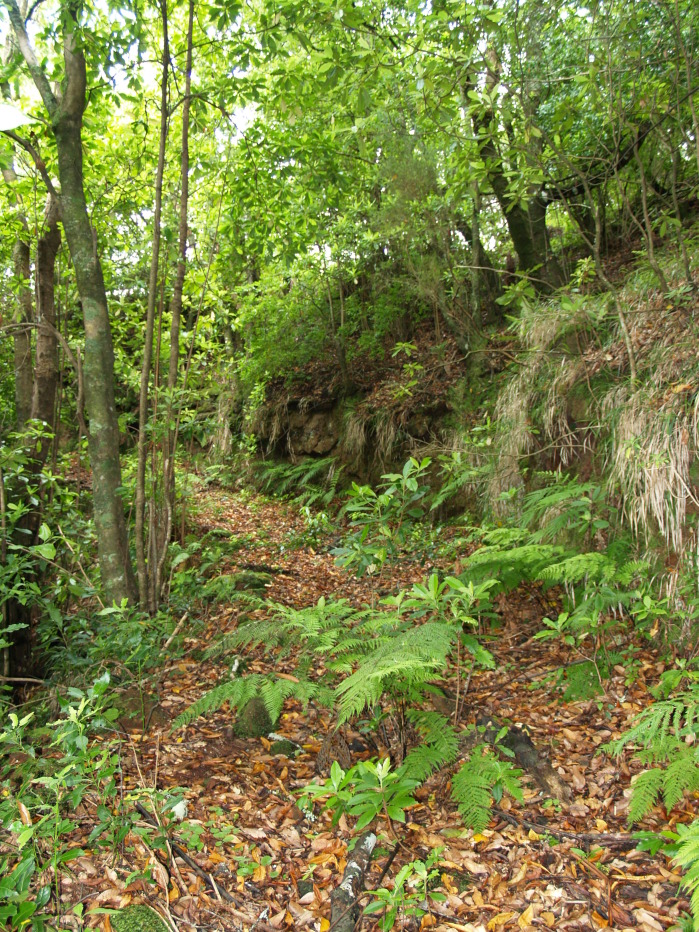
sampling site in Galhano.

**Table 1.** Study sites, sampling date/period and collecting method. Geographic coordinates in decimal degrees.

Study sites					Sampling	
Code	Site	Lat. (N)	Long. (W)	Altitude (m)	Pitfall trapping period	Direct sampling period
1	Funduras	32.7493	-16.8114	500	22.V-05.VI.2006	04.VI.2007
2	Funduras	32.7540	-16.8099	552	22.V-05.VI.2006	04.VI.2007
3	Fajã da Nogueira – Levada Pte. Roquete	32.7391	-16.9156	1074	23.V-06.VI.2006	-
4	Fajã da Nogueira – Mtdo. do Leacoque	32.7415	-16.9161	630	23.V-06.VI.2006	06.VI.2007
5	Fajã da Nogueira – Casa do Levadeiro	32.7406	-16.9136	989	23.V-06.VI.2006	-
6	Fajã da Nogueira – Mtdo. do Leacoque	32.7418	-16.9177	614	23.V-06.VI.2006	06.VI.2007
7	Fajã da Nogueira – Tanque	32.7425	-16.9168	845	23.V-06.VI.2006	06.VI.2007
8	Fajã da Nogueira – Til Gigante	32.7457	-16.9150	841	23.V-06.VI.2006	06.VI.2007
9	Queimadas	32.7873	-16.9047	841	24.V-07.VI.2006	05.VI.2007
10	Pico das Pedras	32.7841	-16.9055	883	24.V-07.VI.2006	05.VI.2007
11	Achada do Teixeira	32.7733	-16.9081	1211	24.V-07.VI.2006	05.VI.2007
12	Achada do Teixeira	32.7762	-16.9022	1103	24.V-07.VI.2006	05.VI.2007
13	Ribeiro Frio – Viveiro	32.7354	-16.8864	906	24.V-06.VI.2006	05.VI.2007
14	Ribeiro Frio – Cottages	32.7319	-16.8861	994	24.V-07.VI.2006	06.VI.2007
15	Pico do Areeiro	32.7231	-16.9109	1533	24.V-07.VI.2006	-
16	Pico do Areeiro	32.7287	-16.9202	1594	24.V-07.VI.2006	-
17	Chão da Ribeira	32.7933	-17.1122	519	25.V-08.VI.2006	07.VI.2007
18	Chão da Ribeira	32.7957	-17.1117	491	25.V-08.VI.2006	07.VI.2007
19	Chão dos Louros	32.7636	-17.0190	748	25.V-08.VI.2006	07.VI.2007
20	Encumeada	32.7558	-17.0143	999	25.V-08.VI.2006	-
21	Ribeiro Bonito – Levada	32.8047	-16.9346	568	25.V-08.VI.2006	09.VI.2007
22	Ribeiro Bonito – Ribeiro	32.7985	-16.9360	560	25.V-08.VI.2006	09.VI.2007
23	Fanal	32.8302	-17.1585	755	26.V-09.VI.2006	-
24	Fanal – Levada dos Cedros	32.8259	-17.1580	820	26.V-09.VI.2006	09.VI.2007
25	Fanal	32.8236	-17.1560	890	26.V-09.VI.2006	09.VI.2007
26	Fanal	32.8226	-17.1539	889	26.V-09.VI.2006	09.VI.2007
27	Fanal	32.8182	-17.1521	1023	26.V-09.VI.2006	09.VI.2007
28	Fanal	32.8062	-17.1409	1134	26.V-09.VI.2006	10.VI.2007
29	Ginjas	32.7758	-17.0534	869	08-22.VI.2007	23.VI.2007
30	Caramujo	32.7722	-17.0529	981	08-22.VI.2007	-
31	Caramujo	32.7746	-17.0559	1001	08-22.VI.2007	-
32	Rabaças	32.7413	-17.0783	993	10-24.VI.2007	-
33	Rabaçal	32.7647	-17.1341	930	10-24.VI.2007	25.VI.2007
34	Risco	32.7608	-17.1256	1048	10-24.VI.2007	25.VI.2007
35	Casa do Elias	32.8268	-17.1883	814	26.VI-10.VII.2007	-
36	Galhano	32.7971	-17.1729	975	27.VI-11.VII.2007	-

**Table 2.** Species richness and abundance per study site. Sampling methods used, number of endemic (SIE), native and introduced species are presented for each site. Site codes as in Table 1.

Site	Species richness (S)				Abundance (N)				Sampling methods
**Code**	SIE	Native	Invasive	**total**	SIE	Native	Invasive	**total**	
**1**	4	2	2	**8**	7	3	24	**34**	Pitfall & direct sampling
**2**	3	3	4	**10**	4	12	63	**79**	Pitfall & direct sampling
**3**	1	1	2	**2**	1	1	2	**4**	Pitfall
**4**	5	4	3	**12**	13	8	38	**59**	Pitfall & direct sampling
**5**	0	1	1	**2**	0	3	1	**4**	Pitfall
**6**	5	6	2	**13**	12	7	36	**55**	Pitfall & direct sampling
**7**	7	4	3	**13**	10	6	21	**37**	Pitfall & direct sampling
**8**	2	2	2	**6**	2	2	19	**23**	Pitfall & direct sampling
**9**	3	1	3	**7**	3	3	10	**16**	Pitfall & direct sampling
**10**	3	1	2	**6**	4	4	9	**17**	Pitfall & direct sampling
**11**	5	7	2	**14**	5	25	17	**47**	Pitfall & direct sampling
**12**	7	5	2	**14**	12	11	8	**31**	Pitfall & direct sampling
**13**	6	5	2	**13**	9	10	7	**26**	Pitfall & direct sampling
**14**	5	4	3	**12**	7	4	11	**22**	Pitfall & direct sampling
**15**	0	4	7	**11**	0	28	364	**392**	Pitfall
**16**	0	3	7	**10**	0	45	215	**260**	Pitfall
**17**	3	3	2	**8**	4	4	14	**22**	Pitfall & direct sampling
**18**	9	5	3	**16**	17	17	25	**59**	Pitfall & direct sampling
**19**	0	1	1	**2**	0	3	3	**6**	Pitfall & direct sampling
**20**	1	1	3	**5**	1	2	13	**16**	Pitfall
**21**	5	2	2	**9**	17	3	37	**57**	Pitfall & direct sampling
**22**	4	1	2	**7**	8	1	20	**29**	Pitfall & direct sampling
**23**	0	0	2	**2**	0	0	5	**5**	Pitfall
**24**	3	5	3	**11**	7	22	22	**51**	Pitfall & direct sampling
**25**	6	6	2	**8**	11	12	19	**42**	Pitfall & direct sampling
**26**	0	2	3	**5**	0	7	7	**14**	Pitfall & direct sampling
**27**	2	4	3	**9**	3	7	9	**19**	Pitfall & direct sampling
**28**	5	4	3	**12**	5	9	15	**29**	Pitfall & direct sampling
**29**	4	2	2	**8**	6	5	11	**22**	Pitfall & direct sampling
**30**	1	1	1	**3**	1	2	2	**5**	Pitfall
**31**	0	1	0	**1**	0	1	0	**1**	Pitfall
**32**	0	6	6	**12**	0	11	49	**60**	Pitfall
**33**	7	5	2	**14**	11	7	17	**35**	Pitfall & direct sampling
**34**	7	3	1	**11**	9	3	8	**20**	Pitfall & direct sampling
**35**	1	0	2	**3**	2	0	5	**7**	Pitfall
**36**	1	1	1	**3**	1	2	12	**15**	Pitfall
